# Transcriptional profiling of leukocytes in critically ill COVID19 patients: implications for interferon response and coagulation

**DOI:** 10.1186/s40635-020-00361-9

**Published:** 2020-12-11

**Authors:** Sean E. Gill, Claudia C. dos Santos, David B. O’Gorman, David E. Carter, Eric K. Patterson, Marat Slessarev, Claudio Martin, Mark Daley, Michael R. Miller, Gediminas Cepinskas, Douglas D. Fraser, Lawson COVID19 Study Team, Robert Arntfield, Ian Ball, Gordon Barkwell, Tracey Bentall, Karen Bosma, Saoirse Cameron, Eileen Campbell, Carolina Gillio-Meina, Robert Hegele, Natalya Odoardi, Ram Singh, Kelly Summers, Sue Tereschyn

**Affiliations:** 1 Lawson Health Research Institute, London, ON Canada; 2 Physiology and Pharmacology, Western University, London, ON Canada; 3 Medicine, Western University, London, ON Canada; 4 Interdepartmental Division of Critical Care Medicine and Keenan Center for Biomedical Research of St. Michael’s Hospital, University of Toronto, Toronto, ON Canada; 5 Biochemistry, Western University, London, ON Canada; 6 London Regional Genomics Centre, Western University, London, ON Canada; 7 Computer Science, Western University, London, ON Canada; 8 Pediatrics, Western University, London, ON Canada; 9 Medical Biophysics, Western University, London, ON Canada; 10 Victoria Research Labs, Room A6-134, 800 Commissioners Road East, London, ON N6A 5W9 Canada; 11 London Health Sciences Centre, Room C2-C82, 800 Commissioners Road East, London, ON N6A 5W9 Canada

**Keywords:** COVID19, RNAseq, Leukocyte transcriptome

## Abstract

**Background:**

COVID19 is caused by the SARS-CoV-2 virus and has been associated with severe inflammation leading to organ dysfunction and mortality. Our aim was to profile the transcriptome in leukocytes from critically ill patients positive for COVID19 compared to those negative for COVID19 to better understand the COVID19-associated host response. For these studies, all patients admitted to our tertiary care intensive care unit (ICU) suspected of being infected with SARS-CoV-2, using standardized hospital screening methodologies, had blood samples collected at the time of admission to the ICU. Transcriptome profiling of leukocytes via ribonucleic acid sequencing (RNAseq) was then performed and differentially expressed genes as well as significantly enriched gene sets were identified.

**Results:**

We enrolled seven COVID19 + (PCR positive, 2 SARS-CoV-2 genes) and seven age- and sex-matched COVID19- (PCR negative) control ICU patients. Cohorts were well-balanced with the exception that COVID19− patients had significantly higher total white blood cell counts and circulating neutrophils and COVID19 + patients were more likely to suffer bilateral pneumonia. The mortality rate for this cohort of COVID19 + ICU patients was 29%. As indicated by both single-gene based and gene set (GSEA) approaches, the major disease-specific transcriptional responses of leukocytes in critically ill COVID19 + ICU patients were: (i) a robust overrepresentation of interferon-related gene expression; (ii) a marked decrease in the transcriptional level of genes contributing to general protein synthesis and bioenergy metabolism; and (iii) the dysregulated expression of genes associated with coagulation, platelet function, complement activation, and tumour necrosis factor/interleukin 6 signalling.

**Conclusions:**

Our findings demonstrate that critically ill COVID19 + patients on day 1 of admission to the ICU display a unique leukocyte transcriptional profile that distinguishes them from COVID19− patients, providing guidance for future targeted studies exploring novel prognostic and therapeutic aspects of COVID19.

## Introduction

Coronavirus disease (COVID) 19 is continuing to spread rapidly throughout the world, negatively impacting infected individuals, the health systems that support them and the global economy. The novel coronavirus that causes COVID19, SARS-CoV-2, was zoonotically derived from the Wuhan region, in Hubei Province, China in late 2019 [1, 2]. Patients infected with SARS-CoV-2 exhibit a range of symptoms from mild hypoxemia with preserved lung compliance, and a mild inflammatory response, to severe hypoxemia associated with loss of lung function and a dysregulated inflammatory response with sustained tumour necrosis factor (TNF) and serine proteases that typically necessitates admission to an intensive care unit (ICU) [3–6]. The overall mortality rate of individuals with COVID19 has been reported to be approximately 3.4%; however, once COVID19 patients are admitted to the ICU, their mortality rate approximates 31–40% with a median of 9 days to ICU death [5–8].

Early reports have suggested that dysregulated cytokine response drives the severity of organ injury and dysfunction in COVID19 patients who require life support [5, 9, 10]. Transcriptional profiling of whole blood from these patients indicate an early and dynamic inflammatory response with enhanced expression of interleukin 1β (IL1β) associated genes and T cell activation [11]. However, these observations were made in comparison to healthy controls, and the increased circulating levels of pro-inflammatory cytokines observed in patients with COVID19 are also observed in other forms of sepsis and acute respiratory distress syndrome (ARDS) [4, 10]. Our aim was to transcriptionally profile COVID19-positive (COVID19 +) ICU patients in comparison to COVID19-negative (COVID19-) ICU patients with severe acute respiratory diseases/conditions [5–7].

## Methods

### Study participants and clinical data

This study was approved by the Human Subject Research Ethics Board at Western University [5–7]. Patients who were admitted to our academic ICU and suspected of having COVID19 based on standard hospital screening procedures and who had acute non-cardiogenic hypoxic respiratory failure requiring mechanical ventilation > 48 h were consecutively enrolled in the study. Patients were then separated into cohorts, either COVID19 + or COVID19−, based on detection of two SARS-CoV-2 viral genes using polymerase chain reaction (PCR). Patient baseline characteristics were recorded on admission and included age, sex, severity of illness scores, comorbidities, hematologic labs, creatinine, arterial partial pressure-to-inspired oxygen (*P*/*F*) ratio, chest X-ray findings and sepsis diagnosis using Sepsis 3.0 criteria [12]. Clinical interventions received in the ICU included use of antibiotics, anti-viral agents, systemic corticosteroids, vasoactive medications, renal replacement therapy, high-flow oxygen therapy, and mechanical ventilation (invasive and non-invasive). Final participant groups were constructed by identifying 7 COVID19 + patients and then matching to 7 COVID19− patients by age and sex only [5–7]. These patients have been included in previous studies performed by our group and this is a retrospective evaluation of the biological data from these patients [5–7]. Further, given the exploratory nature of the study, no sample size calculation was performed.

### Blood draws

Blood was collected from patients upon ICU admission using standard operating procedures to ensure all samples were treated rapidly and equally [5–7]. Blood was obtained via indwelling catheters and placed immediately on ice. Once transferred to a negative pressure hood, the blood was centrifuged and the buffy coat was isolated and frozen at − 80 °C. All samples were stored frozen in the Translational Research Centre, London, Ontario (directed by Dr. D.D. Fraser; https://translationalresearchcentre.com/) until use and freeze/thaw cycles were avoided [13, 14].

### RNA isolation

Buffy coat cells were homogenized in Trizol LS and centrifuged at 12,000 rcf to remove cell debris. Following chloroform extraction, RNA was isolated using the RNeasy Micro Plus Kit (Qiagen) according to the manufacturer’s protocol.

### Illumina NextSeq next generation sequencing

All samples were sequenced at the London Regional Genomics Centre (Robarts Research Institute, London, Ontario, Canada; http://www.lrgc.ca) using the Illumina NextSeq 500 (Illumina Inc., San Diego, CA).

Total RNA samples were quantified using the NanoDrop (Thermo Fisher Scientific, Waltham, MA) and quality was assessed using the Agilent 2100 Bioanalyzer (Agilent Technologies Inc., Palo Alto, CA) and the RNA 6000 Nano kit (Caliper Life Sciences, Mountain View, CA). Only samples with an RNA integrity number ≥ 6.0 were used. The samples were then processed using the Vazyme VAHTS Total RNA-seq (H/M/R) Library Prep Kit for Illumina (Vazyme, Nanjing, China), which includes ribosomal RNA (rRNA) reduction.

Briefly, samples were rRNA depleted, fragmented, and utilized for cDNA synthesis and PCR amplification with indexed primers to permit equimolar pooling of samples into one library. The pooled library size distribution was assessed on an Agilent High Sensitivity DNA Bioanalyzer chip, and quantitated using the Qubit 2.0 Fluorometer (Thermo Fisher Scientific, Waltham, MA).

**Fig. 1:**
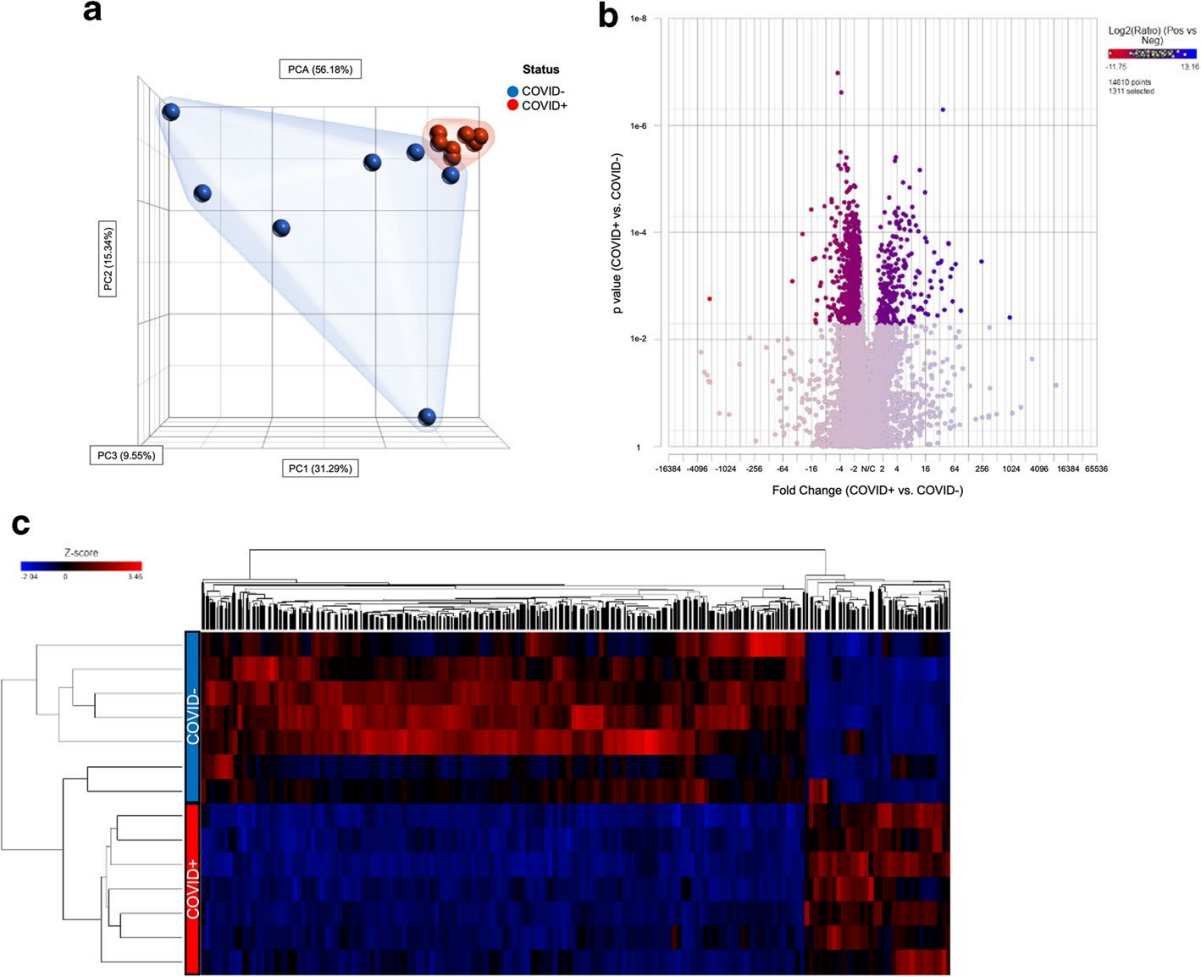
Leukocytes from COVID19 + ICU patients have a unique transcriptional profile compared to leukocytes from COVID19- ICU patients. **a** Principal component analysis (PCA) plot of total leucocyte (buffy coat) RNA samples derived from COVID19-positive (COVID +) and negative (COVID−) patients after removing the batch effect of date and interaction between date and status, using all principal components and features contributing equally. **b** Volcano plot of 1311 genes (highlighted) differentially expressed between leucocyte RNA samples derived from COVID19 positive (COVID +) versus negative (COVID−) samples based on a filtering criterion of ± 1.5-Fold change and an FDR step-up *p*-value cut-off ≤ 0.0545. **c** Heat map of 1311 genes that were differentially expressed between leucocyte RNA samples derived from COVID19 positive (COVID +) versus negative (COVID−) samples using average linkage, Euclidean distance metric and standardize normalization mode (shift mean to 0 and scale standard deviation to 1 on all features)

The library was sequenced on the Illumina NextSeq 500 as single end runs, 1 × 76 bp, using High Output v2 kits (75 cycles). Fastq data files were analysed using Partek Flow (St. Louis, MO; Additional file 1: Figure S1). After importation, the data were aligned to the *Homo sapiens* genome hg19 using STAR 2.7.3a and annotated using RefSeq Transcripts 93. Features with more than 18 reads were normalized using Trimmed Mean of M-values (TMM) (https://doi.org/10.1186/gb-2010-11-3-r25) followed by adding 0.0001. Any batch effect due to the run date was removed using Partek Flow’s Remove batch effect tool based on the results of the principal component analysis (PCA) plot and a dissimilarity plot analysis (Fig. 1a and Additional file 2: Figure S2). The gene-specific analysis (GSA) function of Partek Flow was then used to determine differential gene expression in COVID19 + vs. COVID19− patients using Akaike Information Criteria corrected (AICc)—a repeated measure analysis using mixed models methodology. The filtered gene list (fold change of 1.5 and FDR *p-*value ≤  0.0545) was then submitted to Metascape (https://metascape.org/gp/index.html#/main/step1) using express analysis of *H. sapiens* gene IDs [15]. A subset of enriched terms were selected and rendered as a network plot, where terms with a similarity > 0.3 were connected by edges. Terms with the lowest *p*-values from each of the 20 clusters were selected, with the constraint that there are no more than 15 terms per cluster and no more than 250 terms in total. The network was visualized using Metascape, where each node represented an enriched term and was coloured first by cluster ID and then by *p*-value. The filtered gene list was also analysed for enriched KEGG pathway terms.

### Gene set enrichment analysis (GSEA)

Functional enrichment of the cumulative changes in gene expression across a priori defined gene sets was performed in GSEA to avoid the limitations of single-gene differential gene expression methods [16]. All 14,810 genes that passed quality control were included in this analysis. The MSigDB collection was used to perform three main enrichment analyses: (i) Hallmark gene set (50 gene sets containing overlaps between MSigDB collections that display coordinate expression); (ii) Canonical Pathways (C2, 2232 gene sets); and (iii) regulatory target gene sets (C3, transcription factors, 1137 gene sets) [17]. The gene sets included in the analysis were limited to those that contained between 10 and 500 genes. Permutation was conducted 1000 times according to default-weighted enrichment statistics and by using a signal-to-noise metric to rank genes according to their differential expression levels across the COVID19 + vs. COVID19− groups. Significant gene sets were defined as those with an FDR ≤ 0.1. Visualization and network analyses were performed using the EnrichmentMap application for Cytoscape 3.8.0 (https://cytoscape.org/) according to default parameters [18]. To determine the degree of similarity between gene sets (nodes), the combined coefficient using a merged version of the Jaccard and overlap similarity coefficients was used to define edges (i.e. connecting lines) between the nodes (cut-off 0.375).

### Population statistics

Medians (IQRs) and frequency (%) were utilized to describe patient baseline characteristics for continuous and categorical variables, respectively. Continuous variables were compared using two-tailed Mann–Whitney U or Kruskal–Wallis tests, as appropriate. Categorical variables were compared using Fisher's exact test. SPSS version 26 (IBM Corp., Armonk, NY, USA) was used to perform all population statistics and *p*-values < 0.05 were considered statistically significant.

## Results

We profiled the leukocyte transcriptome of 7 COVID19 + ICU patients (median years of age = 60.0, IQR = 56.0, 67.0) and 7 age- and sex-matched COVID19− ICU patients (median years of age = 60.0, IQR = 53.0, 63.0; *p* = 0.520) on day 1 of admission to the ICU. Baseline demographic characteristics, comorbidities, laboratory values, and chest X-ray findings are reported in Table 1. Compared to the COVID19 + , COVID19− ICU patients were more likely to have an increased white blood cell count, specifically neutrophils; however, COVID19 + ICU patients were more likely to have bilateral pneumonias. While SARS-CoV-2 was confirmed by PCR in 100% of COVID19 + ICU patients, an infectious agent was only identified in 43% of COVID19− ICU patients and was 'suspected' in the remaining 57%. The mortality rate was 29% for COVID19 + ICU patients.

**Table 1:** Subject demographics and clinical data

Variable	COVID19 + patients	COVID19– patients	*p*-value
n	7	7	1.000
Age in years	60.0 (56.0, 67.0)	60.0 (53.0, 63.0)	0.520
Sex	5F:2 M	5F:2 M	1.000
MODS	4.0 (1.0, 7.0)	5.0 (3.0, 7.0)	0.400
SOFA	4.0 (3.0, 9.0)	5.0 (4.0, 11.0)	0.334
Comorbidities, n (%)			
Hypertension	4 (57.1)	6 (85.7)	0.559
Diabetes	2 (28.6)	3 (42.9)	1.000
Chronic kidney disease	1 (14.3)	0 (0)	1.000
Cancer	1 (14.3)	1 (14.3)	1.000
Admission laboratory values			
WBC (× 10^9^/L)	8.2 (3.8, 12.9)	19.3 (13.6, 24.8)	0.018*
Neutrophils (× 10^9^/L)	7.3 (3.8, 11.1)	13.0 (11.7, 22.2)	0.025*
Lymphocytes (× 10^9^/L)	0.7 (0.6, 1.0)	1.4 (0.4, 1.7)	0.608
Platelets (× 10^9^/L)	202 (119, 225)	212 (145, 291)	0.565
Haemoglobin (g/L)	122 (104, 137)	123 (98, 137)	0.898
Creatinine (µmol/L)	68 (45, 184)	65 (49, 80)	0.609
*p*:F ratio	124 (69, 202)	172 (132, 304)	0.317
Admission chest X-ray findings, n (%)			
Bilateral pneumonia	7 (100)	1 (14.3)	0.005*
Unilateral pneumonia	0 (0)	4 (57.1)	0.070
Interstitial infiltrates	0 (0)	1 (14.3)	1.000
Normal	0 (0)	1 (14.3)	1.000
Sepsis diagnosis			
Suspected	0 (0)	4 (57.1)	0.070
Confirmed	7 (100)	3 (42.9)	0.070
Interventions during study			
Antibiotics	7 (100)	7 (100)	1.000
Anti-virals	3 (42.9)	0 (0)	0.192
Steroids	1 (14.3)	2 (28.6)	1.000
Vasoactive medications	5 (71.4)	5 (71.4)	1.000
Renal replacement therapy	1 (14.3)	0 (0)	1.000
High-flow nasal cannula	3 (42.9)	1 (14.3)	0.559
Non-invasive mechanical ventilation	4 (57.1)	7 (100)	0.192
Invasive mechanical ventilation	5 (71.4)	6 (85.7)	1.000
ICU outcome			
Survived	5 (71.4)	7 (100)	0.462

Continuous data are presented as medians (IQRs). *MODS* Multiple Organ Dysfunction Score, *SOFA* Sequential Organ Failure Assessment Score, *COPD* Chronic Obstructive Pulmonary Disease, *WBC* white blood cell

Principal components analysis revealed clustering of samples based on SARS-CoV-2 PCR status, suggesting that changes in gene expression in circulating leukocytes in COVID19 + patients were unique to SARS-CoV-2, rather than common to sepsis or/and ARDS in critically ill COVID19− patients (Fig. 1a). GSA yielded 1,311 differentially expressed genes (false discovery rate < 0.055 and a > 1.5-fold increase or decrease in expression; Additional file 3: Table S1), 254 of which were upregulated and 1057 of which were downregulated (Fig. 1b, c) in the circulating immune cells of COVID19 + patients.

**Fig. 2:**
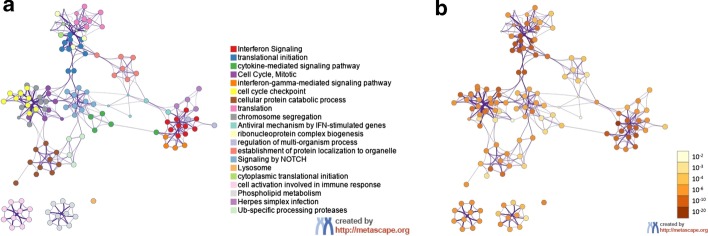
Metascape functional analysis of transcriptional differences in circulating leukocytes from COVID19 + and COVID19− critically ill patients. The list of 1311 differentially expressed genes was submitted to Metascape using express analysis of *Homo sapiens* gene IDs. A subset of enriched terms was selected and rendered as a network plot, where terms with a similarity > 0.3 were connected by edges. The network was visualized using Metascape. Each node represents an enriched term and coloured first by its cluster ID (**a**) and then by its *p*-value (**b**)

**Fig. 3:**
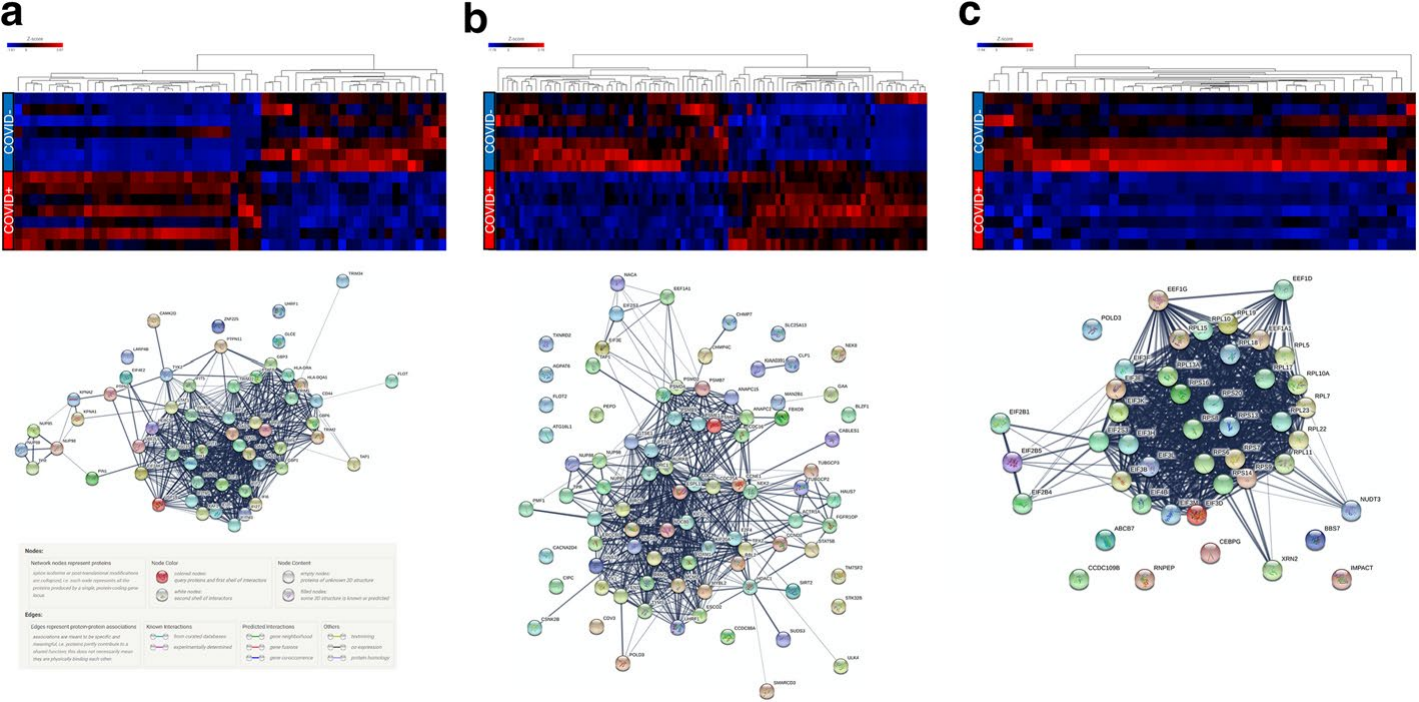
Gene expression and predicted protein–protein interaction networks of transcriptional differences in circulating leukocytes from COVID19 + and COVID19− critically ill patients. Differentially expressed genes (1311) were submitted to the Reactome Pathway Browser, resulting in the identification of three major pathway classifications (shown as heat maps, left to right): **a** interferon (interferon signalling, interferon alpha/beta signalling, anti-viral mechanism by IFN-stimulated genes); **b** cell cycle regulation (cell cycle, mitotic, G1/S transition, mitotic G1 phase and G1/S transition, G1/S-specific transcription); and **c** protein translation/ribosomes (GTP hydrolysis and joining of the 60S ribosomal subunit, formation of a pool of free 40S subunits, formation of the ternary complex, the 43S complex, eukaryotic translation initiation, Cap-dependent translation initiation, L13a-mediated translational silencing of ceruloplasmin expression, eukaryotic translation elongation, peptide chain elongation, translation initiation complex formation, response of EIF2AK4 (GCN2) to amino acid deficiency). The gene lists corresponding to these three pathway classifications were submitted to String-db (https://string-db.org/cgi/input.pl) to predict the protein–protein interaction networks (shown below each corresponding heat map)

Functional enrichment analysis performed in Enrichr (https://maayanlab.cloud/Enrichr/) suggested an overrepresentation of genes that characterize CD14 + monocytes and CD33 + myeloid cells (Additional file 4: Table S2) in COVID19 + patients. Further functional analysis using Metascape (Fig. 2) revealed an overall upregulation in IFN mediated gene transcription in leukocytes of COVID19 + ICU patients, whereas genes associated with protein translation were all downregulated in the leukocytes of COVID19 + relative to COVID19− ICU patients (Fig. 3). These findings were independently confirmed using an alternative analysis tool, Reactome (Additional file 5: Figure S3), indicating highly reproducible responses.

**Fig. 4:**
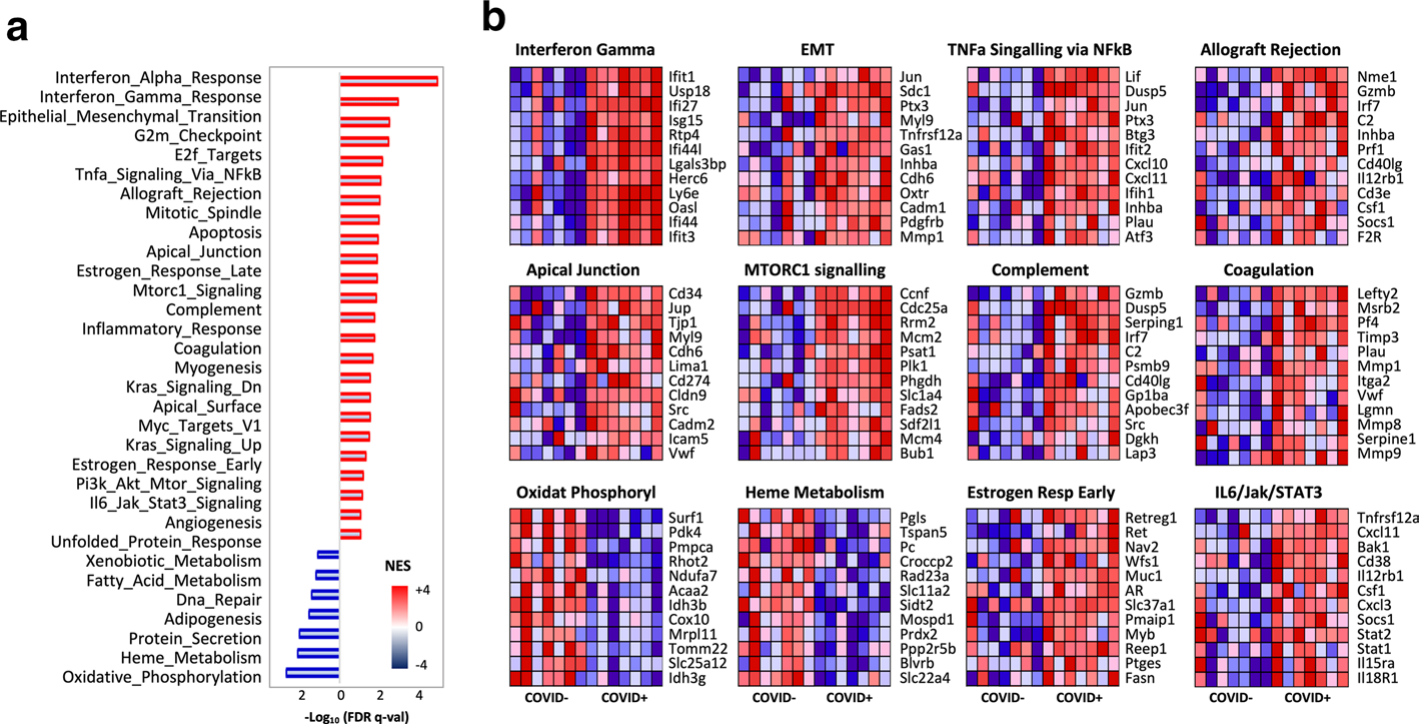
Gene set enrichment analysis of transcriptional differences in circulating leukocytes from COVID19 + and COVID19− critically ill patients. **a** Histogram of Hallmark gene set enrichment analysis. A total of 26/50 gene sets were positively enriched in the phenotype COVID positive ( +) and 7/50 gene sets showed positive enrichment for the COVID negative (−) phenotype. Differentially regulated gene sets were ranked by normalized enrichment score (NES) and plotted against the –log_10_ of the false discovery rate (FDR *q* value). **b** Heat maps of the top 12 gene sets in the ranking list in **a**. Red indicates positive enrichment and blue indicates negative enrichment of gene transcripts.

GSEA was then used to identify differentially expressed gene sets in the entire data set. After correcting for multiple comparisons, GSEA revealed that about 68.5% of genes were decreased compared to 31.5% that were increased in COVID19+ compared to COVID19− ICU patients. Furthermore, a total of 7/50 gene sets were negatively enriched while 26/50 were positively enriched (FDR < 10%). In addition to overrepresentation of genes associated with IFN signalling (Fig. 4), GSEA identified enrichment for gene sets involved in TNF and IL6 signalling, complement signalling, apoptosis, and coagulation pathways in COVID19 + ICU patients (Fig. 4). In contrast, gene transcripts contributing to metabolic pathways involving protein synthesis, oxidative phosphorylation, and DNA repair were consistently and markedly downregulated in COVID19 + ICU patients (Fig. 4). GSEA was also used to identify enrichment of specific transcription factor (TF) binding sites in the leukocytes of COVID19 + relative to COVID19− ICU patients that may shed light on the observed disease-associated modifications of leukocyte transcriptomes. Network analysis confirmed an overrepresentation of genes that shared cis-regulatory sequences for serum response factor (SRF), E2F/1, nuclear factor kappa beta (NFκB), and cAMP response element-binding protein (CREB) in the COVID19 + data set (Additional file 6: Figure S4).

**Fig. 5:**
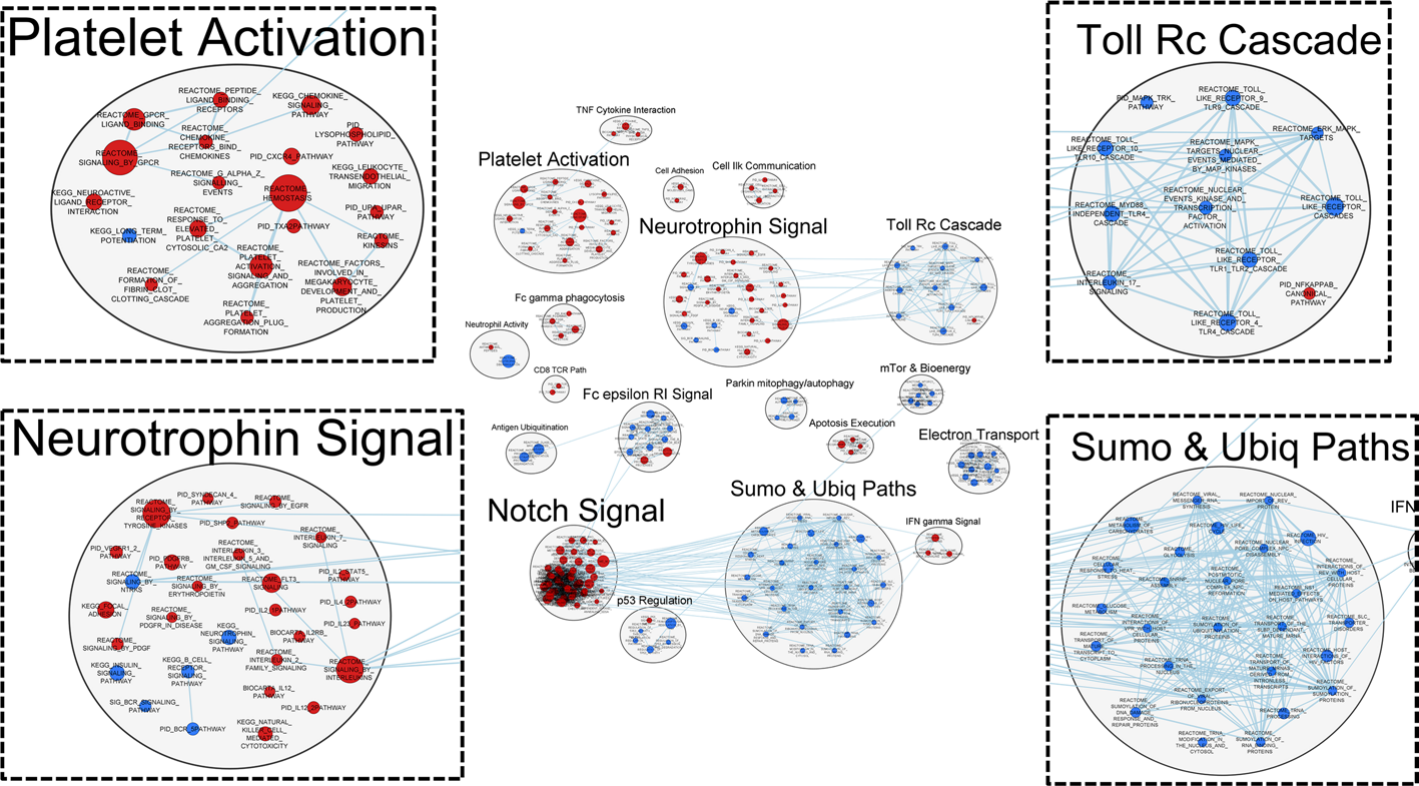
Correlation network generated from transcriptional differences in circulating leukocytes derived from COVID19 + and COVID19− critically ill patients. Each node represents a unique gene set and the edges represent the coefficient of similarity between gene sets above a defined threshold. Markov Cluster Algorithm (MCL) clustering analyses revealed groups of gene transcript nodes, where platelet activation, neurotrophin signalling, toll-like receptor signalling cascades, and sumoylation/ubiquitination pathways were identified as the top four nodes. Red indicates positive enrichment of gene transcripts and blue indicates negative enrichment of gene transcripts.

Enrichment Map and the Autoannotate function in Cytoscape 3.8.0 was used to perform network analyses to visualize higher level changes in biological organization. This approach identifies tightly connected nodes, or clusters, indicating shared relationships between differentially expressed genes (shared gene membership). We used an FDR cut-off of ≤ 0.1 and a nominal *p*-value of ≤ 0.01 to select genes sets for network analysis. Figure 5 highlights the top network clusters and key biological processes that were differentially regulated in COVID19 + vs. COVID19− ICU patients. Notable clusters/processes identified as being either positively or negatively enriched in COVID19 + ICU patients by these analyses included platelet activation (20 gene sets), neurotrophin signalling (29 gene sets), SUMOylation and ubiquitination of proteins (27 gene sets), and toll receptor cascades (12 gene sets).

## Discussion

In this study, we compared leukocyte transcriptional profiling of COVID19 + to COVID19− ICU patients. The study was designed in such a way that control COVID19− ICU patients were grossly clinically indistinguishable from the COVID19 + patients. Despite similar clinical features, the most striking result was that patients were distinguishable based on leukocyte transcriptional profile (e.g. unique molecular features could be picked up in those that were positive for the SARS-Cov2 virus vs. those that were not). One difference that was evident between COVID19 + and COVID19− ICU patients was the identification of an infectious agent. However, while no agent was identified in 57% of the COVID19− ICU patients, the presence of an infectious agent was suspected. Moreover, this is not uncommon in sepsis as the literature suggests that in at least 30% of sepsis cases, a pathogen cannot be detected [19].

The consistent differences between the transcriptional profiles of COVID19 + patients and COVID19− patients with non-COVID lung injury that we observed are similar to previous clinical reports indicating that COVID19 + patients do not exhibit “typical” symptoms of acute respiratory distress [3], and that COVID19 + patients have a unique inflammatory profile [5, 6]. As indicated by both single-gene based and gene set (GSEA) approaches, the major disease-specific transcriptional responses of leukocytes in critically ill COVID19 + ICU patients were: (i) a robust overrepresentation of IFN related gene expression; (ii) a marked decrease in the transcriptional level of genes contributing to protein synthesis and bioenergy metabolism; and (iii) the dysregulated expression of genes associated with coagulation, platelet function, complement activation, and TNF/IL6 signalling.

Functional enrichment analysis also highlighted a marked shift in cellular population within the circulation with a significant enrichment for transcriptional profiles associated with CD14 + monocytes and CD33 + myeloid cells, underscoring the lymphopenia observed in COVID19 + ICU patients. Further, compared to COVID19− patients, COVID19 + patients exhibited an overall decrease in the expression of genes associated with these cell types, suggesting a potential shift in the innate immune response. Moreover, these data can be interpreted to indicate a decrease in CD14 + monocyte activation and/or a decrease in overall CD14 + monocyte number within COVID19 + ICU patients compared to COVID19− ICU patients, who exhibited increased white blood cells.

Disease-specific alterations in IFN signalling have been previously identified in patients with COVID19 [20], and our recent publication on a similar patient cohort identified significantly increased levels of IFNs in the plasma of COVID19 + ICU patients compared to both age- and sex-matched COVID19− ICU patients and healthy controls [5]. Classical type I (*α*/*β*) and type II (*γ*) IFN signalling through IFN receptors and Janus kinase-signal transducer and activator of transcription (JAK-STAT) pathways activate IFN-stimulated genes (ISGs), in part through nuclear factor kappa B (NFkB) signalling, that mediate a complex web of responses that include, but are not limited to, pathogen sensing, sensitization, desensitization and inhibition of viral entry [21–23]. Toll-like receptor (TLR)-mediated cascades, independently identified in our network correlation analyses, are one of the major pathogen sensing pathways activated by type I IFN signalling. In parallel with the induction of ISG transcription, IFN signalling promotes a global shutdown in translation and cellular RNA degradation to induce the death of virus-infected cells. This correlates with the upregulation of E2F genes that regulate the core transcriptional machinery driving cell cycle progression, dictating the timing and fidelity of genome replication and ensuring genetic material is accurately passed on through each cell division cycle [23].

While transient activation of IFN signalling pathways often occurs during viral infections, sustained IFN signalling can paradoxically promote viral persistence and induce both immune suppression and chronic inflammation [24]. For example, chronically increased IFN levels have been implicated in the inhibition of T cell proliferation and chemokine release, and the TRAIL-induced apoptosis of CD4 + T cells during both infectious and non-infections pathologies [25, 26]. While TRAIL (*TNFSF10*) expression was not identified as significantly increased in our patient population, there was a trend towards enhanced *TNFSF10* expression in COVID19 + compared to COVID19- patients at day 1 of ICU admission. In addition to TRAIL, sustained type I IFN levels can also induce the expression of pro-apoptotic molecules while enhancing the immunosuppressive actions of dendritic cells, monocytes, and macrophages through augmented expression of cytokines [24]. Thus, sustained IFN signalling can effectively “derail” the normal innate immune response and promote viral persistence at both the cellular and tissue level.

Potentially relevant to COVID19 + ICU patients, chronically high levels of IFNγ have also been detected in, and postulated to be causal of, macrophage activation syndrome (MAS). Moreover, dysregulated and persistent macrophage activation has been previously described in COVID19 + patients [27]. In this scenario, SARS-CoV-2 infection would lead to defects in lymphocyte cytolytic activity [28], where natural killer (NK) cells and cytolytic CD8 + T cells exhibit reduced capacity to lyse infected and otherwise activated antigen presenting cells, resulting in prolonged cell–cell interactions with persistent amplification of pro-inflammatory cytokine cascades [29]. The subsequent cytokine response induces macrophage activation and haemophagocytic lymphohistiocytosis, ultimately contributing to multi-organ dysfunction and death [30]. While this is a compelling working hypothesis, larger scale and more focussed analyses will be required to test this possibility.

Transcriptomic signatures are expected to differ depending on the specific cell populations being examined, which can account for variation in overall trends that can be identified. For example, a recent study by Jeannet and colleagues [31] focused on lymphocytes and suggested that these cells are immunosuppressed and exhausted. However, as our understanding of the immunopathology of COVID19 evolves, it has been suggested that uncontrolled macrophage and monocyte activation due to a dysfunctional interferon response to SARS-CoV-2 infection plays a key role in subsequent inflammatory response and organ injury [32–34]. It is likely that this is the predominant signature in our patients. Moreover, our data supports that dysregulation of macrophage/monocyte responses, sustains and enhances activation of interferon response genes in monocytes and macrophages driving immunosuppression and dysfunction in lymphocytes [27, 29, 35, 36].

In addition to IFN, our data also identified a significant enrichment in genes associated with TNF signalling in COVID19 + compared to COVID19- patients. TNF signalling is a critical pathway in the regulation of the pro-inflammatory response following infection, where it typically is observed in the very early stages of infection and returns to basal levels as the infection progresses [37, 38]. With respect to COVID19, previous work by our group in a similar patient population identified a persistent elevation of circulating TNF in COVID19 + patients [5]. Together, these studies suggest that augmented TNF signalling may be key in COVID19 and may provide a potential therapeutic target through the use of neutralizing antibodies or small molecule inhibitors as has been previously suggested [5, 39].

One of the key clinical features of COVID19 is upregulation of the pro-thrombotic phenotype and microvascular complications, leading to COVID19-associated coagulopathy [7]. This is characterized by venous, arterial, and microvascular thrombosis despite the use of anti-coagulant therapies [40–46]. Our study provides compelling evidence of COVID19-associated changes in coagulation-related gene expression levels that may exacerbate thrombosis caused by endothelial cell injury and platelet activation in COVID19 + ICU patients [7]. In particular, disease-specific upregulation of *SERPINE1* (encoding plasminogen activator inhibitor-1; PAI-1), *VWF* (von Willebrand factor), and *GZMB* (Granzyme B) gene expression levels in COVID19 + ICU patients may contribute to thrombotic disorders in COVID19 patients [5, 47]. Granzyme B is a serine protease that may be involved in thrombus formation through induction of endothelial damage and endothelial cell apoptosis [48], which we recently found to be the most discriminatory inflammatory analyte for identifying COVID19 status in ICU patients [5]. Assessed in combination, the COVID19-associated changes in the expression of genes involved in platelet activation, haemostasis, fibrin clot formation, platelet cytosolic calcium levels, and Thromboxane A2 expression, in parallel with transcriptional changes in genes regulating chemokine levels and granulocyte activation, identified in this study may provide both a unique transcriptional profile that identifies and reflects the pathophysiological mechanism(s) involved, and potential therapeutic targets for the treatment of patients with life-threatening COVID19 infections.

We have also identified a decrease in the expression of genes required for heme metabolism in the immune cells from COVID19 + ICU patients. These findings may be relevant to viral sequestration of iron from haemoglobin. Many viruses require iron for replication, and utilize a variety of mechanisms to decrease cellular iron metabolism to increase iron availability. Cell free iron can bind to damage recognition receptors, and in addition to driving oxidative stress, cell-free haemoglobin has been shown to be injurious in the lungs of patients with ARDS [49].

As shown in Fig. 5, COVID19 + ICU patients also exhibited changes in the expression of genes regulating the addition of ubiquitin moieties and/or small ubiquitin-like moieties (SUMO) to proteins. Viruses rely heavily on the host’s cellular replication machinery, including the ability to by-pass and/or exploit cellular ubiquitin and SUMOylation conjugating systems, to successfully proliferate and achieve infection. Many viruses are able to target essentially every step of ubiquitination and SUMOylation processes, including the activation of genes that encode ubiquitin ligases or other molecules that alter the intracellular pools of free ubiquitin and SUMOs available for conjugation to proteins that modulate replication processes [50].

Another intriguing network correlation identified was neurotrophin signalling. Neurotrophins are structurally related neuropeptides and include nerve growth factor (NGF) and brain-derived neurotrophic factor (BDNF). These peptides play key roles in the survival and development of peripheral and central nervous systems. In the context of COVID19, neurotrophins are likely to be components of a neuroimmune response to infection, as shown previously in the early stages of the H1N1 influenza pandemic [51]. Neurotrophins have been previously implicated in the pathogenesis of other lung conditions associated with airway inflammation and hyperreactivity, such as asthma [52]. Moreover, it is plausible that the dysregulation of neurotrophin-encoding genes in COVID19 + ICU patients may be linked to neural damage resulting from SARS-CoV-2 infection of the brainstem and/or other central nervous system (CNS) sites [53].

Collectively, the data from our exploratory study identify a unique leukocyte transcriptional profile in COVID19 + ICU patients vs. COVID19− ICU patients. However, it is important to note that, while novel, these findings are derived from a study with some design limitations. First, all the data in this study were derived from critically ill patients at a single admission time point. As such, we were unable to assess any transcriptional changes associated with ICU admission or track these changes over the course of the patients time in the ICU. Second, the study included only 7 COVID19 + and 7 COVID19− patients. However, even with what could be considered to be a small sample size, we were still able to identify a unique transcriptional profile associated with SARS-CoV-2 infection. Importantly, these studies, which are supported by previous findings by our group [5–7], will be hypothesis-generating for future studies of disease severity and/or outcome. Third, the COVID19-specific transcriptional profiles reported here have not been independently verified. However, our findings were consistent with previous studies by ourselves and others comparing COVID19 patients with healthy controls, and with the COVID19-induced changes in plasma proteins identified in our recently published studies from a similar patient cohort [5–7, 11, 31, 47, 54]. For example, increased expression of the *GZMB* and *SDC1* genes, as well as the increased expression of multiple genes encoding IFN signalling molecules, are supported by findings of increased abundance of granzyme B, syndecan-1, and IFNs, respectively, in the plasma of COVID19 + vs. COVID19− ICU patients [5–7].

We also utilized multiple approaches to analyse the transcriptome data, including both single-gene as well as gene set enrichment analysis (i.e. GSEA). GSEA applies a threshold-free overrepresentation analysis strategy to evaluate genome wide expression profiles and to determine whether a priori defined sets of genes show statistically significant, cumulative changes in gene expression that correlate with phenotype (COVID19 + vs. COVID19−). While single-gene methods can identify individual gene effects, this approach may be undermined by the variance across individuals seen in complex disease states. GSEA is complementary to single-gene analysis in providing a framework with which to examine changes in higher levels of biological organization, such that alterations in gene expression associated with a disease can manifest at the level of biological pathways or co-regulated gene sets, rather than individual genes. Thus, while we did not independently assess the changes in expression of individual genes identified in these RNAseq analyses, the collective support provided by previous publications in combination with the use of multiple analysis techniques validates our overall findings.

## Conclusion

In summary, we report a unique transcriptome in COVID19 + ICU patients that can be distinguished from those of COVID19− patients despite the heterogeneity and timing of presentation/admission to the ICU. This unique transcriptome, which can be measured using common laboratory techniques suggesting it may be amenable to future point-of-care tests, is driven by enhanced IFN signalling, dysregulated protein synthesis, and increased platelet activation and coagulation. Finally, given the significant impact of COVID19 critical illness on society, our novel data may provide guidance in future targeted studies exploring therapeutics in parallel with validation of our findings in larger COVID19 + cohorts.

## Supplementary information

**Additional file 1: Figure S1.** Data analysis pipeline in Partek Flow. Unaligned reads (fastq files) were imported and their attributes (status, date, and patient) were added manually. Pre-alignment QA/QC provided information on the total number of reads, average length, average quality (Q) scores, % N and %GC content. Bowtie2 was used to align the reads to a database of rRNA sequences to assess the effectiveness of the ribosomal RNA (rRNA) reduction step in the assay. Using default parameters, reads were aligned to the hg19 reference index using STAR 2.7.3a. Transcript abundance was quantified from the aligned reads using the Expectation/Maximization algorithm and annotated to a transcriptome, in this case RefSeq transcripts 93–2020-02-03. 18,040 genes were identified in the dataset at this point. The output was raw gene counts for each gene. The coverage report provided information on the genome coverage and number of on-target and off-target reads. Post-alignment QA/QC assembled the results and metrics from previous QC reports, as well as providing information regarding the average number of alignments per read and the percentage of unique, non-unique and unaligned reads. Filtered features were based on the Q1 feature distribution, and features with ≤ 18 reads were excluded in a conservative approach to reduce noise. The data were normalized using Trimmed Mean of M values (TMM) and by the addition of 1.0 × 10^–4^ to avoid zero values. Data dimensionality reduction allowed for visualization in three dimensions and was performed with principal component analysis (PCA) using all components and features contributing equally (all the features were standardized to a mean of 0 and a standard deviation of 1). Based on the PCA, the batch effect of the date the two runs were performed was removed, as well as the interaction of data and status, using Partek’s batch effect remover algorithm (calculating the variation attributed to the factor(s) being removed then adjusting the original values to remove the variation). Gene-specific analysis (GSA) is a statistical modelling approach used to test for differential expression of genes or transcripts. The goal of GSA is to identify a statistical model that is the best for a specific gene transcript, and then use the best model to test for it’s differential expression. Genes with a fold change of < −1.5-fold or > 1.5-fold, and an FDR step-up *p*-values of ≤ 0.0545 were filtered for further biological analysis with Partek, as well as with various third party tools. Gene set enrichment was used to find gene sets (Gene Ontology terms and KEGG pathways) that were overrepresented in the filtered gene list, based on Fisher’s exact test. Hierarchical clustering was performed by clustering samples and features using the average linkage and Euclidean distance. Features were standardized (shift mean to 0 and scale standard deviation to 1) to normalize the data and facilitate data display in heat maps.

**Additional file 2: Figure S2.** Dissimilarity plots of COVID positive and negative samples, before (left) and after (right) the batch effect removal tool was used visualize hidden structures in the data—such as the presence of outliers or persistent batch effects after correction. Entries with lower dissimilarities (higher similarity) are plotted darker.

**Additional file 3: Table S1.** Differentially expressed genes (COVID19 + vs. COVID19-)

**Additional file 4: Table S2.** Top significant cell types from Enrichr (*p* < 0.05)

**Additional file 5: Figure S3.** Differentially expressed genes (1,311) were submitted to the Reactome Pathway Browser and the top 25 pathways from Reactome (https://reactome.org/) were identified. The number of genes in each pathway is represented by the blue bars, and the FDR *p*-value for each pathway is represented by the orange line.

**Additional file 6: Figure S4.** Transcription factor analysis data set where nodes represent unique gene sets containing genes known to share 5′ cis-regulatory sequences for the binding of specific transcription factors. The edges represent the coefficient of similarity between the gene sets above a defined threshold (Jaccard and overlap similarity coefficient cut-off 0.375). Groups of nodes were obtained by Markov Cluster Algorithm (MCL) clustering into gene modules. The highlighted nodes were associated with a given transcription factor, suggesting a co-regulatory network. Red indicates positive transcript enrichment and blue indicates negative transcript enrichment.

## Data Availability

The datasets generated and/or analysed during the current study are available in the GEO repository (Accession #: GSE154998), https://www.ncbi.nlm.nih.gov/geo/query/acc.cgi?acc=GSE154998. Datasets are also available from the corresponding author upon reasonable request.
