# Supplementary figures and images for: Transcriptional profiling of leukocytes in critically ill COVID19 patients: implications for interferon response and coagulation
Source: Intensive Care Med Exp. 2020 Dec 11;8:75. doi: 10.1186/s40635-020-00361-9 (PMC7729690; doi:10.1186/s40635-020-00361-9)

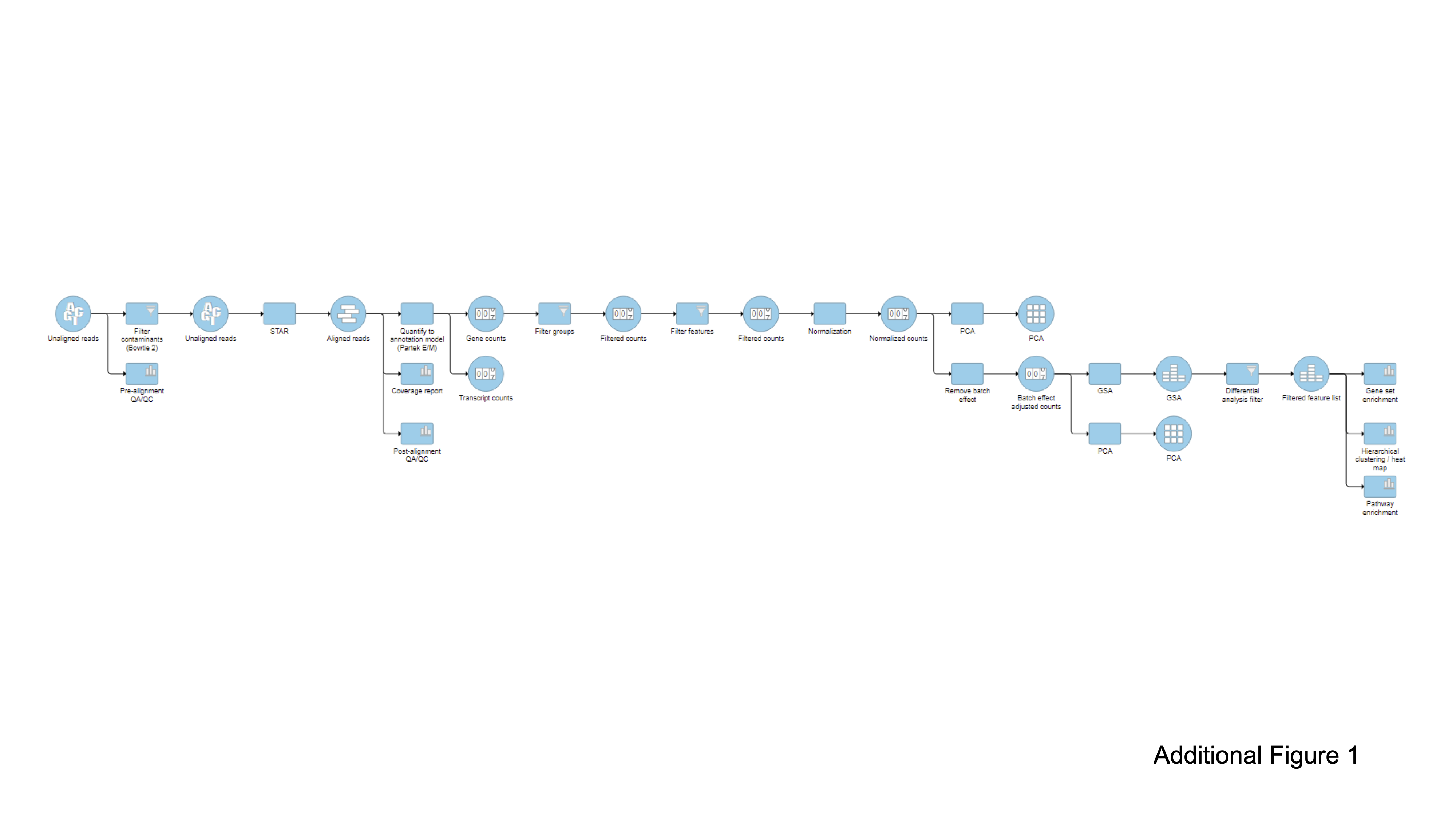

Supplement: Supplementary file 1 — Additional file 1: Figure S1. Data analysis pipeline in Partek Flow. Unaligned reads (fastq files) were imported and their attributes (status, date, and patient) were added manually. Pre-alignment QA/QC provided information on the total number of reads, average length, average quality (Q) scores, % N and %GC content. Bowtie2 was used to align the reads to a database of rRNA sequences to assess the effectiveness of the ribosomal RNA (rRNA) reduction step in the assay. Using default parameters, reads were aligned to the hg19 reference index using STAR 2.7.3a. Transcript abundance was quantified from the aligned reads using the Expectation/Maximization algorithm and annotated to a transcriptome, in this case RefSeq transcripts 93–2020-02-03. 18,040 genes were identified in the dataset at this point. The output was raw gene counts for each gene. The coverage report provided information on the genome coverage and number of on-target and off-target reads. Post-alignment QA/QC assembled the results and metrics from previous QC reports, as well as providing information regarding the average number of alignments per read and the percentage of unique, non-unique and unaligned reads. Filtered features were based on the Q1 feature distribution, and features with ≤ 18 reads were excluded in a conservative approach to reduce noise. The data were normalized using Trimmed Mean of M values (TMM) and by the addition of 1.0 × 10^–4^ to avoid zero values. Data dimensionality reduction allowed for visualization in three dimensions and was performed with principal component analysis (PCA) using all components and features contributing equally (all the features were standardized to a mean of 0 and a standard deviation of 1). Based on the PCA, the batch effect of the date the two runs were performed was removed, as well as the interaction of data and status, using Partek’s batch effect remover algorithm (calculating the variation attributed to the factor(s) being removed then adjusting the o [file 40635_2020_361_MOESM1_ESM.tiff]

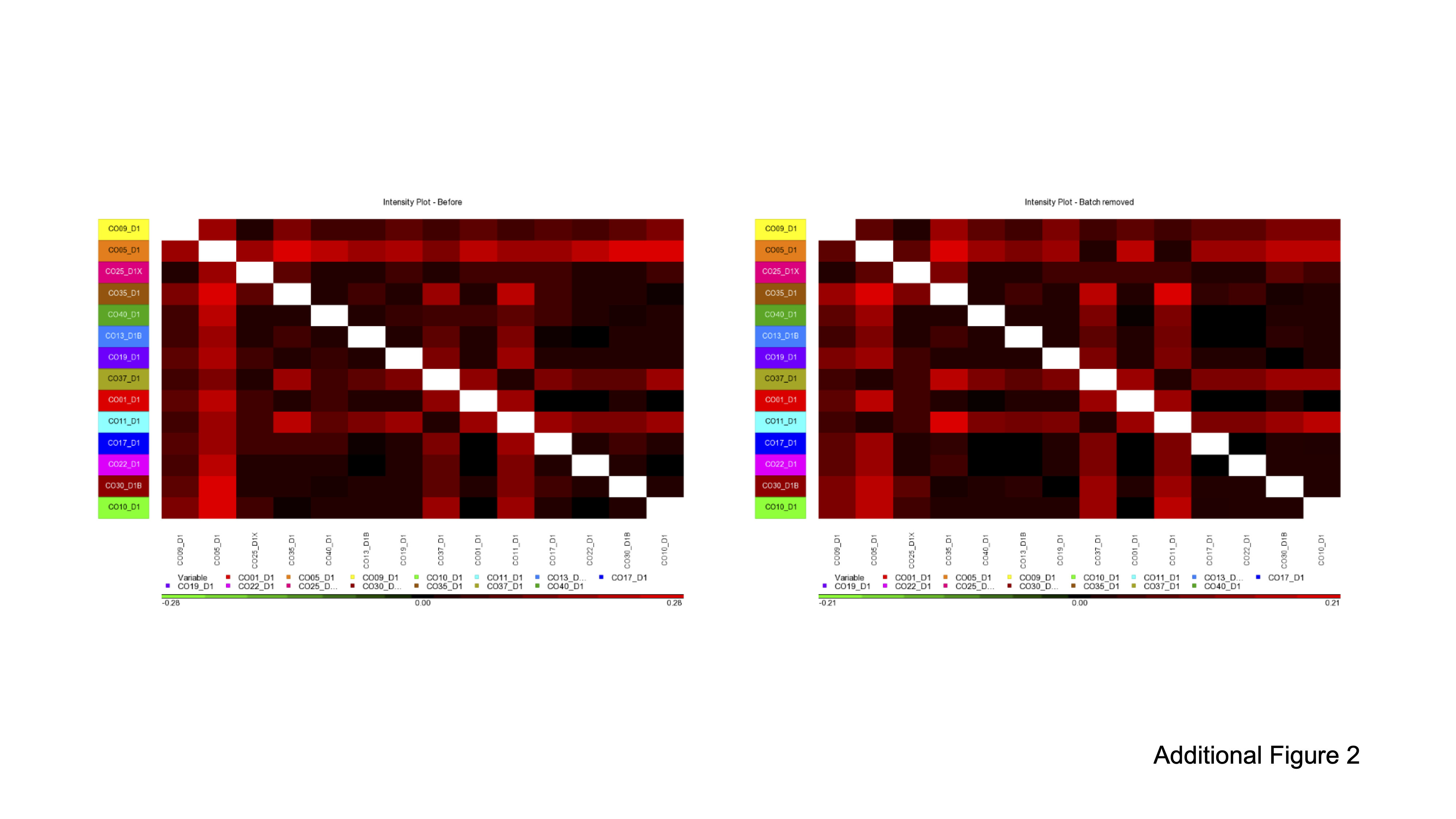

Supplement: Supplementary file 2 — Additional file 2: Figure S2. Dissimilarity plots of COVID positive and negative samples, before (left) and after (right) the batch effect removal tool was used visualize hidden structures in the data—such as the presence of outliers or persistent batch effects after correction. Entries with lower dissimilarities (higher similarity) are plotted darker. [file 40635_2020_361_MOESM2_ESM.tiff]

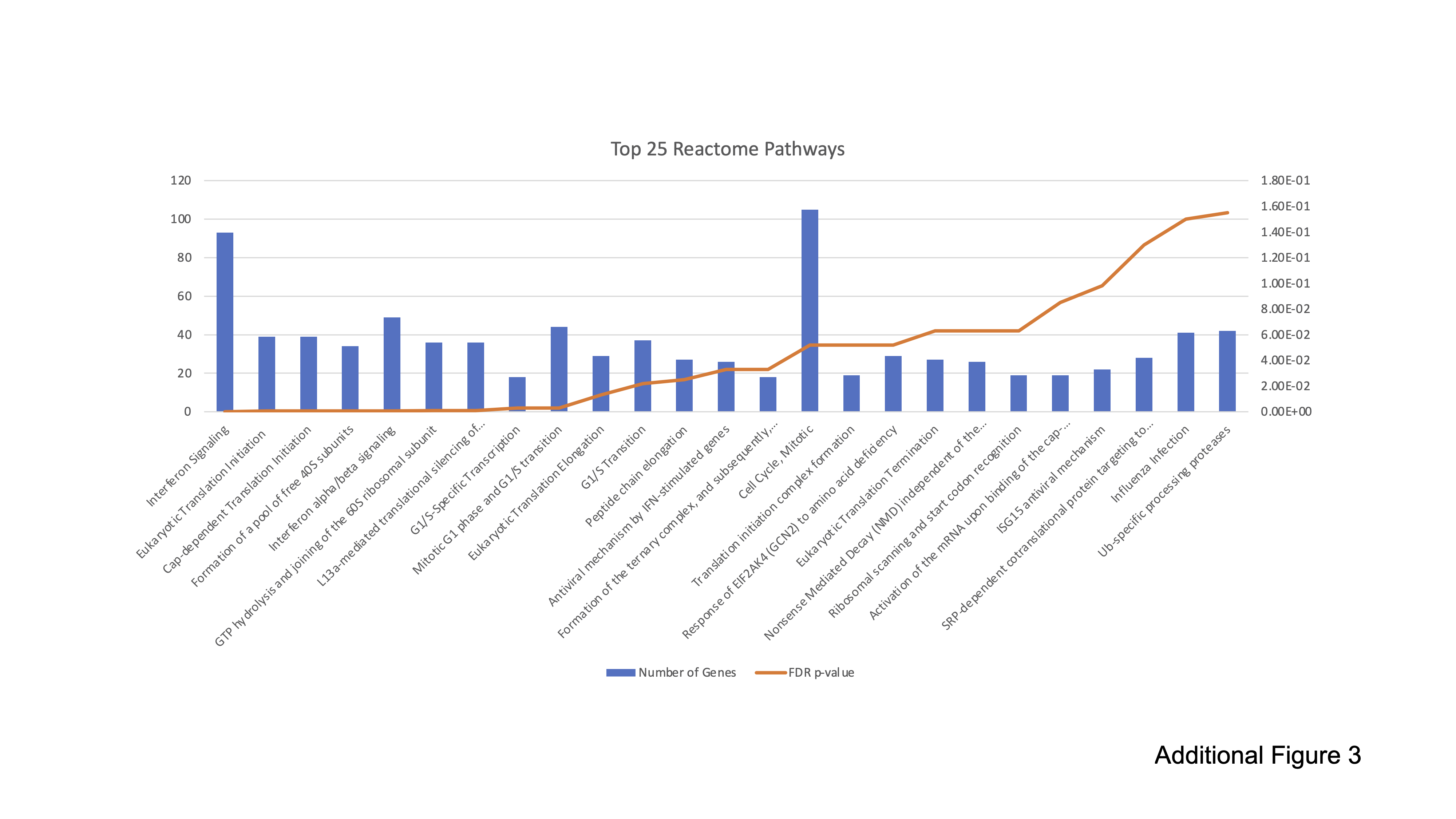

Supplement: Supplementary file 5 — Additional file 5: Figure S3. Differentially expressed genes (1,311) were submitted to the Reactome Pathway Browser and the top 25 pathways from Reactome (https://reactome.org/) were identified. The number of genes in each pathway is represented by the blue bars, and the FDR p-value for each pathway is represented by the orange line. [file 40635_2020_361_MOESM5_ESM.tiff]

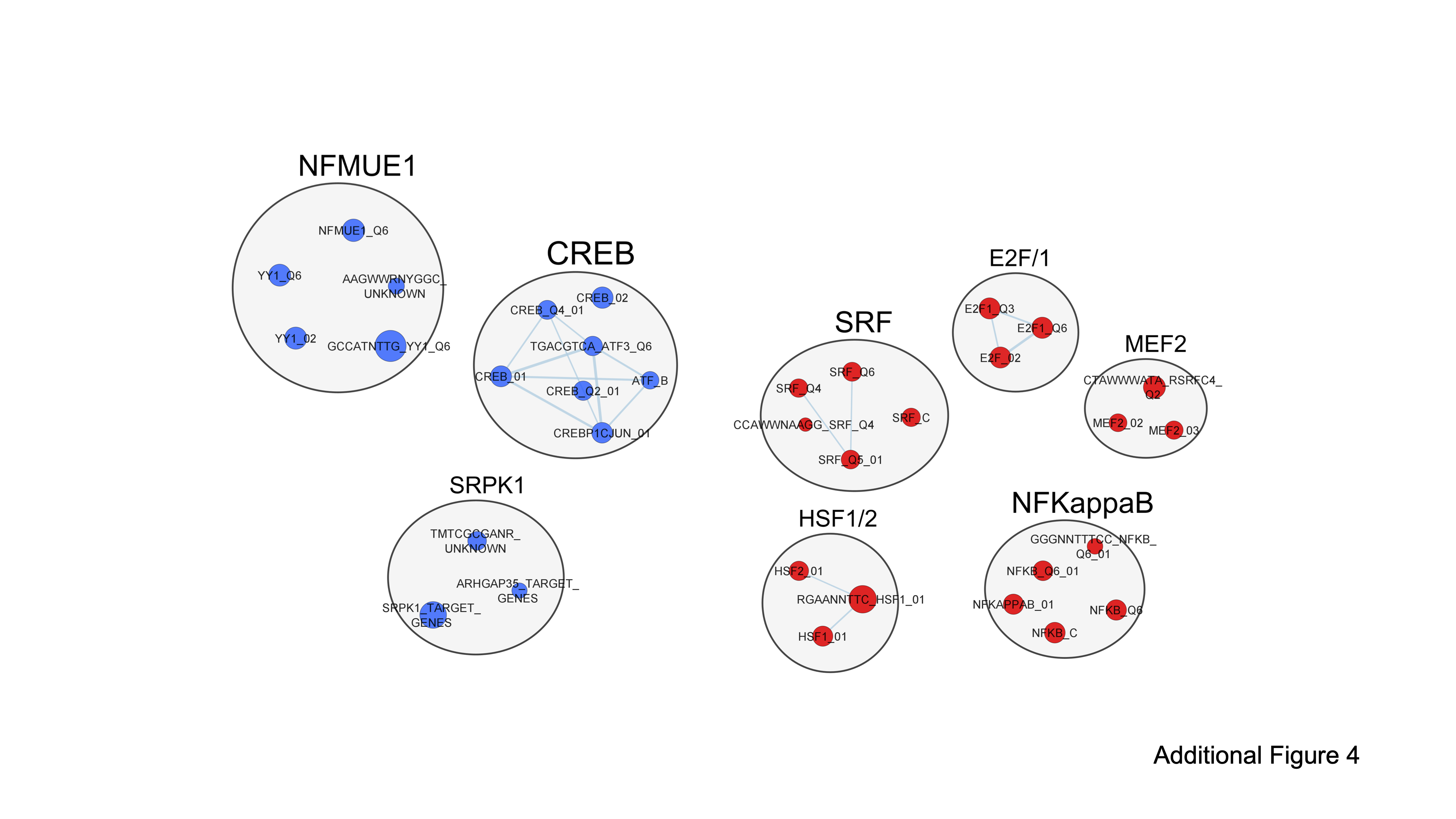

Supplement: Supplementary file 6 — Additional file 6: Figure S4. Transcription factor analysis data set where nodes represent unique gene sets containing genes known to share 5′ cis-regulatory sequences for the binding of specific transcription factors. The edges represent the coefficient of similarity between the gene sets above a defined threshold (Jaccard and overlap similarity coefficient cut-off 0.375). Groups of nodes were obtained by Markov Cluster Algorithm (MCL) clustering into gene modules. The highlighted nodes were associated with a given transcription factor, suggesting a co-regulatory network. Red indicates positive transcript enrichment and blue indicates negative transcript enrichment. [file 40635_2020_361_MOESM6_ESM.tiff]
