# Supplementary material for: Transcriptional profiling of leukocytes in critically ill COVID19 patients: implications for interferon response and coagulation
Source: Intensive Care Med Exp. 2020 Dec 11;8:75. doi: 10.1186/s40635-020-00361-9 (PMC7729690; doi:10.1186/s40635-020-00361-9)
Supplement: Supplementary file 3 — Additional file 3: Table S1. Differentially expressed genes (COVID19 + vs. COVID19-) [file 40635_2020_361_MOESM3_ESM.docx]

**Supplemental Table 1: Differentially expressed genes (COVID19+ vs. COVID19-)**

| \| Gene symbol \| Total counts \| P-value (Pos vs. Neg) \| FDR step up (Pos vs. Neg) \| Ratio (Pos vs. Neg) \| Fold change (Pos vs. Neg) \| \| --- \| --- \| --- \| --- \| --- \| --- \| \| HTR7 \| 1.58E+00 \| 1.75E-03 \| 0.03566312 \| 4.42E-04 \| -2260.8302 \| \| CACNA2D3 \| 9.82E+00 \| 8.18E-04 \| 0.02771433 \| 2.46E-02 \| -40.668672 \| \| APOBEC2 \| 3.77E+00 \| 1.07E-04 \| 0.01508103 \| 4.05E-02 \| -24.714086 \| \| NRG1 \| 4.25E+01 \| 3.72E-05 \| 0.01358748 \| 6.18E-02 \| -16.189495 \| \| TMTC1 \| 2.33E+02 \| 3.19E-04 \| 0.0194621 \| 6.72E-02 \| -14.882466 \| \| ADAMTS5 \| 7.55E+00 \| 4.32E-03 \| 0.05162447 \| 7.53E-02 \| -13.281275 \| \| FAM153A \| 7.28E+00 \| 3.05E-04 \| 0.01914308 \| 7.55E-02 \| -13.244757 \| \| PGA4 \| 3.84E+00 \| 4.87E-03 \| 0.05322635 \| 7.77E-02 \| -12.875518 \| \| KCNH3 \| 1.94E+01 \| 3.39E-03 \| 0.04624744 \| 7.89E-02 \| -12.667216 \| \| RGPD2 \| 1.99E+02 \| 1.86E-03 \| 0.03626429 \| 8.29E-02 \| -12.060069 \| \| PAX8-AS1 \| 2.52E+01 \| 1.00E-03 \| 0.03013087 \| 9.47E-02 \| -10.562071 \| \| HTRA1 \| 1.32E+01 \| 8.68E-04 \| 0.02858274 \| 9.92E-02 \| -10.078284 \| \| NRIP3 \| 6.55E+00 \| 2.83E-04 \| 0.0184668 \| 1.17E-01 \| -8.5622682 \| \| CLEC10A \| 1.62E+01 \| 3.24E-05 \| 0.01358748 \| 1.18E-01 \| -8.4721019 \| \| AMPH \| 2.38E+01 \| 1.67E-03 \| 0.03534598 \| 1.25E-01 \| -8.0117272 \| \| GDF7 \| 5.93E+00 \| 2.09E-04 \| 0.01737605 \| 1.29E-01 \| -7.7377615 \| \| PLIN5 \| 6.95E+01 \| 2.42E-03 \| 0.04084419 \| 1.39E-01 \| -7.19071 \| \| KIAA0408 \| 1.29E+00 \| 4.34E-03 \| 0.05185301 \| 1.52E-01 \| -6.5777076 \| \| CORIN \| 8.33E+00 \| 3.81E-03 \| 0.04858404 \| 1.53E-01 \| -6.5566922 \| \| ST6GALNAC1 \| 2.09E+00 \| 1.66E-04 \| 0.01638822 \| 1.55E-01 \| -6.4391195 \| \| ARPIN-AP3S2 \| 1.57E+01 \| 2.96E-04 \| 0.01892037 \| 1.55E-01 \| -6.4318188 \| \| PAGE2B \| 1.14E+01 \| 2.55E-03 \| 0.04186861 \| 1.56E-01 \| -6.4170762 \| \| RGPD1 \| 5.61E+01 \| 5.26E-04 \| 0.02266365 \| 1.65E-01 \| -6.063037 \| \| FCRL1 \| 1.16E+02 \| 2.40E-05 \| 0.01358748 \| 1.66E-01 \| -6.0347129 \| \| GASK1B \| 1.29E+03 \| 2.71E-05 \| 0.01358748 \| 1.66E-01 \| -6.0125146 \| \| PDE7B \| 5.40E+00 \| 5.44E-05 \| 0.01358748 \| 1.67E-01 \| -6.00261 \| \| GPR15 \| 4.80E+01 \| 3.46E-03 \| 0.04657396 \| 1.70E-01 \| -5.8781297 \| \| VSIG4 \| 8.17E+01 \| 1.77E-03 \| 0.03579792 \| 1.75E-01 \| -5.727779 \| \| EDNRB \| 1.54E+01 \| 8.31E-04 \| 0.02791931 \| 1.77E-01 \| -5.6643239 \| \| SASH1 \| 2.84E+01 \| 8.60E-04 \| 0.02849752 \| 1.87E-01 \| -5.3604371 \| \| IBA57-DT \| 2.19E+00 \| 2.62E-03 \| 0.0424653 \| 1.87E-01 \| -5.3573174 \| \| OR2W3 \| 1.18E+02 \| 2.26E-03 \| 0.0397705 \| 1.87E-01 \| -5.3509741 \| \| CD302 \| 1.02E+03 \| 1.15E-04 \| 0.01508103 \| 1.91E-01 \| -5.2359614 \| \| SPRED1 \| 2.92E+01 \| 1.48E-04 \| 0.01595531 \| 1.94E-01 \| -5.1658798 \| \| SOWAHC \| 1.96E+01 \| 7.57E-05 \| 0.01383902 \| 1.95E-01 \| -5.1162289 \| \| TSPAN5 \| 6.38E+02 \| 3.01E-03 \| 0.04389068 \| 2.05E-01 \| -4.8693613 \| \| APCDD1 \| 5.26E+00 \| 2.83E-03 \| 0.0431483 \| 2.06E-01 \| -4.8652836 \| \| MBNL3 \| 5.20E+03 \| 2.86E-03 \| 0.0431483 \| 2.06E-01 \| -4.8441574 \| \| HNMT \| 1.04E+02 \| 6.28E-05 \| 0.01358748 \| 2.07E-01 \| -4.8384543 \| \| SLC46A2 \| 2.18E+01 \| 1.38E-04 \| 0.01562712 \| 2.08E-01 \| -4.8032126 \| \| PID1 \| 1.18E+01 \| 6.71E-04 \| 0.02505425 \| 2.12E-01 \| -4.7270475 \| \| ASGR1 \| 9.65E+00 \| 1.59E-04 \| 0.01638822 \| 2.12E-01 \| -4.7244887 \| \| RNASE4 \| 5.19E+01 \| 2.30E-04 \| 0.01796904 \| 2.13E-01 \| -4.7011117 \| \| RAPH1 \| 2.56E+01 \| 3.39E-05 \| 0.01358748 \| 2.16E-01 \| -4.6248732 \| \| THBS3 \| 4.23E+01 \| 3.38E-03 \| 0.0462197 \| 2.22E-01 \| -4.5067806 \| \| NFXL1 \| 1.45E+02 \| 8.12E-04 \| 0.02766819 \| 2.23E-01 \| -4.4907929 \| \| TTC9 \| 5.19E+01 \| 1.05E-07 \| 0.00155246 \| 2.23E-01 \| -4.4874813 \| \| PPARG \| 3.35E+01 \| 2.18E-04 \| 0.0175328 \| 2.29E-01 \| -4.3693286 \| \| XKR7 \| 2.88E+00 \| 3.78E-03 \| 0.04843566 \| 2.29E-01 \| -4.369118 \| \| EPS8 \| 1.41E+01 \| 1.68E-04 \| 0.01644262 \| 2.32E-01 \| -4.3185828 \| \| PLCB1 \| 5.87E+01 \| 5.59E-06 \| 0.00733564 \| 2.32E-01 \| -4.3142869 \| \| RNF14 \| 5.34E+02 \| 1.93E-03 \| 0.03701102 \| 2.32E-01 \| -4.3070276 \| \| BCAT1 \| 3.83E+02 \| 3.12E-04 \| 0.01931625 \| 2.37E-01 \| -4.2266527 \| \| LINC00526 \| 2.33E+00 \| 1.81E-03 \| 0.03592144 \| 2.41E-01 \| -4.1559956 \| \| CALCRL \| 3.44E+01 \| 4.24E-03 \| 0.05124371 \| 2.41E-01 \| -4.151503 \| \| GABRR2 \| 3.83E+01 \| 3.05E-03 \| 0.04404908 \| 2.41E-01 \| -4.1467798 \| \| SEMA3C \| 8.27E+01 \| 1.06E-03 \| 0.03043475 \| 2.43E-01 \| -4.1106774 \| \| EPHA1-AS1 \| 1.92E+01 \| 3.64E-03 \| 0.04754042 \| 2.44E-01 \| -4.0947531 \| \| LGALS3 \| 3.85E+02 \| 5.39E-04 \| 0.02271088 \| 2.45E-01 \| -4.0814299 \| \| PDK4 \| 3.15E+02 \| 4.37E-05 \| 0.01358748 \| 2.53E-01 \| -3.9587478 \| \| CYP2D7 \| 3.73E+00 \| 2.97E-03 \| 0.0437064 \| 2.55E-01 \| -3.9274607 \| \| GPR82 \| 5.74E+00 \| 3.87E-03 \| 0.04890098 \| 2.55E-01 \| -3.9158555 \| \| FAM20A \| 3.43E+01 \| 1.83E-04 \| 0.01716907 \| 2.56E-01 \| -3.9013493 \| \| PLB1 \| 1.90E+02 \| 3.13E-06 \| 0.00733564 \| 2.57E-01 \| -3.8960942 \| \| PTPN13 \| 1.80E+01 \| 6.49E-06 \| 0.00733564 \| 2.59E-01 \| -3.8546418 \| \| ENC1 \| 1.85E+02 \| 4.26E-03 \| 0.05129215 \| 2.61E-01 \| -3.8297957 \| \| SIRPB3P \| 9.76E+00 \| 1.78E-03 \| 0.035844 \| 2.63E-01 \| -3.8037266 \| \| SEPTIN10 \| 2.57E+01 \| 6.92E-04 \| 0.02518353 \| 2.64E-01 \| -3.7901913 \| \| SLC37A2 \| 4.28E+01 \| 2.41E-07 \| 0.00178617 \| 2.66E-01 \| -3.7643378 \| \| MYO7A \| 1.35E+01 \| 1.71E-03 \| 0.03557751 \| 2.67E-01 \| -3.7476096 \| \| BAG1 \| 4.89E+02 \| 4.20E-04 \| 0.02154268 \| 2.71E-01 \| -3.6842286 \| \| TCP11L2 \| 1.63E+03 \| 2.03E-04 \| 0.01727339 \| 2.72E-01 \| -3.6744148 \| \| STK32B \| 5.96E+00 \| 2.67E-03 \| 0.04281202 \| 2.73E-01 \| -3.6683504 \| \| SMPDL3A \| 2.36E+01 \| 2.12E-04 \| 0.01742995 \| 2.73E-01 \| -3.66624 \| \| SH3PXD2B \| 3.33E+01 \| 4.56E-03 \| 0.05231298 \| 2.74E-01 \| -3.650732 \| \| SEMA6B \| 8.01E+00 \| 1.58E-03 \| 0.03496769 \| 2.74E-01 \| -3.6496185 \| \| DSC2 \| 8.81E+02 \| 1.57E-03 \| 0.03496769 \| 2.75E-01 \| -3.6377594 \| \| IRF6 \| 2.94E+00 \| 4.62E-03 \| 0.05256331 \| 2.75E-01 \| -3.6361899 \| \| TAGLN \| 9.66E+00 \| 2.65E-04 \| 0.01810876 \| 2.77E-01 \| -3.6134944 \| \| NMNAT2 \| 4.61E+00 \| 3.47E-03 \| 0.04665936 \| 2.77E-01 \| -3.6110481 \| \| SLC12A9 \| 1.88E+02 \| 1.97E-04 \| 0.01723175 \| 2.77E-01 \| -3.608359 \| \| TGFBI \| 2.38E+02 \| 1.62E-04 \| 0.01638822 \| 2.80E-01 \| -3.5688508 \| \| RUBCNL \| 2.78E+02 \| 1.42E-03 \| 0.03422635 \| 2.81E-01 \| -3.5587969 \| \| LINC01006 \| 3.46E+00 \| 1.78E-03 \| 0.035844 \| 2.84E-01 \| -3.5188675 \| \| H2BU1 \| 4.16E+01 \| 1.09E-03 \| 0.03064526 \| 2.88E-01 \| -3.4701206 \| \| CPVL \| 3.63E+02 \| 3.24E-04 \| 0.01959571 \| 2.93E-01 \| -3.4183583 \| \| LINC00926 \| 1.93E+01 \| 1.81E-03 \| 0.03592144 \| 2.93E-01 \| -3.4168712 \| \| RNASE6 \| 1.42E+02 \| 1.66E-04 \| 0.01638822 \| 2.93E-01 \| -3.4135118 \| \| SLC25A37 \| 1.31E+04 \| 1.47E-03 \| 0.03434026 \| 2.94E-01 \| -3.4024118 \| \| MARCO \| 1.88E+01 \| 2.03E-03 \| 0.03795385 \| 2.95E-01 \| -3.3921216 \| \| RNU1-3 \| 9.94E+01 \| 1.60E-03 \| 0.03496769 \| 2.98E-01 \| -3.3610907 \| \| RNVU1-18 \| 9.94E+01 \| 1.60E-03 \| 0.03496769 \| 2.98E-01 \| -3.3610907 \| \| RNU1-4 \| 9.94E+01 \| 1.60E-03 \| 0.03496769 \| 2.98E-01 \| -3.3610907 \| \| RNU1-2 \| 9.94E+01 \| 1.60E-03 \| 0.03496769 \| 2.98E-01 \| -3.3610907 \| \| RNU1-1 \| 9.94E+01 \| 1.60E-03 \| 0.03496769 \| 2.98E-01 \| -3.3610907 \| \| TRIM2 \| 1.23E+01 \| 2.64E-03 \| 0.04256329 \| 2.98E-01 \| -3.3550158 \| \| RASSF7 \| 3.50E+00 \| 2.67E-03 \| 0.04278542 \| 2.98E-01 \| -3.3532702 \| \| GSTZ1 \| 6.83E+00 \| 2.36E-04 \| 0.01796904 \| 3.00E-01 \| -3.3311263 \| \| VCAN \| 2.05E+04 \| 2.14E-04 \| 0.01742995 \| 3.02E-01 \| -3.3061868 \| \| TNFRSF9 \| 4.77E+01 \| 4.84E-04 \| 0.0222881 \| 3.07E-01 \| -3.2616893 \| \| MS4A7 \| 2.10E+02 \| 2.74E-05 \| 0.01358748 \| 3.08E-01 \| -3.2465902 \| \| FAM198B-AS1 \| 8.61E+01 \| 8.44E-05 \| 0.01407615 \| 3.09E-01 \| -3.2383794 \| \| BPHL \| 4.30E+00 \| 5.51E-04 \| 0.02272904 \| 3.10E-01 \| -3.2267312 \| \| CYBRD1 \| 4.77E+02 \| 1.85E-03 \| 0.03625874 \| 3.10E-01 \| -3.2235223 \| \| TMEM144 \| 4.83E+01 \| 6.71E-04 \| 0.02505425 \| 3.10E-01 \| -3.2213104 \| \| REREP3 \| 1.63E+00 \| 9.60E-04 \| 0.0293236 \| 3.10E-01 \| -3.2211946 \| \| DHX32 \| 4.73E+01 \| 1.82E-03 \| 0.03592144 \| 3.11E-01 \| -3.2189412 \| \| MS4A4A \| 8.69E+01 \| 1.52E-03 \| 0.03454272 \| 3.11E-01 \| -3.2150783 \| \| HFE \| 1.59E+01 \| 2.42E-04 \| 0.01800472 \| 3.11E-01 \| -3.2132055 \| \| CCR2 \| 1.33E+03 \| 1.11E-03 \| 0.03091554 \| 3.13E-01 \| -3.1997553 \| \| SLITRK4 \| 5.13E+01 \| 3.17E-03 \| 0.04497545 \| 3.13E-01 \| -3.1946055 \| \| SLC27A1 \| 1.27E+01 \| 6.41E-05 \| 0.01358748 \| 3.15E-01 \| -3.1729646 \| \| TSPYL5 \| 7.62E+00 \| 4.62E-04 \| 0.02201345 \| 3.17E-01 \| -3.1532853 \| \| FMC1-LUC7L2 \| 1.91E+01 \| 9.12E-04 \| 0.02864918 \| 3.18E-01 \| -3.1422114 \| \| MICALCL \| 2.13E+02 \| 2.65E-03 \| 0.042717 \| 3.19E-01 \| -3.1390388 \| \| LTA4H \| 1.78E+03 \| 5.36E-06 \| 0.00733564 \| 3.21E-01 \| -3.1164649 \| \| CCDC170 \| 2.56E+01 \| 1.12E-04 \| 0.01508103 \| 3.23E-01 \| -3.0924144 \| \| RPS14 \| 1.45E+03 \| 4.98E-03 \| 0.05366228 \| 3.27E-01 \| -3.05911 \| \| B3GNTL1 \| 6.28E+01 \| 5.81E-04 \| 0.02325349 \| 3.28E-01 \| -3.0531365 \| \| ARRDC4 \| 2.41E+02 \| 4.60E-04 \| 0.02201345 \| 3.28E-01 \| -3.0519467 \| \| MYCL \| 4.88E+01 \| 1.23E-04 \| 0.01509922 \| 3.28E-01 \| -3.0485166 \| \| SLC38A6 \| 2.04E+01 \| 3.15E-03 \| 0.04486926 \| 3.29E-01 \| -3.0419411 \| \| LOC100288069 \| 8.68E+01 \| 1.93E-03 \| 0.03701102 \| 3.30E-01 \| -3.0306365 \| \| ARPIN \| 2.87E+01 \| 1.39E-04 \| 0.0156319 \| 3.30E-01 \| -3.028225 \| \| TM7SF2 \| 1.21E+01 \| 1.91E-03 \| 0.03697811 \| 3.31E-01 \| -3.0243903 \| \| KCTD15 \| 1.33E+01 \| 1.37E-03 \| 0.03405687 \| 3.36E-01 \| -2.9804741 \| \| TUBBP5 \| 6.75E+00 \| 6.31E-04 \| 0.0243819 \| 3.38E-01 \| -2.9622144 \| \| TXNRD2 \| 3.94E+01 \| 4.78E-04 \| 0.02227986 \| 3.39E-01 \| -2.9496501 \| \| NUDT14 \| 2.88E+00 \| 2.93E-03 \| 0.04348498 \| 3.40E-01 \| -2.9415069 \| \| TPT1 \| 1.10E+04 \| 2.73E-03 \| 0.0429721 \| 3.41E-01 \| -2.9361063 \| \| PELI3 \| 1.57E+01 \| 1.13E-05 \| 0.01073864 \| 3.41E-01 \| -2.9348287 \| \| ASB13 \| 1.74E+01 \| 2.12E-03 \| 0.03843036 \| 3.42E-01 \| -2.9265343 \| \| CSF1R \| 2.67E+02 \| 3.98E-06 \| 0.00733564 \| 3.42E-01 \| -2.9245436 \| \| CPM \| 6.26E+01 \| 3.03E-03 \| 0.04404095 \| 3.42E-01 \| -2.922553 \| \| SUMF1 \| 1.07E+02 \| 4.31E-04 \| 0.02156426 \| 3.43E-01 \| -2.9189435 \| \| LINC01503 \| 9.40E+00 \| 4.46E-03 \| 0.05222976 \| 3.43E-01 \| -2.9165597 \| \| PLD2 \| 2.16E+01 \| 4.84E-05 \| 0.01358748 \| 3.43E-01 \| -2.913748 \| \| LRP1 \| 1.63E+03 \| 1.00E-04 \| 0.01508103 \| 3.43E-01 \| -2.9125258 \| \| CAMK1D \| 3.37E+02 \| 5.78E-05 \| 0.01358748 \| 3.44E-01 \| -2.9085212 \| \| RBM3 \| 3.61E+02 \| 2.82E-03 \| 0.0431483 \| 3.44E-01 \| -2.9066219 \| \| KLF11 \| 9.23E+01 \| 6.24E-06 \| 0.00733564 \| 3.47E-01 \| -2.8853446 \| \| ZFHX3 \| 1.13E+02 \| 1.75E-05 \| 0.0109766 \| 3.47E-01 \| -2.8788889 \| \| AHR \| 7.87E+02 \| 2.33E-04 \| 0.01796904 \| 3.48E-01 \| -2.8733413 \| \| WDR45 \| 2.81E+02 \| 2.30E-03 \| 0.03980839 \| 3.49E-01 \| -2.8669036 \| \| AMPD2 \| 2.37E+02 \| 4.04E-04 \| 0.0212069 \| 3.50E-01 \| -2.8561154 \| \| MTMR8 \| 9.02E+00 \| 2.76E-04 \| 0.01841921 \| 3.52E-01 \| -2.8445456 \| \| NCEH1 \| 1.06E+02 \| 3.38E-04 \| 0.01980958 \| 3.52E-01 \| -2.843617 \| \| VENTX \| 9.33E+00 \| 1.68E-03 \| 0.0354207 \| 3.53E-01 \| -2.8360449 \| \| SETD7 \| 3.41E+02 \| 3.29E-03 \| 0.04567974 \| 3.53E-01 \| -2.834624 \| \| LOC100289473 \| 3.35E+01 \| 1.81E-03 \| 0.03592144 \| 3.54E-01 \| -2.82855 \| \| SOCS6 \| 5.14E+01 \| 1.77E-03 \| 0.03579792 \| 3.54E-01 \| -2.8254258 \| \| ADCK5 \| 2.45E+00 \| 3.42E-03 \| 0.04632133 \| 3.54E-01 \| -2.825422 \| \| LILRB1 \| 9.79E+01 \| 4.81E-03 \| 0.05307894 \| 3.55E-01 \| -2.8143898 \| \| POMT2 \| 1.01E+01 \| 3.58E-03 \| 0.04729405 \| 3.55E-01 \| -2.8134768 \| \| MPEG1 \| 2.45E+03 \| 6.93E-06 \| 0.00733564 \| 3.56E-01 \| -2.812396 \| \| HLA-DQA1 \| 1.19E+02 \| 4.01E-04 \| 0.0212069 \| 3.56E-01 \| -2.8062447 \| \| PTRHD1 \| 3.37E+01 \| 4.39E-03 \| 0.05197104 \| 3.57E-01 \| -2.8030451 \| \| KLHDC2 \| 1.39E+02 \| 4.71E-03 \| 0.05303553 \| 3.57E-01 \| -2.8009719 \| \| ALDH2 \| 2.42E+02 \| 9.76E-04 \| 0.02948669 \| 3.58E-01 \| -2.7954594 \| \| ANO9 \| 3.94E+00 \| 2.27E-03 \| 0.0397705 \| 3.58E-01 \| -2.7906745 \| \| SLC46A1 \| 6.70E+00 \| 2.36E-03 \| 0.040317 \| 3.60E-01 \| -2.7795364 \| \| MTMR11 \| 3.89E+01 \| 3.19E-03 \| 0.04504367 \| 3.60E-01 \| -2.7793941 \| \| FLJ44635 \| 1.70E+02 \| 1.47E-03 \| 0.03434026 \| 3.60E-01 \| -2.7775433 \| \| CD44 \| 3.89E+03 \| 8.29E-05 \| 0.01407615 \| 3.64E-01 \| -2.7494766 \| \| CYP1B1 \| 1.74E+03 \| 1.53E-03 \| 0.03462625 \| 3.64E-01 \| -2.7466144 \| \| PALLD \| 2.31E+01 \| 1.40E-03 \| 0.03422635 \| 3.64E-01 \| -2.7435941 \| \| ZNF697 \| 5.37E+01 \| 1.45E-03 \| 0.03434026 \| 3.65E-01 \| -2.7427622 \| \| SNX22 \| 1.36E+01 \| 2.70E-03 \| 0.04289637 \| 3.65E-01 \| -2.7423561 \| \| NLRP1 \| 6.61E+02 \| 3.33E-03 \| 0.0458714 \| 3.65E-01 \| -2.7412246 \| \| TBC1D9 \| 1.84E+02 \| 6.37E-06 \| 0.00733564 \| 3.65E-01 \| -2.7407966 \| \| TEPSIN \| 2.21E+01 \| 2.06E-03 \| 0.03807473 \| 3.66E-01 \| -2.7296775 \| \| SLC7A7 \| 3.76E+02 \| 7.50E-04 \| 0.02646243 \| 3.68E-01 \| -2.7206088 \| \| CLMN \| 1.48E+02 \| 1.01E-04 \| 0.01508103 \| 3.69E-01 \| -2.7125382 \| \| CDK5 \| 1.30E+01 \| 1.08E-03 \| 0.03063671 \| 3.69E-01 \| -2.7064727 \| \| IMPDH1 \| 3.31E+02 \| 1.58E-03 \| 0.03496769 \| 3.71E-01 \| -2.6982262 \| \| SUN1 \| 8.97E+01 \| 3.47E-05 \| 0.01358748 \| 3.71E-01 \| -2.6980454 \| \| AHNAK \| 1.19E+04 \| 1.00E-04 \| 0.01508103 \| 3.73E-01 \| -2.6817951 \| \| MCOLN1 \| 9.24E+01 \| 1.34E-03 \| 0.03373293 \| 3.73E-01 \| -2.6790421 \| \| MERTK \| 5.07E+01 \| 3.42E-03 \| 0.04632133 \| 3.73E-01 \| -2.6781202 \| \| CLCN5 \| 9.20E+01 \| 1.46E-04 \| 0.01584897 \| 3.74E-01 \| -2.6761399 \| \| DPH6 \| 1.74E+01 \| 6.86E-05 \| 0.01358748 \| 3.74E-01 \| -2.6744826 \| \| ALCAM \| 2.29E+02 \| 2.27E-04 \| 0.01796904 \| 3.76E-01 \| -2.6623581 \| \| TG \| 9.15E+01 \| 3.63E-03 \| 0.04741929 \| 3.78E-01 \| -2.6449328 \| \| RPGR \| 1.02E+02 \| 3.54E-05 \| 0.01358748 \| 3.79E-01 \| -2.6401754 \| \| SLC9A7P1 \| 9.23E+01 \| 4.43E-04 \| 0.02187475 \| 3.79E-01 \| -2.6392815 \| \| CTSK \| 1.76E+01 \| 2.99E-03 \| 0.04383474 \| 3.79E-01 \| -2.6363263 \| \| ALKBH7 \| 1.37E+01 \| 4.23E-03 \| 0.05120325 \| 3.80E-01 \| -2.6309494 \| \| TMEM168 \| 1.38E+02 \| 1.61E-05 \| 0.01082113 \| 3.82E-01 \| -2.6198683 \| \| PCMTD2 \| 3.02E+02 \| 4.07E-04 \| 0.02122812 \| 3.82E-01 \| -2.6158188 \| \| SLC8A1 \| 1.71E+03 \| 1.13E-04 \| 0.01508103 \| 3.83E-01 \| -2.61117 \| \| LILRA1 \| 4.00E+02 \| 4.91E-04 \| 0.0222881 \| 3.83E-01 \| -2.6081699 \| \| CACNA2D4 \| 1.92E+01 \| 4.91E-04 \| 0.0222881 \| 3.85E-01 \| -2.5941313 \| \| CERK \| 2.85E+02 \| 5.59E-04 \| 0.02281591 \| 3.87E-01 \| -2.5812352 \| \| TAZ \| 3.69E+01 \| 5.01E-03 \| 0.05376205 \| 3.88E-01 \| -2.5800435 \| \| EIF3L \| 4.13E+02 \| 3.26E-05 \| 0.01358748 \| 3.88E-01 \| -2.5756209 \| \| COQ8A \| 7.52E+01 \| 7.92E-05 \| 0.01392625 \| 3.89E-01 \| -2.5737679 \| \| GALK1 \| 2.62E+01 \| 9.68E-04 \| 0.02938995 \| 3.89E-01 \| -2.5703483 \| \| CAT \| 1.02E+03 \| 1.17E-03 \| 0.03155022 \| 3.89E-01 \| -2.5701306 \| \| TMCO3 \| 2.51E+02 \| 1.05E-03 \| 0.03041505 \| 3.90E-01 \| -2.5669729 \| \| IMPA2 \| 1.27E+02 \| 6.10E-04 \| 0.0239563 \| 3.90E-01 \| -2.565573 \| \| RMND5A \| 1.68E+03 \| 2.42E-03 \| 0.04084419 \| 3.90E-01 \| -2.5627873 \| \| CTSB \| 1.75E+03 \| 1.55E-05 \| 0.01082113 \| 3.91E-01 \| -2.5573157 \| \| OPN3 \| 5.83E+01 \| 2.80E-05 \| 0.01358748 \| 3.93E-01 \| -2.5433033 \| \| DPH5 \| 3.49E+01 \| 2.60E-03 \| 0.04214926 \| 3.93E-01 \| -2.5422316 \| \| RAD23A \| 2.39E+02 \| 2.86E-03 \| 0.0431483 \| 3.95E-01 \| -2.5329507 \| \| METRNL \| 1.25E+02 \| 3.66E-04 \| 0.02063449 \| 3.95E-01 \| -2.5285395 \| \| SLC4A7 \| 1.96E+02 \| 1.91E-04 \| 0.01723175 \| 3.96E-01 \| -2.5272202 \| \| EPM2A \| 1.90E+01 \| 5.59E-04 \| 0.02281591 \| 3.97E-01 \| -2.5184965 \| \| PTPRO \| 6.29E+01 \| 1.28E-04 \| 0.01528723 \| 3.98E-01 \| -2.5145667 \| \| GHITM \| 6.61E+02 \| 2.17E-03 \| 0.03890483 \| 3.98E-01 \| -2.5127929 \| \| TRIM36 \| 1.07E+01 \| 4.62E-03 \| 0.05256331 \| 3.99E-01 \| -2.5079172 \| \| CD300E \| 2.13E+02 \| 8.90E-04 \| 0.02862553 \| 3.99E-01 \| -2.5078415 \| \| LPCAT3 \| 1.25E+02 \| 1.63E-04 \| 0.01638822 \| 3.99E-01 \| -2.5077295 \| \| ASGR2 \| 4.55E+01 \| 6.81E-04 \| 0.02508175 \| 3.99E-01 \| -2.5065513 \| \| DEF8 \| 3.03E+02 \| 5.46E-04 \| 0.02271088 \| 3.99E-01 \| -2.5061119 \| \| MRPS17 \| 1.32E+01 \| 5.50E-04 \| 0.02272904 \| 4.00E-01 \| -2.5008864 \| \| SNX30 \| 3.26E+02 \| 4.71E-05 \| 0.01358748 \| 4.00E-01 \| -2.5008091 \| \| DHX40 \| 3.09E+02 \| 5.85E-04 \| 0.02335624 \| 4.00E-01 \| -2.5000543 \| \| MGST1 \| 1.03E+02 \| 4.98E-04 \| 0.02234623 \| 4.00E-01 \| -2.4974027 \| \| FAM104B \| 1.91E+01 \| 3.73E-03 \| 0.04789609 \| 4.01E-01 \| -2.493527 \| \| MAP3K14 \| 4.71E+01 \| 1.11E-04 \| 0.01508103 \| 4.01E-01 \| -2.4907405 \| \| FUCA1 \| 6.78E+01 \| 9.60E-04 \| 0.0293236 \| 4.03E-01 \| -2.4835091 \| \| RPS16 \| 6.09E+02 \| 4.93E-03 \| 0.0534573 \| 4.03E-01 \| -2.4834155 \| \| TNFSF13 \| 1.45E+02 \| 4.35E-03 \| 0.05185301 \| 4.03E-01 \| -2.4824404 \| \| IRF8 \| 2.48E+02 \| 1.89E-04 \| 0.01718472 \| 4.04E-01 \| -2.4743522 \| \| MOB3B \| 4.44E+01 \| 2.36E-03 \| 0.04025965 \| 4.04E-01 \| -2.4736732 \| \| TTC30A \| 1.81E+01 \| 2.19E-03 \| 0.03918397 \| 4.05E-01 \| -2.4710993 \| \| SLC11A1 \| 8.89E+02 \| 2.74E-03 \| 0.0429721 \| 4.05E-01 \| -2.4679657 \| \| SLC26A6 \| 3.74E+01 \| 2.51E-03 \| 0.04147083 \| 4.05E-01 \| -2.4660994 \| \| D2HGDH \| 5.40E+00 \| 4.74E-03 \| 0.05304222 \| 4.06E-01 \| -2.4653703 \| \| CA5B \| 4.67E+01 \| 3.63E-03 \| 0.04741929 \| 4.07E-01 \| -2.4570957 \| \| TEN1 \| 1.13E+01 \| 2.06E-03 \| 0.03807473 \| 4.08E-01 \| -2.4531227 \| \| XRN2 \| 7.32E+02 \| 5.62E-04 \| 0.02281742 \| 4.08E-01 \| -2.4514255 \| \| FMO5 \| 3.27E+01 \| 2.49E-03 \| 0.04130559 \| 4.08E-01 \| -2.448792 \| \| CCNDBP1 \| 8.81E+02 \| 2.57E-03 \| 0.04191493 \| 4.10E-01 \| -2.4393525 \| \| RAB36 \| 1.34E+01 \| 4.82E-03 \| 0.05307894 \| 4.11E-01 \| -2.4337495 \| \| ERCC8 \| 3.03E+01 \| 1.64E-03 \| 0.03532746 \| 4.12E-01 \| -2.4294338 \| \| FCGRT \| 3.18E+02 \| 7.95E-05 \| 0.01392625 \| 4.12E-01 \| -2.4244883 \| \| ANKRD50 \| 2.43E+02 \| 1.14E-03 \| 0.03112937 \| 4.13E-01 \| -2.4191518 \| \| LRP3 \| 7.11E+00 \| 3.49E-03 \| 0.0467129 \| 4.14E-01 \| -2.4136593 \| \| BMF \| 1.17E+02 \| 1.43E-05 \| 0.01073864 \| 4.15E-01 \| -2.4092284 \| \| EEF1A1 \| 1.22E+04 \| 2.46E-03 \| 0.041125 \| 4.16E-01 \| -2.4062622 \| \| JAML \| 1.16E+03 \| 7.37E-04 \| 0.02629382 \| 4.16E-01 \| -2.4056315 \| \| RAB34 \| 2.60E+01 \| 2.88E-03 \| 0.04331108 \| 4.16E-01 \| -2.4052611 \| \| TIAM2 \| 2.71E+01 \| 2.28E-03 \| 0.03979599 \| 4.16E-01 \| -2.402754 \| \| SIRPD \| 4.14E+01 \| 2.25E-03 \| 0.0397705 \| 4.16E-01 \| -2.402114 \| \| RPL23 \| 8.33E+02 \| 3.20E-03 \| 0.04504367 \| 4.17E-01 \| -2.4008989 \| \| NBPF9 \| 2.06E+02 \| 1.16E-03 \| 0.03152193 \| 4.17E-01 \| -2.3988764 \| \| CREBL2 \| 2.49E+02 \| 3.32E-03 \| 0.04581592 \| 4.19E-01 \| -2.3862126 \| \| NEK8 \| 6.13E+00 \| 2.04E-03 \| 0.03806237 \| 4.20E-01 \| -2.3832734 \| \| SLC16A7 \| 4.85E+02 \| 6.50E-05 \| 0.01358748 \| 4.20E-01 \| -2.3831116 \| \| PEPD \| 4.64E+01 \| 3.68E-04 \| 0.02063449 \| 4.20E-01 \| -2.3822421 \| \| GLIPR1 \| 1.31E+03 \| 4.60E-03 \| 0.05256331 \| 4.20E-01 \| -2.3811117 \| \| ATRN \| 1.71E+02 \| 1.24E-04 \| 0.01509922 \| 4.20E-01 \| -2.3795494 \| \| TMTC2 \| 9.00E+01 \| 2.03E-04 \| 0.01727339 \| 4.20E-01 \| -2.3787697 \| \| SLC25A40 \| 2.91E+02 \| 4.55E-03 \| 0.05231298 \| 4.21E-01 \| -2.3774977 \| \| BTF3 \| 1.15E+03 \| 1.14E-03 \| 0.0310461 \| 4.21E-01 \| -2.37629 \| \| SLC8B1 \| 1.26E+02 \| 2.94E-04 \| 0.01891181 \| 4.21E-01 \| -2.3736528 \| \| UBXN11 \| 4.81E+01 \| 1.64E-03 \| 0.03532746 \| 4.23E-01 \| -2.3667143 \| \| NPEPL1 \| 6.62E+01 \| 1.26E-03 \| 0.03281376 \| 4.23E-01 \| -2.3658136 \| \| PLXND1 \| 9.14E+01 \| 1.40E-03 \| 0.03422635 \| 4.23E-01 \| -2.3645742 \| \| ADK \| 5.93E+01 \| 1.60E-03 \| 0.03500513 \| 4.23E-01 \| -2.3621679 \| \| NBPF11 \| 1.24E+02 \| 6.38E-05 \| 0.01358748 \| 4.24E-01 \| -2.3582979 \| \| FLVCR2 \| 9.45E+01 \| 4.78E-04 \| 0.02227986 \| 4.24E-01 \| -2.3576115 \| \| PPM1F \| 4.37E+02 \| 2.47E-04 \| 0.01800472 \| 4.24E-01 \| -2.3571931 \| \| NAAA \| 1.27E+02 \| 1.95E-04 \| 0.01723175 \| 4.25E-01 \| -2.3542064 \| \| LPAR1 \| 5.62E+01 \| 3.33E-03 \| 0.0458714 \| 4.25E-01 \| -2.3536905 \| \| TSPAN3 \| 5.88E+01 \| 1.08E-03 \| 0.03063671 \| 4.25E-01 \| -2.3530965 \| \| MDFIC \| 2.27E+02 \| 9.10E-04 \| 0.02864918 \| 4.26E-01 \| -2.3496212 \| \| NIBAN2 \| 8.63E+01 \| 2.34E-04 \| 0.01796904 \| 4.26E-01 \| -2.3447478 \| \| SGPL1 \| 2.47E+02 \| 7.36E-04 \| 0.02629382 \| 4.27E-01 \| -2.3423868 \| \| HAUS7 \| 1.12E+01 \| 5.59E-04 \| 0.02281591 \| 4.27E-01 \| -2.3393839 \| \| CD1D \| 1.08E+02 \| 4.30E-04 \| 0.02156426 \| 4.29E-01 \| -2.3316252 \| \| RPL15 \| 1.80E+03 \| 2.26E-03 \| 0.0397705 \| 4.29E-01 \| -2.3306683 \| \| CCDC88A \| 4.04E+02 \| 8.82E-04 \| 0.02862553 \| 4.30E-01 \| -2.3275122 \| \| CUL4A \| 3.85E+02 \| 1.31E-03 \| 0.03339217 \| 4.30E-01 \| -2.3263844 \| \| KCTD17 \| 4.13E+00 \| 2.25E-03 \| 0.0397705 \| 4.31E-01 \| -2.3224014 \| \| MTMR1 \| 2.00E+02 \| 1.25E-03 \| 0.03271222 \| 4.31E-01 \| -2.3215597 \| \| CCDC124 \| 2.60E+01 \| 1.23E-03 \| 0.03244251 \| 4.31E-01 \| -2.3208188 \| \| TMEM167A \| 1.01E+03 \| 1.43E-03 \| 0.03422635 \| 4.31E-01 \| -2.3175149 \| \| ZNF785 \| 9.21E+00 \| 2.59E-04 \| 0.01801198 \| 4.32E-01 \| -2.3159741 \| \| PGP \| 1.47E+01 \| 4.88E-03 \| 0.05322635 \| 4.32E-01 \| -2.3122953 \| \| ULK2 \| 5.01E+01 \| 2.67E-04 \| 0.01810876 \| 4.33E-01 \| -2.3102288 \| \| SLC25A24 \| 4.76E+02 \| 3.07E-03 \| 0.04416241 \| 4.34E-01 \| -2.3052881 \| \| LOC110091768 \| 5.24E+00 \| 4.87E-03 \| 0.05322635 \| 4.34E-01 \| -2.3051925 \| \| ACP3 \| 2.40E+02 \| 2.25E-03 \| 0.0397705 \| 4.34E-01 \| -2.3040151 \| \| TRPC1 \| 1.23E+01 \| 4.20E-03 \| 0.05101218 \| 4.34E-01 \| -2.3034378 \| \| EML3 \| 6.71E+01 \| 2.45E-04 \| 0.01800472 \| 4.35E-01 \| -2.3006325 \| \| ZNF558 \| 3.78E+01 \| 8.09E-04 \| 0.02766819 \| 4.37E-01 \| -2.2891133 \| \| TMEM70 \| 9.40E+01 \| 1.72E-03 \| 0.03557815 \| 4.37E-01 \| -2.2889525 \| \| COLGALT1 \| 3.11E+02 \| 1.75E-03 \| 0.03566312 \| 4.37E-01 \| -2.2879454 \| \| CPNE8 \| 1.29E+02 \| 2.74E-03 \| 0.0429721 \| 4.37E-01 \| -2.2874191 \| \| RHOT2 \| 4.00E+01 \| 3.08E-03 \| 0.04416241 \| 4.37E-01 \| -2.2872638 \| \| PPT1 \| 1.45E+03 \| 4.34E-03 \| 0.05185301 \| 4.37E-01 \| -2.2868355 \| \| EMC3 \| 6.82E+02 \| 3.21E-03 \| 0.04507005 \| 4.38E-01 \| -2.2823665 \| \| METTL17 \| 1.71E+01 \| 4.42E-03 \| 0.05205649 \| 4.40E-01 \| -2.2732542 \| \| TNFRSF10B \| 2.13E+02 \| 5.03E-04 \| 0.02238866 \| 4.41E-01 \| -2.2686807 \| \| DDX28 \| 2.69E+01 \| 1.98E-03 \| 0.03723394 \| 4.41E-01 \| -2.2663191 \| \| NUDT3 \| 4.72E+02 \| 5.24E-04 \| 0.02266365 \| 4.41E-01 \| -2.2654453 \| \| TPK1 \| 8.01E+01 \| 1.83E-04 \| 0.01716907 \| 4.41E-01 \| -2.2650506 \| \| DUSP12 \| 2.06E+01 \| 2.27E-03 \| 0.0397705 \| 4.42E-01 \| -2.2645178 \| \| BCS1L \| 1.22E+01 \| 4.45E-03 \| 0.05222976 \| 4.42E-01 \| -2.2618441 \| \| RPS7 \| 4.86E+02 \| 1.33E-03 \| 0.03352942 \| 4.43E-01 \| -2.2587441 \| \| GPCPD1 \| 1.61E+03 \| 1.01E-03 \| 0.03013087 \| 4.43E-01 \| -2.2555642 \| \| PLXNA2 \| 4.51E+01 \| 3.41E-04 \| 0.01982471 \| 4.44E-01 \| -2.2546731 \| \| TBL1XR1 \| 1.67E+03 \| 2.16E-03 \| 0.03890483 \| 4.44E-01 \| -2.2516963 \| \| STAB1 \| 2.74E+02 \| 1.09E-03 \| 0.03064526 \| 4.45E-01 \| -2.2462078 \| \| EVI5 \| 2.91E+02 \| 1.44E-04 \| 0.01579217 \| 4.45E-01 \| -2.2450905 \| \| SNTB2 \| 2.88E+02 \| 2.93E-03 \| 0.04348498 \| 4.46E-01 \| -2.2424962 \| \| PDSS1 \| 4.16E+01 \| 1.93E-03 \| 0.03697811 \| 4.46E-01 \| -2.2422311 \| \| ZBTB22 \| 5.08E+01 \| 9.09E-04 \| 0.02864918 \| 4.46E-01 \| -2.2415697 \| \| HVCN1 \| 1.07E+02 \| 4.53E-04 \| 0.02201345 \| 4.46E-01 \| -2.24134 \| \| SLC39A1 \| 1.52E+02 \| 1.76E-03 \| 0.03573192 \| 4.46E-01 \| -2.2400894 \| \| FBXO9 \| 4.33E+02 \| 1.08E-04 \| 0.01508103 \| 4.47E-01 \| -2.2390632 \| \| PDP1 \| 2.90E+02 \| 1.08E-03 \| 0.03063671 \| 4.48E-01 \| -2.2339731 \| \| FAM135A \| 7.54E+01 \| 1.13E-03 \| 0.0310461 \| 4.48E-01 \| -2.2332121 \| \| TMED8 \| 7.85E+02 \| 3.08E-03 \| 0.04416241 \| 4.48E-01 \| -2.232551 \| \| NAGA \| 2.45E+02 \| 1.14E-04 \| 0.01508103 \| 4.48E-01 \| -2.2310316 \| \| C12orf10 \| 2.90E+01 \| 9.67E-04 \| 0.02938995 \| 4.50E-01 \| -2.2219479 \| \| HEATR3 \| 7.40E+01 \| 1.23E-03 \| 0.03244251 \| 4.51E-01 \| -2.2196246 \| \| SLC25A6 \| 2.71E+02 \| 4.25E-05 \| 0.01358748 \| 4.51E-01 \| -2.2189701 \| \| AGO4 \| 1.90E+03 \| 1.93E-03 \| 0.03701102 \| 4.51E-01 \| -2.2168414 \| \| RPS13 \| 5.42E+02 \| 4.17E-03 \| 0.0509016 \| 4.51E-01 \| -2.2168078 \| \| NBPF26 \| 4.99E+02 \| 5.92E-05 \| 0.01358748 \| 4.51E-01 \| -2.2154422 \| \| RPL10 \| 1.62E+03 \| 3.00E-03 \| 0.04383474 \| 4.52E-01 \| -2.2110331 \| \| ATP5F1B \| 7.85E+02 \| 2.08E-03 \| 0.03820965 \| 4.52E-01 \| -2.2105689 \| \| SDHAP2 \| 8.87E+01 \| 4.48E-04 \| 0.02201345 \| 4.53E-01 \| -2.2071092 \| \| INPP5K \| 1.94E+02 \| 1.22E-03 \| 0.03244251 \| 4.54E-01 \| -2.2048291 \| \| LOC100287467 \| 7.59E+00 \| 4.78E-03 \| 0.05307894 \| 4.54E-01 \| -2.204398 \| \| RFX2 \| 7.63E+01 \| 1.65E-03 \| 0.03534598 \| 4.54E-01 \| -2.2042939 \| \| SNX29P2 \| 1.79E+01 \| 2.54E-04 \| 0.01800472 \| 4.54E-01 \| -2.2027276 \| \| GLCE \| 1.05E+02 \| 1.46E-03 \| 0.03434026 \| 4.54E-01 \| -2.2024999 \| \| NUP88 \| 1.40E+02 \| 3.04E-03 \| 0.04404095 \| 4.54E-01 \| -2.2020236 \| \| TSPO \| 9.65E+01 \| 1.64E-03 \| 0.03532746 \| 4.55E-01 \| -2.1998232 \| \| LINC01347 \| 1.21E+02 \| 1.39E-03 \| 0.03422635 \| 4.55E-01 \| -2.1997214 \| \| RNF146 \| 3.30E+02 \| 4.76E-03 \| 0.05307894 \| 4.55E-01 \| -2.196915 \| \| RASSF4 \| 8.36E+01 \| 1.39E-03 \| 0.03422635 \| 4.55E-01 \| -2.1963219 \| \| TRPM6 \| 2.84E+02 \| 1.75E-04 \| 0.01672332 \| 4.56E-01 \| -2.1921216 \| \| WDR19 \| 4.20E+01 \| 4.50E-03 \| 0.05227731 \| 4.57E-01 \| -2.1889608 \| \| CHP1 \| 8.04E+02 \| 1.76E-03 \| 0.03573192 \| 4.57E-01 \| -2.1887476 \| \| SLC31A1 \| 1.38E+02 \| 9.13E-04 \| 0.02864918 \| 4.57E-01 \| -2.188393 \| \| F5 \| 1.56E+03 \| 4.25E-03 \| 0.05124371 \| 4.57E-01 \| -2.1865382 \| \| NDUFA11 \| 1.57E+01 \| 4.62E-03 \| 0.05256331 \| 4.59E-01 \| -2.1808739 \| \| GALNT1 \| 4.25E+02 \| 1.67E-03 \| 0.03534598 \| 4.59E-01 \| -2.1766365 \| \| PTAFR \| 9.41E+02 \| 2.83E-04 \| 0.0184668 \| 4.61E-01 \| -2.1700133 \| \| ERGIC3 \| 1.47E+02 \| 2.10E-03 \| 0.03828352 \| 4.61E-01 \| -2.1673644 \| \| ADGRE1 \| 2.48E+02 \| 1.13E-03 \| 0.0310461 \| 4.62E-01 \| -2.1668041 \| \| STK11IP \| 3.42E+01 \| 2.74E-03 \| 0.0429721 \| 4.62E-01 \| -2.1657153 \| \| SFT2D2 \| 1.09E+03 \| 1.05E-03 \| 0.03041505 \| 4.62E-01 \| -2.1629658 \| \| PARVG \| 2.68E+02 \| 3.69E-04 \| 0.02063449 \| 4.63E-01 \| -2.1599725 \| \| MIR22HG \| 5.12E+01 \| 4.27E-03 \| 0.05129215 \| 4.63E-01 \| -2.1591824 \| \| SMPD2 \| 1.63E+01 \| 3.70E-03 \| 0.04772117 \| 4.63E-01 \| -2.1589781 \| \| PCSK5 \| 6.31E+01 \| 1.13E-03 \| 0.0310461 \| 4.63E-01 \| -2.1586355 \| \| GPR155 \| 3.51E+02 \| 1.21E-03 \| 0.03218986 \| 4.63E-01 \| -2.1586106 \| \| GLMP \| 3.35E+01 \| 2.69E-03 \| 0.04284522 \| 4.64E-01 \| -2.1566984 \| \| SIGLEC10 \| 2.21E+02 \| 2.48E-03 \| 0.0412902 \| 4.64E-01 \| -2.1538965 \| \| PEX11B \| 5.50E+01 \| 1.01E-03 \| 0.03013087 \| 4.65E-01 \| -2.1527601 \| \| ZNF783 \| 2.13E+01 \| 6.67E-04 \| 0.02505425 \| 4.65E-01 \| -2.1508256 \| \| CD52 \| 1.01E+02 \| 4.51E-03 \| 0.05227731 \| 4.65E-01 \| -2.1495472 \| \| PARL \| 7.91E+01 \| 3.84E-04 \| 0.02090486 \| 4.66E-01 \| -2.147976 \| \| GTF2IP4 \| 6.92E+02 \| 7.97E-04 \| 0.02766819 \| 4.66E-01 \| -2.1467114 \| \| GTF2IP1 \| 7.47E+02 \| 8.17E-04 \| 0.02771433 \| 4.66E-01 \| -2.1444431 \| \| MARCHF1 \| 1.02E+03 \| 8.79E-04 \| 0.02862553 \| 4.67E-01 \| -2.1402115 \| \| RPL7 \| 5.94E+02 \| 2.14E-03 \| 0.03864 \| 4.67E-01 \| -2.1399517 \| \| RIPOR1 \| 1.16E+02 \| 4.80E-03 \| 0.05307894 \| 4.67E-01 \| -2.1391097 \| \| TCAF2 \| 7.82E+01 \| 4.96E-05 \| 0.01358748 \| 4.68E-01 \| -2.1381151 \| \| WDR91 \| 5.82E+01 \| 2.59E-04 \| 0.01801198 \| 4.68E-01 \| -2.1362371 \| \| HLA-DRA \| 1.61E+03 \| 3.26E-03 \| 0.04541638 \| 4.68E-01 \| -2.1348695 \| \| DHRS4-AS1 \| 3.43E+01 \| 9.72E-04 \| 0.02944172 \| 4.69E-01 \| -2.1321679 \| \| ZDHHC20 \| 1.60E+03 \| 2.85E-03 \| 0.0431483 \| 4.69E-01 \| -2.1316761 \| \| SPG21 \| 3.99E+02 \| 2.20E-03 \| 0.03926859 \| 4.70E-01 \| -2.1287354 \| \| CD300C \| 5.51E+01 \| 1.94E-04 \| 0.01723175 \| 4.70E-01 \| -2.1276053 \| \| COPG2 \| 3.16E+01 \| 4.17E-03 \| 0.0509016 \| 4.70E-01 \| -2.125797 \| \| KCTD6 \| 3.55E+01 \| 6.24E-04 \| 0.02418866 \| 4.71E-01 \| -2.1248264 \| \| KLHDC3 \| 9.60E+01 \| 3.09E-03 \| 0.0441959 \| 4.71E-01 \| -2.1247114 \| \| C12orf49 \| 1.74E+02 \| 2.28E-03 \| 0.03979599 \| 4.72E-01 \| -2.1206771 \| \| FKBP9 \| 3.61E+01 \| 2.71E-03 \| 0.04291402 \| 4.72E-01 \| -2.1198866 \| \| LDLRAD4 \| 1.46E+02 \| 1.17E-04 \| 0.01508103 \| 4.72E-01 \| -2.1185154 \| \| ANXA2P2 \| 2.64E+02 \| 3.62E-03 \| 0.04740123 \| 4.72E-01 \| -2.1179622 \| \| BRI3BP \| 1.56E+02 \| 7.92E-04 \| 0.02766819 \| 4.72E-01 \| -2.1176839 \| \| HSPBAP1 \| 1.41E+02 \| 2.33E-03 \| 0.04011618 \| 4.73E-01 \| -2.1149648 \| \| NPL \| 6.44E+02 \| 2.11E-03 \| 0.0382945 \| 4.73E-01 \| -2.113095 \| \| SMG1P5 \| 1.68E+02 \| 3.00E-03 \| 0.04389068 \| 4.74E-01 \| -2.1119125 \| \| TYK2 \| 4.48E+02 \| 2.90E-03 \| 0.04340126 \| 4.74E-01 \| -2.1119061 \| \| HAVCR2 \| 9.29E+01 \| 1.33E-03 \| 0.03361727 \| 4.74E-01 \| -2.1118924 \| \| OAZ2 \| 3.69E+02 \| 1.06E-03 \| 0.03041505 \| 4.74E-01 \| -2.1117643 \| \| SPSB3 \| 8.90E+01 \| 1.29E-03 \| 0.03293681 \| 4.74E-01 \| -2.1108408 \| \| ANKRD42 \| 3.19E+01 \| 4.90E-04 \| 0.0222881 \| 4.74E-01 \| -2.1101016 \| \| ZDHHC7 \| 3.82E+02 \| 1.12E-04 \| 0.01508103 \| 4.75E-01 \| -2.106137 \| \| TAS2R20 \| 8.95E+00 \| 2.82E-03 \| 0.0431483 \| 4.75E-01 \| -2.1057479 \| \| LILRB2 \| 5.49E+02 \| 1.70E-03 \| 0.03554527 \| 4.75E-01 \| -2.1048733 \| \| TBC1D14 \| 1.17E+03 \| 3.59E-03 \| 0.04729405 \| 4.76E-01 \| -2.10106 \| \| POLR2B \| 4.87E+02 \| 1.91E-03 \| 0.03697811 \| 4.76E-01 \| -2.1007766 \| \| MXD4 \| 6.58E+01 \| 4.65E-03 \| 0.05270169 \| 4.76E-01 \| -2.0987003 \| \| CIPC \| 6.99E+01 \| 6.78E-04 \| 0.02505425 \| 4.77E-01 \| -2.0954645 \| \| ENTR1 \| 1.96E+01 \| 1.13E-03 \| 0.0310461 \| 4.77E-01 \| -2.0949852 \| \| ATP6V1C1 \| 6.13E+02 \| 3.82E-03 \| 0.04859009 \| 4.78E-01 \| -2.0940012 \| \| HACD2 \| 1.63E+02 \| 1.73E-03 \| 0.03557815 \| 4.78E-01 \| -2.0920645 \| \| NR6A1 \| 4.78E+01 \| 1.50E-03 \| 0.03434026 \| 4.78E-01 \| -2.0901443 \| \| ANAPC15 \| 2.27E+01 \| 4.35E-03 \| 0.0518734 \| 4.79E-01 \| -2.0885111 \| \| SIDT2 \| 1.57E+02 \| 2.63E-05 \| 0.01358748 \| 4.79E-01 \| -2.0882677 \| \| OGT \| 1.17E+03 \| 8.78E-04 \| 0.02862553 \| 4.79E-01 \| -2.0879579 \| \| DPYD \| 2.11E+03 \| 4.07E-04 \| 0.02122812 \| 4.79E-01 \| -2.0865027 \| \| TRUB1 \| 4.78E+01 \| 1.27E-03 \| 0.03293681 \| 4.79E-01 \| -2.0855179 \| \| WDR35 \| 4.26E+01 \| 9.51E-05 \| 0.01482445 \| 4.80E-01 \| -2.0847492 \| \| LY86 \| 6.42E+01 \| 2.36E-04 \| 0.01796904 \| 4.80E-01 \| -2.0837087 \| \| DSE \| 5.25E+02 \| 8.52E-04 \| 0.02834622 \| 4.80E-01 \| -2.0816608 \| \| C20orf194 \| 5.56E+01 \| 3.39E-03 \| 0.0462197 \| 4.81E-01 \| -2.0801001 \| \| CHN2 \| 4.54E+01 \| 2.86E-03 \| 0.0431483 \| 4.81E-01 \| -2.0797464 \| \| GALT \| 1.64E+01 \| 1.73E-03 \| 0.03557815 \| 4.81E-01 \| -2.079418 \| \| WDR37 \| 2.96E+02 \| 9.20E-04 \| 0.02879243 \| 4.81E-01 \| -2.0790618 \| \| EIF2S3B \| 2.90E+02 \| 1.97E-03 \| 0.03723394 \| 4.81E-01 \| -2.0775967 \| \| CCNY \| 6.06E+02 \| 9.90E-04 \| 0.02985612 \| 4.81E-01 \| -2.0774452 \| \| SLC4A8 \| 1.30E+01 \| 8.90E-04 \| 0.02862553 \| 4.82E-01 \| -2.0765262 \| \| HDAC9 \| 1.58E+02 \| 1.27E-03 \| 0.03293681 \| 4.82E-01 \| -2.0754607 \| \| TPCN1 \| 6.39E+01 \| 2.14E-04 \| 0.01742995 \| 4.82E-01 \| -2.0730713 \| \| FAM114A1 \| 2.22E+01 \| 3.19E-03 \| 0.04504367 \| 4.83E-01 \| -2.071826 \| \| OXA1L \| 1.69E+02 \| 1.22E-04 \| 0.01508103 \| 4.83E-01 \| -2.0697293 \| \| CRTAP \| 2.36E+02 \| 2.73E-04 \| 0.01838854 \| 4.83E-01 \| -2.0689807 \| \| PANK3 \| 8.67E+02 \| 2.70E-03 \| 0.04291402 \| 4.83E-01 \| -2.0683013 \| \| SMARCD3 \| 9.67E+01 \| 3.31E-04 \| 0.01980958 \| 4.84E-01 \| -2.0673108 \| \| CIAO3 \| 1.08E+01 \| 1.83E-03 \| 0.03598003 \| 4.85E-01 \| -2.0633834 \| \| RPS20 \| 1.70E+03 \| 3.26E-03 \| 0.04541638 \| 4.85E-01 \| -2.0631329 \| \| TOMM20 \| 2.33E+02 \| 1.66E-03 \| 0.03534598 \| 4.85E-01 \| -2.0624305 \| \| DDX39B \| 2.74E+02 \| 2.89E-03 \| 0.04336843 \| 4.85E-01 \| -2.0617884 \| \| SEC61A2 \| 2.10E+01 \| 6.44E-04 \| 0.02457212 \| 4.85E-01 \| -2.0613402 \| \| TAPT1-AS1 \| 1.84E+01 \| 8.36E-04 \| 0.02796205 \| 4.86E-01 \| -2.0592191 \| \| LFNG \| 7.50E+01 \| 1.65E-04 \| 0.01638822 \| 4.86E-01 \| -2.0579777 \| \| GPRIN3 \| 4.08E+02 \| 3.35E-04 \| 0.01980958 \| 4.86E-01 \| -2.0573363 \| \| RPL5 \| 5.37E+02 \| 1.18E-03 \| 0.03173681 \| 4.86E-01 \| -2.0560457 \| \| AKT1S1 \| 3.95E+01 \| 1.67E-03 \| 0.03534598 \| 4.87E-01 \| -2.0537175 \| \| MS4A6A \| 7.82E+02 \| 4.39E-03 \| 0.05197104 \| 4.87E-01 \| -2.0532208 \| \| ARHGAP24 \| 1.55E+02 \| 2.80E-03 \| 0.0431483 \| 4.88E-01 \| -2.0503599 \| \| P2RX4 \| 3.90E+01 \| 1.57E-03 \| 0.03496769 \| 4.88E-01 \| -2.0473186 \| \| ICAM3 \| 8.48E+02 \| 6.37E-04 \| 0.0244386 \| 4.89E-01 \| -2.046913 \| \| CDC123 \| 2.20E+02 \| 5.77E-04 \| 0.02315187 \| 4.89E-01 \| -2.0464676 \| \| CYREN \| 6.90E+01 \| 1.63E-03 \| 0.03522877 \| 4.89E-01 \| -2.0434297 \| \| C5orf24 \| 1.52E+02 \| 4.62E-03 \| 0.05256331 \| 4.90E-01 \| -2.0428641 \| \| VAV2 \| 3.32E+01 \| 2.95E-03 \| 0.04360912 \| 4.90E-01 \| -2.0425592 \| \| NIPAL2 \| 7.03E+01 \| 8.92E-04 \| 0.02862553 \| 4.90E-01 \| -2.0423897 \| \| ATP9B \| 1.64E+02 \| 7.32E-04 \| 0.02626705 \| 4.90E-01 \| -2.0401719 \| \| ULK4 \| 7.38E+01 \| 1.92E-03 \| 0.03697811 \| 4.91E-01 \| -2.0378788 \| \| CYFIP1 \| 2.04E+02 \| 2.38E-04 \| 0.01796904 \| 4.91E-01 \| -2.0369549 \| \| HMOX1 \| 9.84E+01 \| 5.52E-05 \| 0.01358748 \| 4.91E-01 \| -2.0360885 \| \| ANGEL1 \| 3.59E+01 \| 6.42E-05 \| 0.01358748 \| 4.91E-01 \| -2.0353783 \| \| TMTC4 \| 1.99E+01 \| 2.68E-03 \| 0.04281263 \| 4.91E-01 \| -2.0346892 \| \| NLN \| 6.88E+01 \| 1.22E-04 \| 0.01508103 \| 4.92E-01 \| -2.0343448 \| \| RALGPS1 \| 3.71E+01 \| 7.67E-04 \| 0.02690367 \| 4.92E-01 \| -2.0330998 \| \| DMAC1 \| 2.48E+01 \| 3.72E-03 \| 0.04789609 \| 4.92E-01 \| -2.0328914 \| \| GOLGA8B \| 4.74E+01 \| 3.94E-03 \| 0.04944595 \| 4.93E-01 \| -2.0302609 \| \| CYBB \| 6.02E+03 \| 3.36E-03 \| 0.0462197 \| 4.93E-01 \| -2.0294974 \| \| GRK3 \| 4.27E+02 \| 1.32E-05 \| 0.01073864 \| 4.93E-01 \| -2.0293517 \| \| ACTR1B \| 3.68E+01 \| 3.19E-03 \| 0.04504367 \| 4.93E-01 \| -2.0282802 \| \| SLC39A13 \| 1.15E+01 \| 1.62E-03 \| 0.03512358 \| 4.93E-01 \| -2.0269754 \| \| NPHP3 \| 1.30E+02 \| 3.42E-03 \| 0.04632133 \| 4.94E-01 \| -2.0246998 \| \| ZNF548 \| 5.04E+01 \| 8.72E-04 \| 0.02862553 \| 4.94E-01 \| -2.0245435 \| \| MTCH1 \| 2.03E+02 \| 1.58E-03 \| 0.03496769 \| 4.94E-01 \| -2.022572 \| \| GAA \| 1.59E+02 \| 7.28E-05 \| 0.01382889 \| 4.95E-01 \| -2.0208533 \| \| TTC12 \| 1.81E+01 \| 1.04E-03 \| 0.03034051 \| 4.95E-01 \| -2.0204405 \| \| MMP14 \| 1.10E+01 \| 4.51E-03 \| 0.05227731 \| 4.95E-01 \| -2.019389 \| \| UNC119 \| 9.12E+01 \| 3.61E-04 \| 0.02063449 \| 4.95E-01 \| -2.0189377 \| \| FCN1 \| 3.22E+03 \| 1.86E-04 \| 0.01718472 \| 4.96E-01 \| -2.0175554 \| \| HLA-DMB \| 9.83E+01 \| 4.63E-03 \| 0.0525919 \| 4.96E-01 \| -2.0173009 \| \| FASTK \| 2.65E+01 \| 4.99E-03 \| 0.0536727 \| 4.96E-01 \| -2.0168227 \| \| MCUB \| 1.29E+02 \| 3.69E-04 \| 0.02063449 \| 4.96E-01 \| -2.0152927 \| \| NACC2 \| 3.19E+02 \| 5.11E-03 \| 0.05426777 \| 4.97E-01 \| -2.0112616 \| \| AIF1 \| 3.19E+02 \| 6.17E-04 \| 0.02401783 \| 4.97E-01 \| -2.0110471 \| \| NPRL2 \| 2.85E+01 \| 1.70E-03 \| 0.03554527 \| 4.98E-01 \| -2.0097477 \| \| CTSO \| 1.01E+02 \| 6.11E-05 \| 0.01358748 \| 4.98E-01 \| -2.0087184 \| \| OPA1 \| 5.51E+02 \| 4.08E-03 \| 0.05046684 \| 4.99E-01 \| -2.0054995 \| \| NUP85 \| 4.71E+01 \| 1.48E-03 \| 0.03434026 \| 4.99E-01 \| -2.0038092 \| \| ARSG \| 2.07E+02 \| 8.95E-04 \| 0.02862553 \| 4.99E-01 \| -2.0020345 \| \| KIAA0355 \| 9.43E+01 \| 6.79E-05 \| 0.01358748 \| 5.00E-01 \| -2.0013188 \| \| LINC00963 \| 6.14E+01 \| 2.80E-03 \| 0.0431483 \| 5.00E-01 \| -2.0003264 \| \| STAT5B \| 1.98E+03 \| 1.82E-03 \| 0.03592144 \| 5.00E-01 \| -1.9999869 \| \| DPYSL2 \| 5.83E+02 \| 7.56E-05 \| 0.01383902 \| 5.01E-01 \| -1.9969506 \| \| PITPNM1 \| 1.03E+02 \| 4.16E-03 \| 0.05088729 \| 5.01E-01 \| -1.9964981 \| \| MTPAP \| 7.98E+01 \| 1.14E-04 \| 0.01508103 \| 5.01E-01 \| -1.996249 \| \| ZBTB14 \| 1.08E+02 \| 5.43E-04 \| 0.02271088 \| 5.01E-01 \| -1.9958284 \| \| SYNGR2 \| 1.25E+02 \| 1.06E-04 \| 0.01508103 \| 5.01E-01 \| -1.9944314 \| \| WDR73 \| 3.80E+01 \| 8.00E-04 \| 0.02766819 \| 5.01E-01 \| -1.9943069 \| \| PLXNB2 \| 2.75E+02 \| 1.08E-03 \| 0.03063671 \| 5.02E-01 \| -1.9928811 \| \| RPL18 \| 7.50E+02 \| 4.19E-03 \| 0.05097746 \| 5.02E-01 \| -1.991696 \| \| ECHDC1 \| 1.55E+02 \| 5.02E-04 \| 0.02238866 \| 5.02E-01 \| -1.9913032 \| \| RACK1 \| 8.19E+02 \| 3.25E-03 \| 0.04541638 \| 5.03E-01 \| -1.9891474 \| \| GLUD2 \| 7.01E+01 \| 3.33E-03 \| 0.04587749 \| 5.03E-01 \| -1.9889927 \| \| AP1AR \| 5.05E+01 \| 5.11E-04 \| 0.02246294 \| 5.03E-01 \| -1.9875696 \| \| EEF1G \| 1.03E+03 \| 1.91E-03 \| 0.03697811 \| 5.03E-01 \| -1.9864204 \| \| SERINC5 \| 3.30E+02 \| 3.06E-03 \| 0.04405569 \| 5.04E-01 \| -1.9855611 \| \| C16orf70 \| 8.75E+01 \| 4.61E-04 \| 0.02201345 \| 5.04E-01 \| -1.9849748 \| \| SCIMP \| 1.66E+02 \| 2.66E-03 \| 0.04273147 \| 5.04E-01 \| -1.9844904 \| \| CLK2 \| 6.62E+01 \| 1.43E-03 \| 0.03422635 \| 5.04E-01 \| -1.9838845 \| \| KPNA1 \| 6.64E+02 \| 2.20E-03 \| 0.03926859 \| 5.04E-01 \| -1.982426 \| \| MAN2C1 \| 5.23E+01 \| 3.62E-03 \| 0.04740123 \| 5.05E-01 \| -1.9816068 \| \| CSDE1 \| 3.31E+03 \| 8.97E-04 \| 0.02862553 \| 5.05E-01 \| -1.9799002 \| \| CAMK2D \| 1.35E+02 \| 3.88E-04 \| 0.02090486 \| 5.05E-01 \| -1.9793459 \| \| GLO1 \| 1.11E+02 \| 1.49E-03 \| 0.03434026 \| 5.05E-01 \| -1.9788088 \| \| DNAJC10 \| 3.82E+02 \| 1.34E-04 \| 0.01562712 \| 5.05E-01 \| -1.9783646 \| \| FGD6 \| 8.98E+01 \| 4.56E-03 \| 0.05231298 \| 5.06E-01 \| -1.9755749 \| \| IMPACT \| 6.08E+01 \| 1.99E-03 \| 0.03739057 \| 5.06E-01 \| -1.9743962 \| \| DGAT1 \| 3.76E+01 \| 4.40E-03 \| 0.0519792 \| 5.07E-01 \| -1.9736258 \| \| GGA2 \| 1.41E+02 \| 8.96E-05 \| 0.01441657 \| 5.07E-01 \| -1.9719728 \| \| ARHGEF40 \| 4.19E+02 \| 4.56E-03 \| 0.05231298 \| 5.07E-01 \| -1.9715631 \| \| PIAS3 \| 3.78E+01 \| 1.02E-03 \| 0.03031049 \| 5.07E-01 \| -1.9710214 \| \| RPL10A \| 3.54E+02 \| 4.49E-03 \| 0.05226789 \| 5.07E-01 \| -1.970493 \| \| ALDH3A2 \| 9.15E+01 \| 4.62E-03 \| 0.05256331 \| 5.08E-01 \| -1.9702861 \| \| SWAP70 \| 2.26E+02 \| 1.56E-04 \| 0.01622824 \| 5.08E-01 \| -1.9682501 \| \| GPAM \| 2.92E+01 \| 1.25E-03 \| 0.03271222 \| 5.08E-01 \| -1.9668464 \| \| ANXA5 \| 6.84E+02 \| 2.52E-04 \| 0.01800472 \| 5.09E-01 \| -1.9631598 \| \| SH3BGRL \| 1.46E+03 \| 1.31E-03 \| 0.03337596 \| 5.10E-01 \| -1.9608044 \| \| NARS1 \| 2.26E+02 \| 2.08E-04 \| 0.01737605 \| 5.10E-01 \| -1.9597054 \| \| SPG7 \| 5.82E+01 \| 2.74E-03 \| 0.0429721 \| 5.10E-01 \| -1.9595828 \| \| WDR74 \| 2.57E+01 \| 3.38E-03 \| 0.0462197 \| 5.11E-01 \| -1.9559376 \| \| FKTN \| 4.13E+01 \| 5.42E-04 \| 0.02271088 \| 5.12E-01 \| -1.9540258 \| \| TIMM17B \| 5.25E+01 \| 4.57E-03 \| 0.05231298 \| 5.12E-01 \| -1.9525916 \| \| SLC22A4 \| 1.88E+02 \| 8.38E-04 \| 0.02796205 \| 5.13E-01 \| -1.9502049 \| \| FAM126A \| 3.34E+02 \| 1.70E-03 \| 0.03554527 \| 5.13E-01 \| -1.9494545 \| \| PRNP \| 2.39E+02 \| 3.60E-03 \| 0.04735767 \| 5.14E-01 \| -1.9449662 \| \| RPS8 \| 1.24E+03 \| 4.86E-03 \| 0.05322635 \| 5.15E-01 \| -1.9435562 \| \| FGFR1OP \| 1.49E+02 \| 4.71E-03 \| 0.05301601 \| 5.15E-01 \| -1.9430578 \| \| LPXN \| 1.66E+02 \| 3.86E-04 \| 0.02090486 \| 5.15E-01 \| -1.9421942 \| \| GLB1 \| 2.19E+02 \| 5.29E-04 \| 0.02266365 \| 5.15E-01 \| -1.9410567 \| \| ASB1 \| 8.18E+01 \| 6.84E-04 \| 0.0250927 \| 5.15E-01 \| -1.9407109 \| \| PECAM1 \| 9.48E+02 \| 1.99E-04 \| 0.01723618 \| 5.15E-01 \| -1.9399227 \| \| PNPLA8 \| 2.87E+02 \| 3.68E-04 \| 0.02063449 \| 5.16E-01 \| -1.9373162 \| \| QARS1 \| 1.26E+02 \| 8.96E-04 \| 0.02862553 \| 5.17E-01 \| -1.9353533 \| \| NEU3 \| 5.86E+01 \| 3.00E-04 \| 0.01905573 \| 5.17E-01 \| -1.9343836 \| \| P4HA1 \| 1.55E+02 \| 1.09E-03 \| 0.03064526 \| 5.17E-01 \| -1.933212 \| \| MAP10 \| 2.13E+01 \| 3.17E-03 \| 0.04494434 \| 5.18E-01 \| -1.9323628 \| \| UBTD2 \| 4.83E+01 \| 4.25E-03 \| 0.05124371 \| 5.18E-01 \| -1.9320446 \| \| CHM \| 1.31E+02 \| 1.18E-03 \| 0.03173681 \| 5.18E-01 \| -1.9316123 \| \| FOXO1 \| 4.44E+02 \| 2.69E-03 \| 0.04281263 \| 5.18E-01 \| -1.9311192 \| \| LSM10 \| 7.92E+01 \| 2.25E-03 \| 0.0397705 \| 5.18E-01 \| -1.9304514 \| \| THAP9-AS1 \| 3.86E+01 \| 1.74E-03 \| 0.03566312 \| 5.18E-01 \| -1.9300925 \| \| ZSWIM6 \| 5.61E+02 \| 1.15E-04 \| 0.01508103 \| 5.18E-01 \| -1.9299519 \| \| SNX17 \| 2.12E+02 \| 8.08E-04 \| 0.02766819 \| 5.19E-01 \| -1.9285252 \| \| TUBGCP2 \| 1.20E+02 \| 6.48E-04 \| 0.02457212 \| 5.19E-01 \| -1.9280527 \| \| PTDSS1 \| 2.27E+02 \| 1.77E-03 \| 0.03579792 \| 5.19E-01 \| -1.9267585 \| \| COP1 \| 1.03E+03 \| 2.91E-03 \| 0.04344435 \| 5.19E-01 \| -1.9252721 \| \| DGKA \| 1.77E+02 \| 1.40E-05 \| 0.01073864 \| 5.20E-01 \| -1.9244941 \| \| MCRS1 \| 6.25E+01 \| 3.79E-04 \| 0.02090486 \| 5.20E-01 \| -1.9241529 \| \| CSF2RA \| 4.48E+02 \| 1.95E-03 \| 0.0370502 \| 5.20E-01 \| -1.9213173 \| \| CENPBD1P1 \| 8.37E+01 \| 1.96E-04 \| 0.01723175 \| 5.21E-01 \| -1.920648 \| \| ATP2B1 \| 8.04E+02 \| 1.24E-03 \| 0.03266901 \| 5.21E-01 \| -1.9194147 \| \| TBXAS1 \| 9.08E+02 \| 2.02E-03 \| 0.03784359 \| 5.21E-01 \| -1.9187429 \| \| FAM168B \| 2.44E+02 \| 2.45E-03 \| 0.04112079 \| 5.21E-01 \| -1.9186532 \| \| ADAP2 \| 6.73E+01 \| 1.12E-03 \| 0.0310461 \| 5.21E-01 \| -1.9183894 \| \| PARGP1-AGAP4 \| 4.47E+01 \| 5.02E-03 \| 0.05376205 \| 5.21E-01 \| -1.9181242 \| \| SAR1B \| 2.48E+02 \| 4.51E-03 \| 0.05227731 \| 5.22E-01 \| -1.9171706 \| \| RPS9 \| 7.84E+02 \| 1.56E-03 \| 0.03496769 \| 5.22E-01 \| -1.9159262 \| \| SURF1 \| 4.50E+01 \| 4.15E-04 \| 0.02146728 \| 5.22E-01 \| -1.9155815 \| \| RPL13A \| 8.43E+02 \| 1.75E-03 \| 0.03566312 \| 5.22E-01 \| -1.9154439 \| \| DUS3L \| 1.23E+01 \| 4.04E-03 \| 0.05014701 \| 5.22E-01 \| -1.9149511 \| \| SLC25A13 \| 4.09E+01 \| 3.16E-04 \| 0.01931625 \| 5.22E-01 \| -1.9145742 \| \| IARS2 \| 1.90E+02 \| 2.86E-03 \| 0.0431483 \| 5.22E-01 \| -1.9142324 \| \| ZNF266 \| 1.14E+02 \| 4.61E-04 \| 0.02201345 \| 5.23E-01 \| -1.9131841 \| \| ADAM10 \| 2.28E+03 \| 5.04E-03 \| 0.0538745 \| 5.23E-01 \| -1.9121758 \| \| USP9X \| 2.06E+03 \| 1.39E-03 \| 0.03422635 \| 5.24E-01 \| -1.908918 \| \| TALDO1 \| 1.53E+03 \| 2.43E-04 \| 0.01800472 \| 5.25E-01 \| -1.904113 \| \| PCGF3 \| 2.12E+02 \| 3.83E-03 \| 0.04859009 \| 5.25E-01 \| -1.9039938 \| \| STX12 \| 2.94E+02 \| 3.46E-03 \| 0.04657396 \| 5.25E-01 \| -1.9032548 \| \| AHCYL1 \| 3.09E+02 \| 1.20E-04 \| 0.01508103 \| 5.26E-01 \| -1.9021537 \| \| SMIM7 \| 8.30E+01 \| 2.24E-03 \| 0.03974504 \| 5.26E-01 \| -1.9018684 \| \| PTRH2 \| 3.79E+01 \| 3.14E-04 \| 0.01931625 \| 5.26E-01 \| -1.901684 \| \| ATF6 \| 1.03E+03 \| 8.04E-04 \| 0.02766819 \| 5.26E-01 \| -1.9013276 \| \| GLUD1 \| 2.82E+02 \| 2.08E-03 \| 0.03821496 \| 5.26E-01 \| -1.9004306 \| \| MYO9A \| 2.16E+02 \| 8.55E-05 \| 0.01407615 \| 5.26E-01 \| -1.8996317 \| \| RRAGA \| 1.40E+02 \| 4.87E-03 \| 0.05322635 \| 5.27E-01 \| -1.8992343 \| \| DTWD2 \| 3.56E+01 \| 1.50E-03 \| 0.03434026 \| 5.27E-01 \| -1.8988561 \| \| ARL6 \| 1.01E+01 \| 4.70E-03 \| 0.05301601 \| 5.27E-01 \| -1.8985356 \| \| HCK \| 1.23E+03 \| 1.28E-03 \| 0.03293681 \| 5.27E-01 \| -1.8975156 \| \| TOMM22 \| 9.64E+01 \| 2.04E-03 \| 0.03804503 \| 5.27E-01 \| -1.8972349 \| \| PARP15 \| 6.31E+01 \| 4.96E-03 \| 0.05353559 \| 5.27E-01 \| -1.8962122 \| \| DDHD2 \| 9.28E+01 \| 1.42E-04 \| 0.0157205 \| 5.28E-01 \| -1.8951953 \| \| SLC35E2B \| 2.85E+02 \| 5.02E-05 \| 0.01358748 \| 5.28E-01 \| -1.8935415 \| \| ARFGAP3 \| 2.69E+02 \| 1.44E-03 \| 0.03422635 \| 5.28E-01 \| -1.8927478 \| \| KIAA0825 \| 5.06E+02 \| 2.91E-03 \| 0.04344435 \| 5.28E-01 \| -1.8923905 \| \| SF3B1 \| 2.65E+03 \| 7.46E-04 \| 0.02646243 \| 5.29E-01 \| -1.889562 \| \| CLN8 \| 4.11E+01 \| 5.81E-05 \| 0.01358748 \| 5.30E-01 \| -1.8871474 \| \| PACC1 \| 3.17E+01 \| 7.53E-04 \| 0.02649127 \| 5.30E-01 \| -1.8862795 \| \| TRIM66 \| 4.12E+01 \| 1.49E-03 \| 0.03434026 \| 5.31E-01 \| -1.883628 \| \| POGLUT1 \| 6.53E+01 \| 5.01E-03 \| 0.05376205 \| 5.31E-01 \| -1.8820426 \| \| ABCC5 \| 1.85E+02 \| 4.44E-03 \| 0.05222976 \| 5.31E-01 \| -1.8816606 \| \| DENND11 \| 1.48E+02 \| 3.06E-04 \| 0.01914308 \| 5.31E-01 \| -1.8815171 \| \| DHX57 \| 1.01E+02 \| 2.66E-04 \| 0.01810876 \| 5.32E-01 \| -1.8796791 \| \| CDC16 \| 1.48E+02 \| 3.39E-04 \| 0.01980958 \| 5.32E-01 \| -1.8789905 \| \| LOC645513 \| 5.77E+01 \| 6.16E-04 \| 0.02401783 \| 5.33E-01 \| -1.87709 \| \| SIAH1 \| 1.26E+02 \| 3.58E-03 \| 0.04729405 \| 5.33E-01 \| -1.8769736 \| \| DICER1 \| 2.63E+03 \| 1.47E-03 \| 0.03434026 \| 5.33E-01 \| -1.876966 \| \| ANXA2 \| 5.10E+02 \| 2.40E-03 \| 0.04075716 \| 5.33E-01 \| -1.876042 \| \| KAT8 \| 6.85E+01 \| 1.06E-03 \| 0.03041505 \| 5.33E-01 \| -1.8753946 \| \| TMEM161B \| 5.56E+01 \| 3.81E-04 \| 0.02090486 \| 5.33E-01 \| -1.8753883 \| \| TRIT1 \| 2.60E+01 \| 1.31E-03 \| 0.03338401 \| 5.34E-01 \| -1.8728391 \| \| LOC285074 \| 1.57E+02 \| 1.14E-03 \| 0.0310461 \| 5.34E-01 \| -1.8721096 \| \| CYB561A3 \| 7.08E+01 \| 1.41E-03 \| 0.03422635 \| 5.35E-01 \| -1.8708018 \| \| C6orf120 \| 2.28E+02 \| 2.95E-03 \| 0.04360912 \| 5.35E-01 \| -1.8693914 \| \| TRIAP1 \| 1.80E+01 \| 5.11E-03 \| 0.05427537 \| 5.35E-01 \| -1.868308 \| \| ATG2B \| 5.17E+02 \| 2.48E-04 \| 0.01800472 \| 5.35E-01 \| -1.8679801 \| \| ACAA2 \| 7.99E+01 \| 1.62E-03 \| 0.03521622 \| 5.36E-01 \| -1.8665293 \| \| ATPAF2 \| 2.03E+01 \| 3.16E-03 \| 0.04493488 \| 5.36E-01 \| -1.8664392 \| \| DUSP22 \| 3.05E+02 \| 9.06E-04 \| 0.02864918 \| 5.36E-01 \| -1.8662519 \| \| AP3S2 \| 1.69E+02 \| 7.03E-05 \| 0.01361826 \| 5.36E-01 \| -1.8660547 \| \| DET1 \| 2.03E+01 \| 2.63E-03 \| 0.04255273 \| 5.36E-01 \| -1.8658718 \| \| NDST2 \| 2.00E+02 \| 4.60E-03 \| 0.05256331 \| 5.36E-01 \| -1.8650259 \| \| PPP3CB \| 4.95E+02 \| 1.04E-03 \| 0.03034051 \| 5.37E-01 \| -1.8633529 \| \| ATP6V1F \| 1.10E+02 \| 2.37E-03 \| 0.04037057 \| 5.37E-01 \| -1.8632929 \| \| RNGTT \| 2.24E+02 \| 2.78E-04 \| 0.0184668 \| 5.37E-01 \| -1.8626054 \| \| AGO2 \| 1.26E+03 \| 3.45E-03 \| 0.04653622 \| 5.39E-01 \| -1.8569318 \| \| NANS \| 5.80E+01 \| 3.42E-03 \| 0.04632133 \| 5.39E-01 \| -1.8569205 \| \| DCAF8 \| 3.17E+02 \| 2.53E-04 \| 0.01800472 \| 5.39E-01 \| -1.856748 \| \| STX16 \| 6.69E+02 \| 1.44E-03 \| 0.03422635 \| 5.39E-01 \| -1.8562349 \| \| NRDC \| 1.81E+03 \| 6.08E-04 \| 0.02393371 \| 5.39E-01 \| -1.8557894 \| \| SERTAD2 \| 3.04E+02 \| 2.97E-03 \| 0.0437064 \| 5.39E-01 \| -1.855566 \| \| RPL22 \| 3.17E+02 \| 1.71E-04 \| 0.01655051 \| 5.40E-01 \| -1.8526897 \| \| ZNF573 \| 1.55E+01 \| 2.73E-03 \| 0.0429721 \| 5.40E-01 \| -1.8518104 \| \| VCPKMT \| 8.53E+01 \| 2.24E-04 \| 0.01793379 \| 5.41E-01 \| -1.8477445 \| \| DCUN1D2 \| 2.81E+01 \| 1.98E-03 \| 0.03723394 \| 5.41E-01 \| -1.8471987 \| \| HGSNAT \| 1.69E+02 \| 2.06E-03 \| 0.03807473 \| 5.41E-01 \| -1.8471362 \| \| SIRPB2 \| 3.49E+02 \| 4.76E-04 \| 0.02227986 \| 5.41E-01 \| -1.8471001 \| \| VPS8 \| 9.16E+02 \| 1.13E-03 \| 0.0310461 \| 5.42E-01 \| -1.8463262 \| \| ERCC3 \| 7.87E+01 \| 2.41E-03 \| 0.04082713 \| 5.42E-01 \| -1.8458027 \| \| DMXL1 \| 7.77E+02 \| 4.59E-04 \| 0.02201345 \| 5.42E-01 \| -1.8445628 \| \| CLCN6 \| 3.32E+01 \| 4.46E-03 \| 0.05222976 \| 5.42E-01 \| -1.8434209 \| \| THOC5 \| 2.65E+02 \| 4.16E-03 \| 0.05088729 \| 5.43E-01 \| -1.8428136 \| \| SLC25A43 \| 3.94E+01 \| 4.81E-03 \| 0.05307894 \| 5.43E-01 \| -1.8427399 \| \| NPEPPS \| 8.42E+02 \| 6.18E-04 \| 0.02401783 \| 5.43E-01 \| -1.8425031 \| \| UBE2E2 \| 5.48E+01 \| 2.41E-03 \| 0.04082713 \| 5.43E-01 \| -1.8414588 \| \| COMMD10 \| 7.42E+01 \| 9.68E-04 \| 0.02938995 \| 5.43E-01 \| -1.8404021 \| \| NEURL4 \| 3.48E+01 \| 3.66E-03 \| 0.04756982 \| 5.43E-01 \| -1.8403178 \| \| TASOR \| 1.07E+03 \| 1.72E-03 \| 0.03557815 \| 5.44E-01 \| -1.839393 \| \| ZHX3 \| 2.67E+01 \| 1.74E-03 \| 0.03566312 \| 5.44E-01 \| -1.8386332 \| \| GPATCH2 \| 6.34E+01 \| 2.55E-04 \| 0.01800472 \| 5.44E-01 \| -1.8368 \| \| HOOK3 \| 8.81E+02 \| 1.58E-03 \| 0.03496769 \| 5.45E-01 \| -1.8362842 \| \| EIF2S3 \| 8.36E+02 \| 3.92E-03 \| 0.04937362 \| 5.45E-01 \| -1.8352261 \| \| FLOT2 \| 2.04E+03 \| 3.86E-03 \| 0.04888126 \| 5.45E-01 \| -1.8350902 \| \| MROH1 \| 3.30E+01 \| 4.80E-03 \| 0.05307894 \| 5.45E-01 \| -1.8345873 \| \| TUBGCP3 \| 2.49E+02 \| 1.09E-03 \| 0.03064526 \| 5.45E-01 \| -1.8345288 \| \| CRYBG3 \| 2.14E+02 \| 7.11E-04 \| 0.02561567 \| 5.45E-01 \| -1.8336643 \| \| TTYH2 \| 2.38E+01 \| 3.03E-03 \| 0.04404095 \| 5.45E-01 \| -1.8333757 \| \| CHRAC1 \| 6.42E+01 \| 2.53E-04 \| 0.01800472 \| 5.46E-01 \| -1.8304669 \| \| COPZ1 \| 2.28E+02 \| 3.14E-03 \| 0.04486926 \| 5.46E-01 \| -1.8303677 \| \| TOR2A \| 2.16E+01 \| 4.09E-03 \| 0.05046684 \| 5.46E-01 \| -1.8303346 \| \| MTAP \| 8.10E+01 \| 1.15E-04 \| 0.01508103 \| 5.47E-01 \| -1.8284528 \| \| IGF1R \| 1.16E+03 \| 3.25E-03 \| 0.04541638 \| 5.47E-01 \| -1.8281329 \| \| EIF3K \| 2.19E+02 \| 4.59E-04 \| 0.02201345 \| 5.48E-01 \| -1.8246126 \| \| ARRB2 \| 1.61E+03 \| 2.95E-03 \| 0.04360912 \| 5.48E-01 \| -1.8236656 \| \| UBE4B \| 4.18E+02 \| 6.96E-04 \| 0.02526436 \| 5.48E-01 \| -1.8232733 \| \| VPS35 \| 7.28E+02 \| 4.38E-03 \| 0.05197104 \| 5.49E-01 \| -1.8227365 \| \| RNMT \| 1.35E+02 \| 4.55E-05 \| 0.01358748 \| 5.49E-01 \| -1.8219538 \| \| PPM1M \| 3.22E+02 \| 2.85E-03 \| 0.0431483 \| 5.49E-01 \| -1.821842 \| \| MRPS16 \| 1.07E+02 \| 1.71E-03 \| 0.03557815 \| 5.50E-01 \| -1.8183478 \| \| BRWD3 \| 1.07E+03 \| 4.74E-03 \| 0.05304222 \| 5.50E-01 \| -1.8167705 \| \| WDR24 \| 1.79E+01 \| 4.27E-03 \| 0.05129215 \| 5.51E-01 \| -1.815622 \| \| SLC7A6 \| 1.23E+02 \| 1.36E-04 \| 0.01562712 \| 5.51E-01 \| -1.8149723 \| \| SLC25A36 \| 1.28E+02 \| 4.95E-03 \| 0.05349049 \| 5.51E-01 \| -1.8143972 \| \| F2RL1 \| 2.10E+02 \| 3.21E-03 \| 0.0450618 \| 5.51E-01 \| -1.8140277 \| \| SLC39A10 \| 1.31E+02 \| 6.46E-04 \| 0.02457212 \| 5.51E-01 \| -1.8138317 \| \| GAPVD1 \| 8.88E+02 \| 3.28E-03 \| 0.04553905 \| 5.52E-01 \| -1.812856 \| \| WDR13 \| 7.43E+01 \| 2.33E-03 \| 0.04010637 \| 5.52E-01 \| -1.8126141 \| \| RSRP1 \| 1.34E+03 \| 2.46E-03 \| 0.041125 \| 5.52E-01 \| -1.812605 \| \| ZNF395 \| 7.79E+01 \| 6.77E-04 \| 0.02505425 \| 5.52E-01 \| -1.810245 \| \| AREL1 \| 7.02E+02 \| 3.76E-03 \| 0.04818244 \| 5.52E-01 \| -1.8100701 \| \| BOD1 \| 2.19E+01 \| 2.56E-03 \| 0.04188029 \| 5.53E-01 \| -1.8097943 \| \| ANKZF1 \| 8.82E+01 \| 1.80E-03 \| 0.03592144 \| 5.53E-01 \| -1.8094893 \| \| GSTK1 \| 3.04E+02 \| 1.78E-03 \| 0.035844 \| 5.53E-01 \| -1.8089763 \| \| EFTUD2 \| 2.34E+02 \| 1.37E-04 \| 0.01562712 \| 5.53E-01 \| -1.80873 \| \| WDFY2 \| 3.93E+02 \| 6.85E-04 \| 0.0250927 \| 5.53E-01 \| -1.8081725 \| \| MPDU1 \| 5.30E+01 \| 2.43E-03 \| 0.0409125 \| 5.53E-01 \| -1.8079007 \| \| PANK4 \| 5.65E+01 \| 3.36E-03 \| 0.04613561 \| 5.54E-01 \| -1.8065539 \| \| RPL13AP5 \| 7.08E+02 \| 3.32E-03 \| 0.04584072 \| 5.54E-01 \| -1.8064802 \| \| EIF3E \| 4.56E+02 \| 5.02E-03 \| 0.05376205 \| 5.54E-01 \| -1.8051189 \| \| DCLRE1B \| 4.10E+01 \| 3.39E-03 \| 0.0462197 \| 5.54E-01 \| -1.8049219 \| \| UBE2J2 \| 7.42E+01 \| 2.80E-03 \| 0.0431483 \| 5.54E-01 \| -1.8046744 \| \| DOCK5 \| 3.08E+03 \| 3.68E-03 \| 0.04765103 \| 5.54E-01 \| -1.8039236 \| \| LINC00667 \| 4.43E+01 \| 4.60E-03 \| 0.05256331 \| 5.54E-01 \| -1.8035415 \| \| SMG1P1 \| 9.18E+02 \| 1.12E-03 \| 0.0310461 \| 5.55E-01 \| -1.8017173 \| \| PSKH1 \| 6.27E+01 \| 1.41E-04 \| 0.0156962 \| 5.55E-01 \| -1.8016587 \| \| TRAPPC11 \| 4.02E+02 \| 2.35E-03 \| 0.04019134 \| 5.55E-01 \| -1.8007769 \| \| EIF2B4 \| 2.64E+01 \| 3.87E-03 \| 0.04889995 \| 5.55E-01 \| -1.8005723 \| \| SLC25A3 \| 4.04E+02 \| 4.00E-03 \| 0.04984038 \| 5.56E-01 \| -1.7984855 \| \| SLFN11 \| 3.32E+02 \| 9.42E-04 \| 0.02905449 \| 5.56E-01 \| -1.7978444 \| \| TSC2 \| 1.40E+02 \| 1.83E-03 \| 0.03604209 \| 5.57E-01 \| -1.7965975 \| \| TMEM218 \| 2.85E+01 \| 4.98E-03 \| 0.05366228 \| 5.57E-01 \| -1.796284 \| \| GIMAP6 \| 4.09E+02 \| 2.37E-03 \| 0.04034587 \| 5.57E-01 \| -1.7950903 \| \| CLOCK \| 1.22E+02 \| 3.36E-04 \| 0.01980958 \| 5.57E-01 \| -1.7945661 \| \| USP7 \| 7.56E+02 \| 4.92E-04 \| 0.0222881 \| 5.58E-01 \| -1.7933179 \| \| KIAA0753 \| 7.66E+01 \| 1.69E-03 \| 0.03554527 \| 5.58E-01 \| -1.7930184 \| \| USP45 \| 8.78E+01 \| 4.47E-03 \| 0.05226745 \| 5.58E-01 \| -1.7921991 \| \| EIF3B \| 2.06E+02 \| 2.90E-03 \| 0.04340126 \| 5.58E-01 \| -1.7915326 \| \| ERF \| 5.03E+01 \| 4.41E-03 \| 0.05200437 \| 5.58E-01 \| -1.7911986 \| \| RASGRP4 \| 3.75E+02 \| 2.66E-03 \| 0.04273147 \| 5.59E-01 \| -1.789628 \| \| SIRPA \| 1.70E+03 \| 2.06E-03 \| 0.03807473 \| 5.59E-01 \| -1.7886853 \| \| ITGA4 \| 1.14E+03 \| 2.38E-03 \| 0.04037057 \| 5.59E-01 \| -1.7886842 \| \| ZCCHC7 \| 2.41E+02 \| 7.29E-04 \| 0.02620136 \| 5.60E-01 \| -1.7868041 \| \| PEX5 \| 4.61E+01 \| 3.54E-03 \| 0.04702324 \| 5.60E-01 \| -1.7867661 \| \| QTRT2 \| 9.98E+01 \| 2.08E-04 \| 0.01737605 \| 5.60E-01 \| -1.7851507 \| \| SYNJ2BP \| 5.74E+01 \| 1.63E-03 \| 0.03522877 \| 5.60E-01 \| -1.7848902 \| \| PAN2 \| 5.96E+01 \| 4.65E-03 \| 0.0526976 \| 5.60E-01 \| -1.7848451 \| \| STARD7 \| 3.95E+02 \| 1.94E-03 \| 0.03702835 \| 5.61E-01 \| -1.7836526 \| \| CHCHD7 \| 6.58E+01 \| 3.15E-03 \| 0.04486926 \| 5.61E-01 \| -1.7832864 \| \| RPL17 \| 4.68E+02 \| 2.55E-03 \| 0.04186861 \| 5.61E-01 \| -1.7816433 \| \| SUDS3 \| 3.52E+02 \| 5.70E-04 \| 0.02292566 \| 5.61E-01 \| -1.7812358 \| \| GPAT4 \| 1.61E+02 \| 3.37E-03 \| 0.0462197 \| 5.61E-01 \| -1.7811874 \| \| RNPEP \| 2.39E+02 \| 1.56E-03 \| 0.03496769 \| 5.62E-01 \| -1.7806263 \| \| ZNF689 \| 3.39E+01 \| 3.82E-03 \| 0.04859009 \| 5.62E-01 \| -1.7793111 \| \| CERS5 \| 9.24E+01 \| 1.65E-03 \| 0.03534598 \| 5.62E-01 \| -1.7779914 \| \| CASP2 \| 3.12E+02 \| 3.93E-04 \| 0.02103499 \| 5.63E-01 \| -1.7766854 \| \| FRMD8 \| 1.09E+02 \| 2.17E-03 \| 0.03890483 \| 5.63E-01 \| -1.7764285 \| \| AP2A2 \| 1.08E+02 \| 4.85E-04 \| 0.0222881 \| 5.63E-01 \| -1.7762886 \| \| OTUD1 \| 2.10E+02 \| 9.29E-04 \| 0.02891446 \| 5.64E-01 \| -1.7741552 \| \| CLP1 \| 4.84E+01 \| 1.82E-03 \| 0.03592144 \| 5.64E-01 \| -1.7736502 \| \| CSNK2A3 \| 1.30E+02 \| 2.38E-04 \| 0.01796904 \| 5.64E-01 \| -1.7731475 \| \| TMEM120A \| 5.99E+01 \| 3.94E-03 \| 0.04944595 \| 5.64E-01 \| -1.7731088 \| \| VPS36 \| 2.60E+02 \| 1.73E-03 \| 0.03557815 \| 5.64E-01 \| -1.7726823 \| \| RBL2 \| 1.63E+03 \| 4.40E-03 \| 0.0519792 \| 5.65E-01 \| -1.7712193 \| \| ZNF862 \| 7.90E+01 \| 5.11E-03 \| 0.05426777 \| 5.65E-01 \| -1.7690342 \| \| AHCYL2 \| 5.94E+01 \| 2.27E-03 \| 0.0397705 \| 5.65E-01 \| -1.7689306 \| \| RTL6 \| 4.21E+01 \| 3.28E-03 \| 0.04553905 \| 5.65E-01 \| -1.7686088 \| \| DYRK2 \| 3.02E+02 \| 2.75E-03 \| 0.04297444 \| 5.66E-01 \| -1.7678718 \| \| NBPF25P \| 6.63E+02 \| 6.15E-04 \| 0.02401783 \| 5.66E-01 \| -1.7673621 \| \| CTNND1 \| 9.83E+01 \| 3.93E-03 \| 0.04944595 \| 5.66E-01 \| -1.7670069 \| \| RCAN3 \| 1.88E+02 \| 3.40E-04 \| 0.01980958 \| 5.66E-01 \| -1.7669254 \| \| RXRB \| 7.87E+01 \| 4.87E-03 \| 0.05322635 \| 5.66E-01 \| -1.7661084 \| \| MIOS \| 1.40E+02 \| 9.34E-04 \| 0.02894859 \| 5.67E-01 \| -1.7645955 \| \| TAB1 \| 2.76E+01 \| 1.53E-04 \| 0.0162209 \| 5.67E-01 \| -1.7644045 \| \| UPRT \| 4.62E+01 \| 3.85E-03 \| 0.0487654 \| 5.68E-01 \| -1.7620532 \| \| PYCARD \| 8.44E+01 \| 5.29E-04 \| 0.02266365 \| 5.68E-01 \| -1.7602335 \| \| MYO1G \| 3.82E+02 \| 1.55E-03 \| 0.03496769 \| 5.68E-01 \| -1.7601368 \| \| TRPS1 \| 5.68E+02 \| 5.25E-04 \| 0.02266365 \| 5.68E-01 \| -1.7595323 \| \| EIF3H \| 5.24E+02 \| 4.95E-04 \| 0.0222881 \| 5.69E-01 \| -1.7578207 \| \| SRSF1 \| 1.09E+03 \| 4.87E-03 \| 0.05322635 \| 5.69E-01 \| -1.7570868 \| \| GTF3C2 \| 1.12E+02 \| 5.20E-04 \| 0.02266365 \| 5.69E-01 \| -1.7568203 \| \| NBPF15 \| 1.68E+02 \| 3.18E-05 \| 0.01358748 \| 5.70E-01 \| -1.7557867 \| \| HSD17B4 \| 2.40E+02 \| 2.15E-03 \| 0.03884006 \| 5.70E-01 \| -1.754955 \| \| SYK \| 1.38E+03 \| 3.88E-04 \| 0.02090486 \| 5.71E-01 \| -1.752631 \| \| PGLS \| 5.81E+01 \| 1.71E-03 \| 0.03557751 \| 5.71E-01 \| -1.7516316 \| \| ZC3HC1 \| 1.90E+01 \| 2.23E-03 \| 0.03968355 \| 5.71E-01 \| -1.7500829 \| \| PTER \| 7.95E+01 \| 1.28E-03 \| 0.03293681 \| 5.71E-01 \| -1.7498562 \| \| PGAM1 \| 6.11E+02 \| 4.77E-03 \| 0.05307894 \| 5.72E-01 \| -1.7495215 \| \| BTBD7 \| 2.87E+02 \| 1.18E-03 \| 0.03173681 \| 5.72E-01 \| -1.7493171 \| \| NUP98 \| 1.21E+03 \| 4.04E-03 \| 0.05014701 \| 5.72E-01 \| -1.7488092 \| \| ZDHHC21 \| 1.48E+02 \| 5.02E-04 \| 0.02238866 \| 5.72E-01 \| -1.7486809 \| \| AKAP8 \| 6.67E+01 \| 1.79E-03 \| 0.03585643 \| 5.72E-01 \| -1.7484057 \| \| ATP1A1 \| 5.12E+02 \| 1.17E-04 \| 0.01508103 \| 5.72E-01 \| -1.7476341 \| \| SULF2 \| 2.57E+02 \| 3.70E-03 \| 0.04772117 \| 5.72E-01 \| -1.7468827 \| \| PMF1 \| 5.41E+01 \| 2.85E-03 \| 0.0431483 \| 5.73E-01 \| -1.7465204 \| \| AOAH \| 7.63E+02 \| 1.83E-04 \| 0.01716907 \| 5.73E-01 \| -1.7459818 \| \| RRN3 \| 9.68E+01 \| 1.14E-03 \| 0.03112937 \| 5.73E-01 \| -1.7453261 \| \| IKBKB \| 1.83E+02 \| 9.41E-04 \| 0.02905449 \| 5.73E-01 \| -1.7452155 \| \| CTSH \| 1.63E+02 \| 5.44E-04 \| 0.02271088 \| 5.73E-01 \| -1.7451579 \| \| ZNF813 \| 3.73E+01 \| 1.66E-03 \| 0.03534598 \| 5.74E-01 \| -1.7436266 \| \| EBLN3P \| 3.82E+02 \| 1.41E-03 \| 0.03422635 \| 5.74E-01 \| -1.7434897 \| \| TMEM259 \| 9.43E+01 \| 2.96E-03 \| 0.04366854 \| 5.74E-01 \| -1.7423519 \| \| GALNT2 \| 2.59E+02 \| 9.31E-04 \| 0.0289153 \| 5.74E-01 \| -1.7415049 \| \| ZNF746 \| 3.31E+02 \| 1.35E-03 \| 0.0337411 \| 5.74E-01 \| -1.7406594 \| \| XPO4 \| 1.87E+02 \| 4.92E-03 \| 0.05336268 \| 5.75E-01 \| -1.7401253 \| \| THTPA \| 2.85E+01 \| 3.99E-03 \| 0.04977319 \| 5.75E-01 \| -1.7390007 \| \| KCNQ1 \| 6.71E+01 \| 3.02E-03 \| 0.04401166 \| 5.75E-01 \| -1.7379989 \| \| MRTFB \| 5.50E+01 \| 8.27E-04 \| 0.02791931 \| 5.76E-01 \| -1.7370447 \| \| ELP1 \| 1.04E+02 \| 6.05E-04 \| 0.02387379 \| 5.76E-01 \| -1.7368124 \| \| NOL6 \| 4.51E+01 \| 1.48E-03 \| 0.03434026 \| 5.76E-01 \| -1.7355083 \| \| TRIO \| 1.49E+02 \| 1.87E-04 \| 0.01718472 \| 5.76E-01 \| -1.735452 \| \| BBS7 \| 4.53E+01 \| 1.88E-03 \| 0.03654287 \| 5.77E-01 \| -1.7345491 \| \| FBL \| 1.11E+02 \| 4.49E-03 \| 0.05226789 \| 5.77E-01 \| -1.7318331 \| \| ANAPC2 \| 3.33E+01 \| 3.66E-03 \| 0.04756982 \| 5.77E-01 \| -1.7318232 \| \| IRAK4 \| 4.28E+02 \| 1.89E-04 \| 0.01718472 \| 5.78E-01 \| -1.7289756 \| \| TCEA1 \| 4.51E+02 \| 4.01E-03 \| 0.04988602 \| 5.78E-01 \| -1.7288502 \| \| DOP1A \| 2.06E+02 \| 5.11E-04 \| 0.02246294 \| 5.79E-01 \| -1.7280716 \| \| NOTCH2 \| 5.09E+03 \| 3.98E-03 \| 0.04975749 \| 5.79E-01 \| -1.7279923 \| \| VPS51 \| 7.03E+01 \| 1.51E-03 \| 0.03454272 \| 5.79E-01 \| -1.7276855 \| \| PCID2 \| 5.49E+01 \| 1.21E-03 \| 0.03218986 \| 5.79E-01 \| -1.7273445 \| \| MAD2L2 \| 3.75E+01 \| 4.01E-04 \| 0.0212069 \| 5.79E-01 \| -1.7268247 \| \| TBL1X \| 6.09E+02 \| 2.74E-03 \| 0.0429721 \| 5.79E-01 \| -1.7266784 \| \| RPS6 \| 1.29E+03 \| 2.68E-03 \| 0.04281263 \| 5.80E-01 \| -1.7254703 \| \| SCAI \| 6.20E+01 \| 6.00E-04 \| 0.02374826 \| 5.80E-01 \| -1.7253367 \| \| PGS1 \| 4.09E+02 \| 3.62E-03 \| 0.04740123 \| 5.80E-01 \| -1.7246225 \| \| KLHL36 \| 1.51E+02 \| 1.03E-03 \| 0.03034051 \| 5.80E-01 \| -1.7227836 \| \| APEH \| 1.14E+02 \| 2.03E-03 \| 0.03786659 \| 5.81E-01 \| -1.7220672 \| \| GNS \| 2.26E+03 \| 3.51E-03 \| 0.04678552 \| 5.81E-01 \| -1.7205671 \| \| VPS16 \| 6.53E+01 \| 2.57E-03 \| 0.04189743 \| 5.81E-01 \| -1.7199812 \| \| PI4K2A \| 5.70E+01 \| 4.36E-03 \| 0.05195107 \| 5.81E-01 \| -1.7197562 \| \| INIP \| 1.13E+02 \| 2.28E-03 \| 0.03979599 \| 5.82E-01 \| -1.7168008 \| \| GOLGA1 \| 1.15E+02 \| 2.63E-03 \| 0.04255273 \| 5.83E-01 \| -1.7158084 \| \| EOGT \| 4.82E+01 \| 3.05E-03 \| 0.04404908 \| 5.83E-01 \| -1.7144218 \| \| VPS52 \| 7.24E+01 \| 1.36E-03 \| 0.03400004 \| 5.83E-01 \| -1.7142858 \| \| HDAC3 \| 9.17E+01 \| 8.64E-04 \| 0.02849752 \| 5.83E-01 \| -1.7141014 \| \| NEMP2 \| 4.48E+01 \| 4.20E-03 \| 0.05102831 \| 5.84E-01 \| -1.712919 \| \| ATR \| 2.86E+02 \| 2.83E-04 \| 0.0184668 \| 5.84E-01 \| -1.7125193 \| \| POLR3E \| 3.94E+01 \| 2.06E-03 \| 0.03807473 \| 5.84E-01 \| -1.7120143 \| \| ACADVL \| 1.97E+02 \| 6.47E-04 \| 0.02457212 \| 5.84E-01 \| -1.7113007 \| \| MAFB \| 3.12E+02 \| 3.37E-03 \| 0.0462197 \| 5.85E-01 \| -1.7090415 \| \| NOA1 \| 7.93E+01 \| 1.87E-03 \| 0.03644446 \| 5.85E-01 \| -1.7087531 \| \| SH3TC1 \| 5.36E+01 \| 3.56E-03 \| 0.04711054 \| 5.86E-01 \| -1.7067352 \| \| PSAP \| 6.79E+03 \| 4.47E-03 \| 0.05224833 \| 5.86E-01 \| -1.7061688 \| \| LRRC58 \| 3.01E+02 \| 2.49E-03 \| 0.04130559 \| 5.86E-01 \| -1.7060738 \| \| ERCC1 \| 4.66E+01 \| 2.64E-03 \| 0.04256329 \| 5.87E-01 \| -1.7047416 \| \| S100PBP \| 1.86E+02 \| 1.85E-03 \| 0.03626429 \| 5.87E-01 \| -1.7037565 \| \| ITFG2 \| 3.94E+01 \| 2.95E-04 \| 0.01891181 \| 5.87E-01 \| -1.7035811 \| \| CALHM2 \| 6.11E+01 \| 5.09E-03 \| 0.05423186 \| 5.87E-01 \| -1.7035754 \| \| C19orf38 \| 1.21E+02 \| 4.89E-03 \| 0.05328933 \| 5.87E-01 \| -1.7034345 \| \| EIF2D \| 5.98E+01 \| 4.24E-03 \| 0.05124371 \| 5.87E-01 \| -1.7032331 \| \| PHB2 \| 1.11E+02 \| 1.72E-03 \| 0.03557815 \| 5.87E-01 \| -1.7029326 \| \| ANKRD10 \| 1.45E+02 \| 1.46E-03 \| 0.03434026 \| 5.87E-01 \| -1.7022433 \| \| NEPRO \| 1.18E+02 \| 2.00E-03 \| 0.03748326 \| 5.88E-01 \| -1.7016962 \| \| MAP3K20 \| 2.75E+02 \| 4.25E-03 \| 0.05124371 \| 5.88E-01 \| -1.7008673 \| \| CEBPG \| 1.54E+02 \| 1.21E-03 \| 0.03218986 \| 5.88E-01 \| -1.7007483 \| \| CEMIP2 \| 1.09E+03 \| 3.50E-03 \| 0.04671329 \| 5.88E-01 \| -1.7001318 \| \| UBTD1 \| 2.68E+01 \| 4.44E-03 \| 0.05222976 \| 5.88E-01 \| -1.6993697 \| \| PRORP \| 1.67E+02 \| 4.57E-04 \| 0.02201345 \| 5.89E-01 \| -1.6987573 \| \| FAM117B \| 1.92E+02 \| 4.79E-03 \| 0.05307894 \| 5.90E-01 \| -1.6939461 \| \| METTL23 \| 3.13E+01 \| 4.79E-03 \| 0.05307894 \| 5.91E-01 \| -1.6927464 \| \| RAPGEF6 \| 7.98E+02 \| 1.49E-03 \| 0.03434026 \| 5.91E-01 \| -1.6921641 \| \| STAT6 \| 1.12E+03 \| 6.34E-04 \| 0.02443204 \| 5.91E-01 \| -1.6920798 \| \| RPS6KA1 \| 9.44E+02 \| 3.62E-03 \| 0.04740123 \| 5.91E-01 \| -1.691777 \| \| SHPRH \| 1.82E+02 \| 2.57E-04 \| 0.01800472 \| 5.91E-01 \| -1.6914099 \| \| CDV3 \| 1.24E+03 \| 2.90E-03 \| 0.04340126 \| 5.92E-01 \| -1.6901481 \| \| ABCB7 \| 7.95E+01 \| 3.79E-03 \| 0.04843566 \| 5.92E-01 \| -1.6897369 \| \| EIF3M \| 3.22E+02 \| 3.83E-03 \| 0.04859009 \| 5.92E-01 \| -1.6891194 \| \| M6PR \| 4.72E+02 \| 5.08E-03 \| 0.05411992 \| 5.92E-01 \| -1.6887727 \| \| USP34 \| 2.42E+03 \| 2.29E-03 \| 0.03979739 \| 5.92E-01 \| -1.6887695 \| \| POR \| 1.74E+02 \| 5.05E-03 \| 0.05391586 \| 5.93E-01 \| -1.68761 \| \| EHD4 \| 9.22E+01 \| 2.26E-03 \| 0.0397705 \| 5.93E-01 \| -1.687016 \| \| NCKAP1L \| 1.86E+03 \| 1.97E-03 \| 0.03723394 \| 5.93E-01 \| -1.6869713 \| \| MED12 \| 3.73E+02 \| 4.64E-03 \| 0.0526918 \| 5.93E-01 \| -1.685334 \| \| UHRF1BP1L \| 9.92E+02 \| 4.39E-03 \| 0.05197104 \| 5.93E-01 \| -1.6850205 \| \| ARHGAP35 \| 2.08E+02 \| 2.84E-04 \| 0.0184668 \| 5.93E-01 \| -1.6849353 \| \| SCPEP1 \| 2.86E+02 \| 4.51E-03 \| 0.05227731 \| 5.95E-01 \| -1.6815371 \| \| TMEM19 \| 7.14E+01 \| 4.26E-03 \| 0.05129063 \| 5.95E-01 \| -1.6813972 \| \| INTS9 \| 4.14E+01 \| 4.30E-04 \| 0.02156426 \| 5.95E-01 \| -1.6810969 \| \| WDR55 \| 6.81E+01 \| 1.29E-03 \| 0.03293681 \| 5.95E-01 \| -1.6805375 \| \| WDR59 \| 8.66E+01 \| 4.82E-03 \| 0.05312425 \| 5.95E-01 \| -1.6797167 \| \| ERVK13-1 \| 1.36E+02 \| 6.33E-05 \| 0.01358748 \| 5.96E-01 \| -1.6773614 \| \| DYM \| 2.55E+02 \| 3.50E-03 \| 0.04671329 \| 5.97E-01 \| -1.6753167 \| \| APH1A \| 1.49E+02 \| 4.77E-03 \| 0.05307894 \| 5.97E-01 \| -1.6738475 \| \| HEATR6 \| 5.42E+01 \| 8.31E-04 \| 0.02791931 \| 5.97E-01 \| -1.673742 \| \| MTRR \| 1.26E+02 \| 2.74E-03 \| 0.0429721 \| 5.98E-01 \| -1.6730036 \| \| CLK3 \| 2.89E+02 \| 4.69E-04 \| 0.02213483 \| 5.98E-01 \| -1.6714799 \| \| DENND6A \| 2.24E+02 \| 2.34E-03 \| 0.04014549 \| 5.99E-01 \| -1.6707984 \| \| FGD5-AS1 \| 5.89E+02 \| 3.08E-03 \| 0.04416241 \| 5.99E-01 \| -1.6706377 \| \| IPP \| 3.53E+01 \| 1.60E-03 \| 0.03500513 \| 5.99E-01 \| -1.6700036 \| \| PSMD2 \| 2.69E+02 \| 1.95E-04 \| 0.01723175 \| 5.99E-01 \| -1.6699036 \| \| PTOV1 \| 3.68E+01 \| 1.20E-03 \| 0.03213487 \| 5.99E-01 \| -1.6691408 \| \| TUFM \| 1.39E+02 \| 2.85E-03 \| 0.0431483 \| 5.99E-01 \| -1.6690566 \| \| CLPX \| 2.28E+02 \| 4.15E-03 \| 0.05084268 \| 5.99E-01 \| -1.6690222 \| \| PPP1CA \| 2.25E+02 \| 5.23E-04 \| 0.02266365 \| 5.99E-01 \| -1.6687394 \| \| TIAL1 \| 3.60E+02 \| 1.44E-03 \| 0.03422635 \| 6.00E-01 \| -1.6680185 \| \| ARF6 \| 8.85E+02 \| 4.37E-04 \| 0.02174045 \| 6.00E-01 \| -1.6659244 \| \| SCYL1 \| 1.41E+02 \| 4.17E-04 \| 0.02150161 \| 6.01E-01 \| -1.6650807 \| \| KYAT3 \| 1.13E+02 \| 3.17E-03 \| 0.04497545 \| 6.01E-01 \| -1.6639937 \| \| SRSF2 \| 2.59E+02 \| 2.76E-03 \| 0.0430014 \| 6.02E-01 \| -1.66123 \| \| DDX42 \| 2.21E+02 \| 8.54E-05 \| 0.01407615 \| 6.02E-01 \| -1.6607374 \| \| TTC31 \| 4.02E+01 \| 8.63E-04 \| 0.02849752 \| 6.03E-01 \| -1.6586522 \| \| YTHDC2 \| 2.29E+02 \| 1.67E-03 \| 0.03534598 \| 6.03E-01 \| -1.6570792 \| \| CAND1 \| 4.16E+02 \| 3.98E-03 \| 0.04975749 \| 6.04E-01 \| -1.6557794 \| \| TTC19 \| 5.16E+01 \| 2.41E-03 \| 0.04082713 \| 6.04E-01 \| -1.6557397 \| \| COPS2 \| 3.94E+02 \| 4.29E-03 \| 0.05141044 \| 6.04E-01 \| -1.6548286 \| \| TWF2 \| 2.41E+02 \| 2.70E-03 \| 0.04291402 \| 6.06E-01 \| -1.6495477 \| \| WDR36 \| 1.85E+02 \| 3.55E-03 \| 0.04711054 \| 6.06E-01 \| -1.6495299 \| \| CPNE2 \| 6.15E+01 \| 2.48E-03 \| 0.0412902 \| 6.07E-01 \| -1.6467914 \| \| EIF4B \| 1.31E+03 \| 1.81E-03 \| 0.03592144 \| 6.08E-01 \| -1.6452633 \| \| PTCD3 \| 1.94E+02 \| 2.83E-03 \| 0.0431483 \| 6.08E-01 \| -1.6446789 \| \| GAB3 \| 2.26E+02 \| 3.12E-04 \| 0.01931625 \| 6.08E-01 \| -1.6444016 \| \| ELOVL1 \| 1.00E+02 \| 2.46E-03 \| 0.0411277 \| 6.08E-01 \| -1.6438498 \| \| RPL19 \| 1.11E+03 \| 2.21E-03 \| 0.03940971 \| 6.09E-01 \| -1.642944 \| \| MTCH2 \| 7.44E+01 \| 3.01E-03 \| 0.04391029 \| 6.09E-01 \| -1.6419981 \| \| VIRMA \| 4.26E+02 \| 2.50E-03 \| 0.0414038 \| 6.09E-01 \| -1.641294 \| \| SUCLG2 \| 1.28E+02 \| 4.28E-03 \| 0.05135747 \| 6.10E-01 \| -1.6404559 \| \| UQCRC1 \| 1.12E+02 \| 2.10E-03 \| 0.03828352 \| 6.10E-01 \| -1.6398257 \| \| SUGT1 \| 2.83E+02 \| 2.43E-03 \| 0.04084419 \| 6.10E-01 \| -1.638055 \| \| UTP25 \| 6.90E+01 \| 1.20E-03 \| 0.03213487 \| 6.11E-01 \| -1.6375731 \| \| KDM3A \| 4.96E+02 \| 2.65E-04 \| 0.01810876 \| 6.11E-01 \| -1.635805 \| \| MAN2B1 \| 2.86E+02 \| 7.08E-05 \| 0.01361826 \| 6.12E-01 \| -1.6348178 \| \| EIF4E2 \| 1.75E+02 \| 2.81E-04 \| 0.0184668 \| 6.12E-01 \| -1.6344355 \| \| HADHB \| 3.55E+02 \| 4.79E-03 \| 0.05307894 \| 6.12E-01 \| -1.6340279 \| \| RER1 \| 2.04E+02 \| 4.72E-03 \| 0.05303658 \| 6.12E-01 \| -1.6340203 \| \| UBXN1 \| 7.29E+01 \| 1.92E-03 \| 0.03697811 \| 6.12E-01 \| -1.6339591 \| \| IRGQ \| 5.74E+01 \| 1.49E-03 \| 0.03434026 \| 6.12E-01 \| -1.6337969 \| \| LOC100294145 \| 4.30E+01 \| 1.29E-03 \| 0.03293681 \| 6.12E-01 \| -1.6332147 \| \| ACSS2 \| 1.52E+02 \| 3.61E-03 \| 0.04739647 \| 6.13E-01 \| -1.6318995 \| \| UFL1 \| 1.47E+02 \| 2.31E-03 \| 0.03980839 \| 6.13E-01 \| -1.6315224 \| \| CPNE1 \| 3.44E+02 \| 3.36E-04 \| 0.01980958 \| 6.13E-01 \| -1.6311809 \| \| AOPEP \| 3.39E+01 \| 1.53E-03 \| 0.03462625 \| 6.13E-01 \| -1.6308894 \| \| GTF3C3 \| 8.35E+01 \| 5.02E-03 \| 0.05376205 \| 6.13E-01 \| -1.6306512 \| \| LRPPRC \| 2.30E+02 \| 1.01E-03 \| 0.0302405 \| 6.14E-01 \| -1.6295081 \| \| ALG13 \| 1.34E+02 \| 1.90E-03 \| 0.03697811 \| 6.14E-01 \| -1.6282606 \| \| SEPTIN2 \| 1.06E+03 \| 3.07E-03 \| 0.04416241 \| 6.14E-01 \| -1.6274755 \| \| HECTD1 \| 8.81E+02 \| 2.85E-03 \| 0.0431483 \| 6.15E-01 \| -1.6267915 \| \| ZNF460 \| 3.14E+02 \| 4.50E-03 \| 0.05227731 \| 6.15E-01 \| -1.6248818 \| \| KXD1 \| 7.46E+01 \| 4.67E-03 \| 0.05289108 \| 6.15E-01 \| -1.6247766 \| \| EIF2B1 \| 8.96E+01 \| 3.31E-03 \| 0.04581592 \| 6.16E-01 \| -1.6244043 \| \| HDAC1 \| 2.37E+02 \| 1.47E-03 \| 0.03434026 \| 6.16E-01 \| -1.6242206 \| \| SIK3 \| 4.30E+02 \| 6.32E-05 \| 0.01358748 \| 6.16E-01 \| -1.6232922 \| \| FOXN3 \| 9.54E+02 \| 1.33E-03 \| 0.03352942 \| 6.16E-01 \| -1.6221939 \| \| INTS11 \| 4.73E+01 \| 3.65E-03 \| 0.04754042 \| 6.17E-01 \| -1.6219806 \| \| EIF2B5 \| 5.12E+01 \| 8.82E-04 \| 0.02862553 \| 6.17E-01 \| -1.6204251 \| \| CD99L2 \| 8.08E+01 \| 3.59E-03 \| 0.04729405 \| 6.17E-01 \| -1.6201302 \| \| DDX17 \| 3.53E+03 \| 4.93E-03 \| 0.0534573 \| 6.18E-01 \| -1.6191639 \| \| NDUFS2 \| 1.06E+02 \| 2.44E-04 \| 0.01800472 \| 6.18E-01 \| -1.6186358 \| \| ZNF263 \| 4.68E+01 \| 2.08E-04 \| 0.01737605 \| 6.18E-01 \| -1.6173057 \| \| GIMAP8 \| 3.86E+02 \| 2.32E-03 \| 0.03995945 \| 6.18E-01 \| -1.6169464 \| \| LRCH1 \| 1.14E+02 \| 1.23E-03 \| 0.03244251 \| 6.19E-01 \| -1.6159876 \| \| IDH3B \| 5.64E+01 \| 1.48E-03 \| 0.03434026 \| 6.19E-01 \| -1.6158251 \| \| PREP \| 1.13E+02 \| 1.77E-03 \| 0.03579792 \| 6.19E-01 \| -1.6148983 \| \| MRE11 \| 1.75E+02 \| 1.71E-03 \| 0.03557751 \| 6.20E-01 \| -1.6134854 \| \| NBPF8 \| 1.18E+02 \| 1.52E-03 \| 0.03460547 \| 6.20E-01 \| -1.613479 \| \| PIP5K1A \| 2.50E+02 \| 3.48E-03 \| 0.04667504 \| 6.20E-01 \| -1.613257 \| \| DGCR8 \| 4.32E+01 \| 5.61E-04 \| 0.02281591 \| 6.20E-01 \| -1.6128729 \| \| PATJ \| 6.74E+01 \| 1.81E-03 \| 0.03592144 \| 6.20E-01 \| -1.6125502 \| \| RPS6KB2 \| 5.55E+01 \| 1.98E-03 \| 0.03723394 \| 6.20E-01 \| -1.612397 \| \| MTMR14 \| 1.87E+02 \| 1.49E-03 \| 0.03434026 \| 6.20E-01 \| -1.6123901 \| \| RIOX2 \| 6.18E+01 \| 2.06E-03 \| 0.03807473 \| 6.21E-01 \| -1.6115501 \| \| STK25 \| 3.99E+01 \| 8.80E-04 \| 0.02862553 \| 6.21E-01 \| -1.6109608 \| \| COG8 \| 8.00E+01 \| 2.27E-03 \| 0.0397705 \| 6.21E-01 \| -1.6107792 \| \| UTP18 \| 6.52E+01 \| 3.79E-03 \| 0.04843566 \| 6.21E-01 \| -1.6106957 \| \| EID1 \| 2.19E+02 \| 2.81E-03 \| 0.0431483 \| 6.21E-01 \| -1.6094085 \| \| TCP11L1 \| 8.40E+01 \| 4.63E-03 \| 0.05260685 \| 6.21E-01 \| -1.6093377 \| \| ZEB2 \| 2.14E+03 \| 4.67E-04 \| 0.02209467 \| 6.21E-01 \| -1.6092885 \| \| COMT \| 5.51E+01 \| 2.43E-03 \| 0.04084419 \| 6.21E-01 \| -1.6090546 \| \| CPSF7 \| 3.02E+02 \| 3.45E-03 \| 0.04653622 \| 6.22E-01 \| -1.608154 \| \| HNRNPA1L2 \| 6.02E+01 \| 1.67E-03 \| 0.03534598 \| 6.22E-01 \| -1.6069812 \| \| ELP2 \| 1.02E+02 \| 3.94E-03 \| 0.04944595 \| 6.23E-01 \| -1.604253 \| \| IGBP1 \| 1.79E+02 \| 1.48E-03 \| 0.03434026 \| 6.23E-01 \| -1.6039095 \| \| COG5 \| 2.58E+02 \| 1.76E-03 \| 0.03573192 \| 6.24E-01 \| -1.6032357 \| \| USP21 \| 3.71E+01 \| 2.75E-03 \| 0.04297444 \| 6.24E-01 \| -1.6027916 \| \| NT5DC1 \| 6.51E+01 \| 4.31E-03 \| 0.05158979 \| 6.24E-01 \| -1.6019069 \| \| WDFY4 \| 8.55E+02 \| 1.04E-03 \| 0.03034051 \| 6.25E-01 \| -1.5997073 \| \| COMMD9 \| 7.78E+01 \| 8.31E-04 \| 0.02791931 \| 6.25E-01 \| -1.5992176 \| \| YKT6 \| 1.10E+02 \| 2.56E-03 \| 0.04188029 \| 6.25E-01 \| -1.5988074 \| \| PITPNA \| 1.70E+02 \| 4.35E-03 \| 0.05185301 \| 6.26E-01 \| -1.5978707 \| \| FAM204A \| 2.28E+02 \| 5.97E-04 \| 0.02372349 \| 6.26E-01 \| -1.5974255 \| \| EIF3D \| 2.74E+02 \| 4.03E-04 \| 0.0212069 \| 6.27E-01 \| -1.5958 \| \| SREK1 \| 1.69E+02 \| 5.11E-04 \| 0.02246294 \| 6.27E-01 \| -1.5949313 \| \| TRIM34 \| 1.27E+02 \| 4.04E-04 \| 0.0212069 \| 6.27E-01 \| -1.5938953 \| \| ZNF529 \| 4.72E+01 \| 3.50E-03 \| 0.04671329 \| 6.28E-01 \| -1.5929138 \| \| TBC1D25 \| 7.94E+01 \| 3.50E-03 \| 0.04672053 \| 6.28E-01 \| -1.5915749 \| \| PAIP1 \| 9.49E+01 \| 4.55E-03 \| 0.05231298 \| 6.28E-01 \| -1.5911859 \| \| ZDHHC3 \| 4.47E+02 \| 1.20E-04 \| 0.01508103 \| 6.29E-01 \| -1.5910018 \| \| KIAA1143 \| 1.51E+02 \| 2.99E-03 \| 0.04383474 \| 6.29E-01 \| -1.5908272 \| \| TNKS \| 3.10E+02 \| 2.56E-03 \| 0.04188029 \| 6.29E-01 \| -1.5903117 \| \| TOP3A \| 2.15E+02 \| 2.58E-03 \| 0.04198582 \| 6.29E-01 \| -1.5893362 \| \| SIRT2 \| 7.93E+01 \| 1.44E-03 \| 0.03422635 \| 6.29E-01 \| -1.5887408 \| \| KATNB1 \| 4.70E+01 \| 4.12E-03 \| 0.05066207 \| 6.30E-01 \| -1.5885395 \| \| ASMTL \| 4.26E+01 \| 5.37E-04 \| 0.02271088 \| 6.30E-01 \| -1.5868181 \| \| ZNF248 \| 3.63E+01 \| 1.38E-03 \| 0.03422635 \| 6.30E-01 \| -1.5866735 \| \| ORMDL1 \| 1.32E+02 \| 5.14E-03 \| 0.05444322 \| 6.30E-01 \| -1.5865078 \| \| TULP4 \| 2.60E+02 \| 8.37E-04 \| 0.02796205 \| 6.31E-01 \| -1.5853393 \| \| PIN1 \| 2.60E+01 \| 4.46E-03 \| 0.05222976 \| 6.31E-01 \| -1.5849277 \| \| GUSB \| 1.02E+02 \| 2.48E-03 \| 0.0412902 \| 6.32E-01 \| -1.5834089 \| \| TRAK1 \| 2.02E+02 \| 1.51E-03 \| 0.03454272 \| 6.32E-01 \| -1.5831428 \| \| ZDHHC6 \| 1.52E+02 \| 2.11E-03 \| 0.03833044 \| 6.32E-01 \| -1.582717 \| \| KLHL18 \| 1.47E+02 \| 3.98E-03 \| 0.04975749 \| 6.32E-01 \| -1.5825479 \| \| XYLT1 \| 1.95E+02 \| 1.92E-03 \| 0.03697811 \| 6.32E-01 \| -1.5822298 \| \| IRF5 \| 1.11E+02 \| 4.42E-03 \| 0.05205649 \| 6.32E-01 \| -1.5813911 \| \| MFNG \| 1.01E+02 \| 3.08E-03 \| 0.04416241 \| 6.33E-01 \| -1.5808733 \| \| TBC1D22A \| 1.59E+02 \| 8.92E-04 \| 0.02862553 \| 6.33E-01 \| -1.5800027 \| \| PI4KA \| 3.82E+02 \| 3.26E-03 \| 0.04541638 \| 6.33E-01 \| -1.5792564 \| \| HADHA \| 7.21E+02 \| 1.42E-03 \| 0.03422635 \| 6.33E-01 \| -1.5788247 \| \| MED14 \| 2.80E+02 \| 3.16E-03 \| 0.04494409 \| 6.34E-01 \| -1.578233 \| \| CCAR2 \| 1.53E+02 \| 6.78E-04 \| 0.02505425 \| 6.34E-01 \| -1.5780198 \| \| ARFGEF2 \| 3.63E+02 \| 2.80E-03 \| 0.0431483 \| 6.34E-01 \| -1.5760584 \| \| ESD \| 9.72E+01 \| 3.70E-03 \| 0.04772117 \| 6.35E-01 \| -1.5760364 \| \| WDR70 \| 6.73E+01 \| 1.97E-03 \| 0.03723394 \| 6.36E-01 \| -1.5729089 \| \| SARS1 \| 2.13E+02 \| 4.86E-03 \| 0.05322635 \| 6.36E-01 \| -1.572473 \| \| NPC1 \| 1.31E+02 \| 2.77E-03 \| 0.04307144 \| 6.36E-01 \| -1.5718953 \| \| UBR5 \| 1.35E+03 \| 2.08E-03 \| 0.03820965 \| 6.36E-01 \| -1.5718721 \| \| SMARCAD1 \| 9.21E+01 \| 5.46E-04 \| 0.02271088 \| 6.36E-01 \| -1.5718673 \| \| WDR77 \| 3.18E+01 \| 1.92E-03 \| 0.03697811 \| 6.37E-01 \| -1.5707278 \| \| IPO5 \| 2.03E+02 \| 1.89E-03 \| 0.03683985 \| 6.37E-01 \| -1.5704335 \| \| PIGG \| 1.09E+02 \| 5.32E-04 \| 0.02269051 \| 6.37E-01 \| -1.5703375 \| \| SUGP2 \| 2.04E+02 \| 2.59E-03 \| 0.0420695 \| 6.37E-01 \| -1.570298 \| \| TAX1BP1 \| 6.91E+02 \| 4.89E-03 \| 0.05328933 \| 6.37E-01 \| -1.5700785 \| \| FLII \| 5.98E+02 \| 2.90E-03 \| 0.04340126 \| 6.37E-01 \| -1.5698623 \| \| STX5 \| 1.18E+02 \| 2.05E-03 \| 0.03807473 \| 6.37E-01 \| -1.5697814 \| \| SS18L1 \| 3.33E+01 \| 4.52E-03 \| 0.05231298 \| 6.37E-01 \| -1.569534 \| \| CANT1 \| 4.68E+02 \| 4.13E-03 \| 0.05070752 \| 6.38E-01 \| -1.5669204 \| \| TAF5L \| 7.76E+01 \| 4.70E-03 \| 0.05301601 \| 6.38E-01 \| -1.5664175 \| \| APRT \| 2.26E+01 \| 3.15E-03 \| 0.04486926 \| 6.38E-01 \| -1.5664073 \| \| POFUT1 \| 7.69E+01 \| 1.04E-03 \| 0.03034051 \| 6.38E-01 \| -1.5663327 \| \| USP24 \| 8.02E+02 \| 1.59E-03 \| 0.03496769 \| 6.39E-01 \| -1.5653516 \| \| SRSF11 \| 2.50E+02 \| 1.31E-04 \| 0.01557706 \| 6.39E-01 \| -1.5643082 \| \| TIMM10B \| 9.06E+01 \| 1.23E-03 \| 0.03244251 \| 6.39E-01 \| -1.5640929 \| \| SIK2 \| 7.64E+01 \| 4.07E-03 \| 0.05031704 \| 6.40E-01 \| -1.5632716 \| \| BECN1 \| 2.39E+02 \| 2.66E-03 \| 0.04273147 \| 6.40E-01 \| -1.5629931 \| \| DPEP2 \| 1.41E+02 \| 4.86E-03 \| 0.05322635 \| 6.40E-01 \| -1.5625399 \| \| GRB2 \| 1.40E+03 \| 3.95E-03 \| 0.04948419 \| 6.40E-01 \| -1.5621624 \| \| AKAP11 \| 4.83E+02 \| 1.28E-03 \| 0.03293681 \| 6.41E-01 \| -1.5606519 \| \| SDAD1 \| 9.28E+01 \| 8.01E-04 \| 0.02766819 \| 6.41E-01 \| -1.560539 \| \| CSNK2B \| 1.13E+02 \| 3.05E-04 \| 0.01914308 \| 6.41E-01 \| -1.56001 \| \| MALT1 \| 3.25E+02 \| 1.77E-03 \| 0.03579792 \| 6.41E-01 \| -1.5592023 \| \| GABARAP \| 1.24E+03 \| 3.43E-03 \| 0.04634965 \| 6.41E-01 \| -1.5589876 \| \| UBE4A \| 6.84E+02 \| 3.31E-03 \| 0.04581592 \| 6.42E-01 \| -1.5587697 \| \| MAP3K4 \| 1.14E+02 \| 4.28E-04 \| 0.02156426 \| 6.42E-01 \| -1.5576181 \| \| ATG16L1 \| 1.05E+02 \| 4.91E-03 \| 0.05331697 \| 6.42E-01 \| -1.5571106 \| \| GUF1 \| 8.40E+01 \| 4.45E-03 \| 0.05222976 \| 6.42E-01 \| -1.5566988 \| \| ERCC6L2 \| 3.01E+02 \| 9.68E-05 \| 0.01494027 \| 6.43E-01 \| -1.5562963 \| \| PTPRE \| 1.51E+03 \| 5.02E-03 \| 0.05376205 \| 6.43E-01 \| -1.5562377 \| \| POLD3 \| 1.25E+02 \| 7.79E-04 \| 0.02728247 \| 6.43E-01 \| -1.5554465 \| \| CYFIP2 \| 7.38E+02 \| 2.20E-03 \| 0.03926859 \| 6.44E-01 \| -1.5528475 \| \| E2F4 \| 1.50E+02 \| 4.62E-03 \| 0.05256331 \| 6.44E-01 \| -1.5526625 \| \| TSC1 \| 1.93E+02 \| 1.37E-03 \| 0.03405687 \| 6.44E-01 \| -1.5520248 \| \| GPR108 \| 1.60E+02 \| 1.85E-03 \| 0.03626429 \| 6.45E-01 \| -1.55086 \| \| PSMB7 \| 8.93E+01 \| 2.86E-03 \| 0.0431483 \| 6.46E-01 \| -1.5488007 \| \| HNRNPA0 \| 1.73E+02 \| 5.14E-03 \| 0.05444322 \| 6.46E-01 \| -1.5486961 \| \| MEF2C \| 4.28E+02 \| 1.04E-03 \| 0.03034051 \| 6.46E-01 \| -1.5484705 \| \| EEF1D \| 5.06E+02 \| 6.53E-04 \| 0.024654 \| 6.46E-01 \| -1.5480959 \| \| AP1M1 \| 1.64E+02 \| 4.81E-03 \| 0.05307894 \| 6.46E-01 \| -1.5468414 \| \| LARP4B \| 5.86E+02 \| 2.52E-03 \| 0.04154566 \| 6.47E-01 \| -1.5462153 \| \| PSMD4 \| 2.32E+02 \| 3.04E-03 \| 0.04404908 \| 6.47E-01 \| -1.5461832 \| \| PLSCR3 \| 3.46E+01 \| 3.59E-03 \| 0.04729405 \| 6.47E-01 \| -1.5452194 \| \| TPR \| 8.80E+02 \| 4.39E-03 \| 0.05197104 \| 6.47E-01 \| -1.5446376 \| \| LIMD1 \| 1.82E+02 \| 3.31E-03 \| 0.04581592 \| 6.48E-01 \| -1.5438892 \| \| PIKFYVE \| 1.05E+03 \| 2.81E-03 \| 0.0431483 \| 6.48E-01 \| -1.5430853 \| \| EZH1 \| 3.07E+02 \| 4.19E-03 \| 0.05101155 \| 6.48E-01 \| -1.5430192 \| \| ACTR1A \| 2.65E+02 \| 8.06E-04 \| 0.02766819 \| 6.48E-01 \| -1.5427076 \| \| EIF3F \| 2.29E+02 \| 2.93E-03 \| 0.04348498 \| 6.48E-01 \| -1.5421427 \| \| SACS \| 1.87E+02 \| 1.36E-03 \| 0.03393026 \| 6.49E-01 \| -1.541707 \| \| GNB2 \| 6.28E+02 \| 2.10E-03 \| 0.03828352 \| 6.49E-01 \| -1.5409177 \| \| ZNF33B \| 9.07E+01 \| 4.20E-03 \| 0.05101218 \| 6.49E-01 \| -1.5406787 \| \| APLP2 \| 2.18E+03 \| 5.07E-03 \| 0.05411025 \| 6.50E-01 \| -1.5386378 \| \| GBA2 \| 1.67E+02 \| 4.82E-03 \| 0.05307894 \| 6.50E-01 \| -1.53728 \| \| KDM2B \| 6.74E+01 \| 4.90E-03 \| 0.05331697 \| 6.51E-01 \| -1.5361756 \| \| POLR1B \| 9.00E+01 \| 3.48E-03 \| 0.0467129 \| 6.51E-01 \| -1.5357569 \| \| PI4KB \| 2.83E+02 \| 2.93E-04 \| 0.01891181 \| 6.51E-01 \| -1.5356659 \| \| PHF14 \| 1.41E+02 \| 3.66E-04 \| 0.02063449 \| 6.52E-01 \| -1.5334221 \| \| PTPN11 \| 4.45E+02 \| 1.59E-03 \| 0.03496769 \| 6.52E-01 \| -1.5327469 \| \| NSUN4 \| 5.61E+01 \| 4.42E-03 \| 0.05205649 \| 6.53E-01 \| -1.5320971 \| \| PTPN2 \| 1.81E+02 \| 1.13E-03 \| 0.0310461 \| 6.53E-01 \| -1.5319869 \| \| DKFZP586I1420 \| 1.15E+02 \| 4.48E-03 \| 0.05226789 \| 6.53E-01 \| -1.5316647 \| \| NUDT5 \| 1.18E+02 \| 7.49E-04 \| 0.02646243 \| 6.54E-01 \| -1.5289697 \| \| ZNF225 \| 3.24E+01 \| 3.60E-03 \| 0.04735767 \| 6.54E-01 \| -1.5289439 \| \| EXOC4 \| 2.84E+02 \| 3.15E-04 \| 0.01931625 \| 6.54E-01 \| -1.5287607 \| \| AGO1 \| 5.40E+02 \| 1.45E-03 \| 0.03434026 \| 6.54E-01 \| -1.5287419 \| \| PRPF38B \| 2.07E+02 \| 1.42E-03 \| 0.03422635 \| 6.55E-01 \| -1.526496 \| \| DELE1 \| 9.40E+01 \| 1.28E-03 \| 0.03293681 \| 6.56E-01 \| -1.5245348 \| \| FASTKD2 \| 8.11E+01 \| 4.53E-03 \| 0.05231298 \| 6.56E-01 \| -1.5234354 \| \| RBMX \| 3.21E+02 \| 2.19E-03 \| 0.03918397 \| 6.57E-01 \| -1.5213841 \| \| BCLAF1 \| 1.01E+03 \| 1.66E-03 \| 0.03534598 \| 6.58E-01 \| -1.5208577 \| \| IKBKE \| 5.47E+01 \| 4.84E-03 \| 0.05322635 \| 6.58E-01 \| -1.5207371 \| \| ARFGAP2 \| 1.35E+02 \| 3.72E-03 \| 0.04789609 \| 6.58E-01 \| -1.5199423 \| \| NACA \| 4.65E+02 \| 4.69E-03 \| 0.05300111 \| 6.59E-01 \| -1.5183922 \| \| CHMP7 \| 1.66E+02 \| 4.73E-03 \| 0.05304222 \| 6.59E-01 \| -1.5182341 \| \| CENPB \| 6.41E+01 \| 1.58E-03 \| 0.03496769 \| 6.59E-01 \| -1.5181619 \| \| THAP12 \| 2.72E+02 \| 2.05E-03 \| 0.03807473 \| 6.59E-01 \| -1.5179551 \| \| RALGAPA1P1 \| 1.82E+02 \| 1.52E-04 \| 0.0161758 \| 6.59E-01 \| -1.5177944 \| \| ADCY9 \| 3.79E+01 \| 4.95E-03 \| 0.0534573 \| 6.60E-01 \| -1.5155293 \| \| RALGAPA1 \| 1.25E+02 \| 5.48E-04 \| 0.02272904 \| 6.61E-01 \| -1.5124238 \| \| PRPF18 \| 1.29E+02 \| 1.03E-03 \| 0.03034051 \| 6.61E-01 \| -1.5120427 \| \| PSPC1 \| 3.30E+02 \| 1.73E-03 \| 0.03557815 \| 6.62E-01 \| -1.5103351 \| \| KDM4C \| 3.26E+02 \| 2.71E-03 \| 0.04291879 \| 6.62E-01 \| -1.5100935 \| \| RPL11 \| 3.84E+02 \| 2.50E-03 \| 0.0414038 \| 6.63E-01 \| -1.5085954 \| \| PPP4C \| 1.84E+02 \| 2.92E-03 \| 0.04346124 \| 6.63E-01 \| -1.5083756 \| \| FBXO34 \| 2.39E+02 \| 1.25E-03 \| 0.03271222 \| 6.63E-01 \| -1.5077862 \| \| HNRNPDL \| 2.90E+02 \| 6.35E-04 \| 0.02443204 \| 6.65E-01 \| -1.504118 \| \| NSUN2 \| 1.72E+02 \| 9.29E-04 \| 0.02891446 \| 6.65E-01 \| -1.5029962 \| \| ZPR1 \| 7.27E+01 \| 3.66E-03 \| 0.04756982 \| 6.65E-01 \| -1.5029956 \| \| CREBZF \| 2.93E+02 \| 2.10E-03 \| 0.03828352 \| 6.66E-01 \| -1.5010164 \| \| FCHSD2 \| 5.11E+02 \| 2.73E-03 \| 0.0429721 \| 6.66E-01 \| -1.5005757 \| \| PSME1 \| 3.77E+02 \| 3.49E-03 \| 0.0467129 \| 1.51E+00 \| 1.5103908 \| \| JADE2 \| 2.29E+02 \| 5.10E-03 \| 0.05426585 \| 1.56E+00 \| 1.55988904 \| \| KPNA2 \| 5.89E+01 \| 5.11E-04 \| 0.02246294 \| 1.62E+00 \| 1.61507102 \| \| BLZF1 \| 2.13E+02 \| 2.76E-04 \| 0.01841921 \| 1.62E+00 \| 1.62483103 \| \| CARD16 \| 2.87E+02 \| 3.04E-03 \| 0.04404908 \| 1.63E+00 \| 1.626558 \| \| BAK1 \| 6.54E+01 \| 3.05E-03 \| 0.04404908 \| 1.64E+00 \| 1.63700533 \| \| DNAJC1 \| 6.00E+01 \| 3.65E-03 \| 0.04754042 \| 1.64E+00 \| 1.64049849 \| \| AGPAT1 \| 2.33E+02 \| 4.16E-03 \| 0.05088729 \| 1.66E+00 \| 1.65698876 \| \| XYLT2 \| 3.32E+01 \| 1.81E-03 \| 0.03592144 \| 1.66E+00 \| 1.66303073 \| \| FOXD2-AS1 \| 8.25E+00 \| 5.02E-03 \| 0.05376205 \| 1.68E+00 \| 1.68268949 \| \| PPFIBP2 \| 6.61E+01 \| 4.72E-03 \| 0.05303553 \| 1.70E+00 \| 1.70253021 \| \| TMEM140 \| 6.65E+02 \| 2.83E-03 \| 0.0431483 \| 1.71E+00 \| 1.70938138 \| \| PNPT1 \| 9.07E+01 \| 9.28E-04 \| 0.02891446 \| 1.75E+00 \| 1.74897067 \| \| BTN3A2 \| 5.44E+02 \| 3.21E-03 \| 0.0450618 \| 1.79E+00 \| 1.79152147 \| \| NBN \| 9.71E+02 \| 7.92E-05 \| 0.01392625 \| 1.80E+00 \| 1.79701853 \| \| ZNF493 \| 1.94E+02 \| 2.86E-03 \| 0.0431483 \| 1.80E+00 \| 1.80422383 \| \| NMI \| 4.96E+02 \| 2.03E-04 \| 0.01727339 \| 1.81E+00 \| 1.81023954 \| \| TRAM2 \| 8.71E+01 \| 7.45E-04 \| 0.02646243 \| 1.81E+00 \| 1.81103835 \| \| NT5C3A \| 9.12E+02 \| 2.44E-03 \| 0.0410063 \| 1.81E+00 \| 1.81173084 \| \| MSRB2 \| 6.38E+01 \| 1.10E-03 \| 0.030773 \| 1.82E+00 \| 1.82143224 \| \| TRIM5 \| 3.32E+02 \| 4.14E-03 \| 0.0507589 \| 1.83E+00 \| 1.82964103 \| \| PSME2 \| 1.83E+02 \| 3.26E-03 \| 0.04541638 \| 1.84E+00 \| 1.83623474 \| \| TMEM229B \| 9.45E+01 \| 4.48E-03 \| 0.05226789 \| 1.85E+00 \| 1.85118095 \| \| MMP9 \| 1.33E+03 \| 4.56E-03 \| 0.05231298 \| 1.87E+00 \| 1.86881794 \| \| CCND2 \| 8.48E+02 \| 1.71E-04 \| 0.01655051 \| 1.89E+00 \| 1.89488826 \| \| LINC01138 \| 9.53E+01 \| 2.16E-04 \| 0.01748455 \| 1.90E+00 \| 1.89579048 \| \| ZNF595 \| 3.68E+01 \| 1.85E-03 \| 0.03626429 \| 1.90E+00 \| 1.89938874 \| \| H2AC17 \| 1.81E+02 \| 3.39E-03 \| 0.04624744 \| 1.90E+00 \| 1.90077342 \| \| MANF \| 4.61E+01 \| 2.08E-03 \| 0.03820965 \| 1.90E+00 \| 1.90361551 \| \| JUN \| 6.39E+01 \| 1.10E-03 \| 0.030773 \| 1.91E+00 \| 1.90628555 \| \| FEN1 \| 1.89E+01 \| 4.94E-03 \| 0.0534573 \| 1.93E+00 \| 1.93063282 \| \| TRIM22 \| 2.71E+03 \| 3.20E-03 \| 0.04504367 \| 1.95E+00 \| 1.95318804 \| \| TUFT1 \| 3.24E+01 \| 1.06E-03 \| 0.03041505 \| 1.97E+00 \| 1.96529125 \| \| CD38 \| 1.41E+02 \| 2.77E-03 \| 0.04307144 \| 1.98E+00 \| 1.97528157 \| \| FOXM1 \| 1.52E+01 \| 3.11E-03 \| 0.04451452 \| 2.01E+00 \| 2.00749814 \| \| BTN3A1 \| 7.84E+02 \| 9.54E-04 \| 0.02931861 \| 2.02E+00 \| 2.01807548 \| \| PARP9 \| 2.01E+03 \| 1.32E-03 \| 0.0334893 \| 2.02E+00 \| 2.01849396 \| \| DTX3L \| 1.80E+03 \| 4.39E-04 \| 0.02174576 \| 2.03E+00 \| 2.02808526 \| \| MCM2 \| 3.37E+01 \| 3.52E-03 \| 0.04679372 \| 2.03E+00 \| 2.03487972 \| \| UHRF1 \| 1.82E+01 \| 2.10E-03 \| 0.03828352 \| 2.07E+00 \| 2.06502016 \| \| SAMD9 \| 2.69E+03 \| 1.68E-03 \| 0.0354207 \| 2.07E+00 \| 2.0689776 \| \| ACP6 \| 8.24E+01 \| 5.95E-04 \| 0.02368492 \| 2.08E+00 \| 2.07533513 \| \| TOP2A \| 8.67E+01 \| 1.23E-03 \| 0.03244251 \| 2.08E+00 \| 2.07849572 \| \| PARP11 \| 2.22E+02 \| 2.09E-03 \| 0.03828352 \| 2.14E+00 \| 2.13645739 \| \| TPX2 \| 9.59E+01 \| 3.65E-03 \| 0.04754042 \| 2.15E+00 \| 2.14616499 \| \| PSAT1 \| 1.68E+01 \| 3.96E-03 \| 0.04955909 \| 2.16E+00 \| 2.16336445 \| \| TAP1 \| 1.10E+03 \| 4.39E-03 \| 0.05197104 \| 2.16E+00 \| 2.16413963 \| \| PSMB9 \| 2.60E+02 \| 8.89E-04 \| 0.02862553 \| 2.16E+00 \| 2.16446086 \| \| FRY-AS1 \| 8.09E+00 \| 3.79E-03 \| 0.04843566 \| 2.17E+00 \| 2.17021084 \| \| SHISA5 \| 1.21E+03 \| 3.53E-04 \| 0.02032055 \| 2.17E+00 \| 2.17463066 \| \| LOC100419583 \| 3.36E+02 \| 4.19E-05 \| 0.01358748 \| 2.18E+00 \| 2.17991849 \| \| ESCO2 \| 1.19E+01 \| 2.10E-03 \| 0.03828352 \| 2.18E+00 \| 2.18318333 \| \| TOX \| 2.82E+01 \| 2.99E-03 \| 0.04383474 \| 2.19E+00 \| 2.19285874 \| \| TXLNB \| 2.17E+01 \| 4.98E-03 \| 0.05366228 \| 2.20E+00 \| 2.19550735 \| \| FADS2 \| 7.01E+01 \| 4.80E-03 \| 0.05307894 \| 2.21E+00 \| 2.20661294 \| \| H3C15 \| 2.91E+01 \| 3.42E-03 \| 0.04632133 \| 2.21E+00 \| 2.20783831 \| \| H3C14 \| 2.91E+01 \| 3.42E-03 \| 0.04632133 \| 2.21E+00 \| 2.20783831 \| \| ZNF496 \| 5.74E+01 \| 3.82E-03 \| 0.04859009 \| 2.25E+00 \| 2.24770649 \| \| H2AC12 \| 5.71E+01 \| 2.92E-03 \| 0.0434845 \| 2.25E+00 \| 2.24834968 \| \| SYNJ2 \| 1.15E+02 \| 2.76E-03 \| 0.0430014 \| 2.25E+00 \| 2.25287035 \| \| KLHDC8B \| 5.16E+01 \| 4.71E-03 \| 0.05301601 \| 2.26E+00 \| 2.25749096 \| \| BTN3A3 \| 2.91E+02 \| 4.37E-04 \| 0.02174045 \| 2.26E+00 \| 2.26492909 \| \| IFITM1 \| 1.07E+03 \| 1.42E-03 \| 0.03422635 \| 2.27E+00 \| 2.27190264 \| \| ZNF714 \| 3.79E+01 \| 2.27E-04 \| 0.01796904 \| 2.27E+00 \| 2.2745324 \| \| H3C7 \| 4.64E+01 \| 2.30E-03 \| 0.03980839 \| 2.29E+00 \| 2.28618288 \| \| SRM \| 2.13E+01 \| 2.49E-03 \| 0.04130559 \| 2.29E+00 \| 2.28832443 \| \| PTTG1 \| 4.56E+01 \| 1.28E-03 \| 0.03293681 \| 2.29E+00 \| 2.29293807 \| \| PARP12 \| 3.01E+02 \| 1.61E-03 \| 0.03512358 \| 2.32E+00 \| 2.3185859 \| \| TTK \| 2.69E+01 \| 4.11E-03 \| 0.05064464 \| 2.33E+00 \| 2.3303206 \| \| GTSE1 \| 8.79E+00 \| 3.24E-03 \| 0.04533263 \| 2.35E+00 \| 2.35102366 \| \| SLAMF7 \| 1.15E+02 \| 1.21E-03 \| 0.03218986 \| 2.35E+00 \| 2.35145198 \| \| TK1 \| 8.03E+00 \| 1.15E-03 \| 0.03112937 \| 2.38E+00 \| 2.37669209 \| \| FBXO6 \| 6.17E+01 \| 3.79E-03 \| 0.04843566 \| 2.38E+00 \| 2.37801046 \| \| PARP14 \| 4.12E+03 \| 3.51E-03 \| 0.04678679 \| 2.41E+00 \| 2.40729885 \| \| REXO5 \| 1.56E+01 \| 4.74E-03 \| 0.05304222 \| 2.41E+00 \| 2.4094362 \| \| CCNA2 \| 2.97E+01 \| 4.55E-04 \| 0.02201345 \| 2.42E+00 \| 2.42023634 \| \| KIFC1 \| 9.56E+00 \| 1.00E-03 \| 0.03013087 \| 2.43E+00 \| 2.42525996 \| \| AURKB \| 6.03E+00 \| 2.31E-03 \| 0.03980839 \| 2.45E+00 \| 2.44911913 \| \| TROAP \| 4.71E+00 \| 4.18E-03 \| 0.05096978 \| 2.46E+00 \| 2.46088974 \| \| KIAA1958 \| 2.28E+02 \| 9.00E-04 \| 0.02864918 \| 2.46E+00 \| 2.46423078 \| \| SP140 \| 2.16E+02 \| 2.34E-03 \| 0.04017454 \| 2.46E+00 \| 2.46482711 \| \| NRIR \| 2.60E+01 \| 4.28E-03 \| 0.05137377 \| 2.47E+00 \| 2.47247608 \| \| JUP \| 1.01E+02 \| 3.21E-03 \| 0.0450618 \| 2.48E+00 \| 2.47871006 \| \| C21orf58 \| 1.51E+01 \| 3.90E-03 \| 0.04910116 \| 2.50E+00 \| 2.50068835 \| \| BISPR \| 7.11E+01 \| 9.57E-04 \| 0.0293236 \| 2.51E+00 \| 2.51263098 \| \| DDX58 \| 1.44E+03 \| 2.13E-04 \| 0.01742995 \| 2.52E+00 \| 2.52247003 \| \| CDC45 \| 4.09E+00 \| 4.09E-03 \| 0.05046684 \| 2.53E+00 \| 2.52659992 \| \| RNF208 \| 4.47E+00 \| 4.17E-03 \| 0.0509016 \| 2.53E+00 \| 2.52997437 \| \| NET1 \| 8.15E+01 \| 4.91E-03 \| 0.05331697 \| 2.58E+00 \| 2.58035153 \| \| ORC1 \| 1.13E+01 \| 2.31E-03 \| 0.03980839 \| 2.59E+00 \| 2.58595089 \| \| GALM \| 7.73E+01 \| 5.61E-04 \| 0.02281591 \| 2.59E+00 \| 2.58966132 \| \| ITM2C \| 7.61E+01 \| 4.10E-04 \| 0.0212803 \| 2.60E+00 \| 2.59517494 \| \| STAT1 \| 4.04E+03 \| 1.43E-03 \| 0.03422635 \| 2.62E+00 \| 2.61546643 \| \| UBE2L6 \| 5.61E+02 \| 2.97E-03 \| 0.0437064 \| 2.63E+00 \| 2.62853145 \| \| SEPT5-GP1BB \| 5.50E+01 \| 1.53E-03 \| 0.03462625 \| 2.64E+00 \| 2.6381001 \| \| NDC80 \| 3.03E+01 \| 3.03E-04 \| 0.01914308 \| 2.66E+00 \| 2.65548599 \| \| ARL17B \| 5.65E+01 \| 1.27E-03 \| 0.03293681 \| 2.66E+00 \| 2.65735537 \| \| GFI1B \| 1.49E+02 \| 9.10E-04 \| 0.02864918 \| 2.66E+00 \| 2.66332451 \| \| CHEK1 \| 2.01E+01 \| 1.74E-04 \| 0.01672332 \| 2.67E+00 \| 2.66745809 \| \| AIM2 \| 3.21E+02 \| 6.76E-04 \| 0.02505425 \| 2.69E+00 \| 2.69172001 \| \| GBP3 \| 3.84E+02 \| 2.63E-03 \| 0.04255273 \| 2.71E+00 \| 2.71132853 \| \| H2BC9 \| 1.37E+02 \| 2.23E-05 \| 0.0131811 \| 2.72E+00 \| 2.72003049 \| \| NME1 \| 1.90E+01 \| 4.53E-04 \| 0.02201345 \| 2.75E+00 \| 2.75468938 \| \| GTSF1 \| 5.48E+00 \| 4.80E-03 \| 0.05307894 \| 2.77E+00 \| 2.76839771 \| \| DUSP5 \| 3.55E+01 \| 1.82E-03 \| 0.03592144 \| 2.77E+00 \| 2.77394904 \| \| ZCCHC2 \| 9.12E+02 \| 6.58E-05 \| 0.01358748 \| 2.79E+00 \| 2.79041328 \| \| SPAG5 \| 1.82E+01 \| 2.52E-03 \| 0.04154566 \| 2.80E+00 \| 2.79790766 \| \| SAMD9L \| 2.84E+03 \| 2.68E-03 \| 0.04281263 \| 2.85E+00 \| 2.84919039 \| \| KIF2C \| 8.82E+00 \| 1.34E-03 \| 0.03361727 \| 2.89E+00 \| 2.88982862 \| \| H1-5 \| 2.94E+02 \| 1.55E-04 \| 0.01622824 \| 2.91E+00 \| 2.90655395 \| \| H3C8 \| 4.76E+01 \| 7.04E-04 \| 0.02541308 \| 2.92E+00 \| 2.92278118 \| \| E2F8 \| 9.43E+00 \| 1.92E-03 \| 0.03697811 \| 2.92E+00 \| 2.92391172 \| \| IFI35 \| 1.29E+02 \| 5.66E-04 \| 0.02290154 \| 2.92E+00 \| 2.92449078 \| \| EGFL7 \| 1.24E+01 \| 1.40E-03 \| 0.03422635 \| 2.93E+00 \| 2.93276856 \| \| KLHDC7B \| 2.33E+01 \| 1.48E-03 \| 0.03434026 \| 2.93E+00 \| 2.93277208 \| \| CDCA5 \| 7.37E+00 \| 1.98E-03 \| 0.03723394 \| 2.94E+00 \| 2.93746997 \| \| H3C2 \| 9.50E+01 \| 2.30E-04 \| 0.01796904 \| 2.95E+00 \| 2.94913697 \| \| IFIT5 \| 1.09E+03 \| 2.55E-04 \| 0.01800472 \| 2.95E+00 \| 2.95267698 \| \| IRF7 \| 1.89E+02 \| 3.04E-03 \| 0.04404095 \| 2.96E+00 \| 2.95949897 \| \| H2BC14 \| 7.43E+01 \| 4.20E-04 \| 0.02154268 \| 2.98E+00 \| 2.98403224 \| \| GNAO1 \| 1.50E+01 \| 8.58E-04 \| 0.02849752 \| 3.01E+00 \| 3.00574403 \| \| H2AC14 \| 9.52E+01 \| 5.14E-04 \| 0.02251803 \| 3.03E+00 \| 3.02789001 \| \| APOL6 \| 2.99E+03 \| 1.94E-03 \| 0.03702835 \| 3.04E+00 \| 3.03820794 \| \| SLC27A2 \| 5.28E+00 \| 5.05E-03 \| 0.05394896 \| 3.06E+00 \| 3.05607364 \| \| NEK2 \| 7.39E+00 \| 1.58E-03 \| 0.03496769 \| 3.09E+00 \| 3.09017713 \| \| MYL9 \| 4.34E+02 \| 7.48E-04 \| 0.02646243 \| 3.10E+00 \| 3.0999494 \| \| HASPIN \| 6.30E+00 \| 1.97E-03 \| 0.03723394 \| 3.10E+00 \| 3.10038194 \| \| IFIH1 \| 7.33E+02 \| 1.04E-03 \| 0.03034051 \| 3.21E+00 \| 3.20879512 \| \| PCLAF \| 1.02E+01 \| 1.64E-03 \| 0.03532746 \| 3.22E+00 \| 3.21572293 \| \| WDR62 \| 3.39E+00 \| 2.99E-03 \| 0.04383474 \| 3.22E+00 \| 3.21684278 \| \| PTX3 \| 1.08E+01 \| 2.84E-03 \| 0.0431483 \| 3.23E+00 \| 3.23102328 \| \| SLFN5 \| 1.21E+03 \| 1.18E-04 \| 0.01508103 \| 3.25E+00 \| 3.24795731 \| \| OAS1 \| 9.61E+02 \| 2.65E-04 \| 0.01810876 \| 3.29E+00 \| 3.28997541 \| \| MTSS2 \| 7.16E+00 \| 3.02E-03 \| 0.04401166 \| 3.31E+00 \| 3.30926977 \| \| TYMS \| 2.46E+01 \| 6.87E-04 \| 0.0250927 \| 3.35E+00 \| 3.34982566 \| \| TICRR \| 8.09E+00 \| 1.67E-03 \| 0.03534598 \| 3.36E+00 \| 3.35768172 \| \| MYBL2 \| 5.81E+01 \| 1.99E-04 \| 0.01723618 \| 3.36E+00 \| 3.36004142 \| \| DNM1 \| 1.24E+01 \| 3.38E-03 \| 0.0462197 \| 3.36E+00 \| 3.36349334 \| \| VIL1 \| 3.73E+01 \| 4.12E-03 \| 0.05066207 \| 3.37E+00 \| 3.37392387 \| \| CEP55 \| 1.16E+01 \| 1.32E-03 \| 0.03349918 \| 3.38E+00 \| 3.37965017 \| \| TNFSF15 \| 1.46E+01 \| 4.39E-03 \| 0.05197104 \| 3.43E+00 \| 3.42982387 \| \| INSYN2B \| 4.57E+00 \| 3.69E-03 \| 0.04772117 \| 3.45E+00 \| 3.45103975 \| \| CCNE1 \| 5.05E+00 \| 1.73E-03 \| 0.03557815 \| 3.47E+00 \| 3.47000785 \| \| HJURP \| 1.48E+01 \| 1.06E-03 \| 0.03041505 \| 3.49E+00 \| 3.48814605 \| \| GCSH \| 5.22E+00 \| 1.81E-03 \| 0.03592144 \| 3.54E+00 \| 3.53613851 \| \| STMN1 \| 4.77E+01 \| 4.37E-05 \| 0.01358748 \| 3.54E+00 \| 3.5389699 \| \| EIF2AK2 \| 2.57E+03 \| 4.59E-06 \| 0.00733564 \| 3.60E+00 \| 3.59691491 \| \| DHRS9 \| 3.11E+02 \| 1.20E-04 \| 0.01508103 \| 3.61E+00 \| 3.61161438 \| \| KIF18B \| 6.28E+00 \| 4.85E-04 \| 0.0222881 \| 3.64E+00 \| 3.64075501 \| \| GINS2 \| 5.62E+00 \| 5.36E-04 \| 0.02271088 \| 3.66E+00 \| 3.65624594 \| \| OAS2 \| 2.08E+03 \| 2.56E-04 \| 0.01800472 \| 3.68E+00 \| 3.67548738 \| \| LOC108783645 \| 3.36E+01 \| 4.10E-05 \| 0.01358748 \| 3.69E+00 \| 3.68816267 \| \| CRACD \| 3.16E+01 \| 1.54E-03 \| 0.03476457 \| 3.71E+00 \| 3.71321614 \| \| AUNIP \| 3.23E+00 \| 4.26E-03 \| 0.05129215 \| 3.73E+00 \| 3.7260049 \| \| H2BC17 \| 1.45E+02 \| 3.93E-06 \| 0.00733564 \| 3.77E+00 \| 3.76983191 \| \| GZMB \| 5.68E+01 \| 3.83E-04 \| 0.02090486 \| 3.80E+00 \| 3.79996726 \| \| H3C3 \| 8.68E+01 \| 6.88E-05 \| 0.01358748 \| 3.87E+00 \| 3.8670509 \| \| CCNF \| 1.48E+01 \| 6.28E-05 \| 0.01358748 \| 3.87E+00 \| 3.86725794 \| \| NPIPB13 \| 3.62E+00 \| 4.54E-03 \| 0.05231298 \| 3.89E+00 \| 3.89305077 \| \| TMEM272 \| 1.86E+02 \| 1.21E-04 \| 0.01508103 \| 3.91E+00 \| 3.90643416 \| \| HERC6 \| 1.82E+02 \| 4.90E-05 \| 0.01358748 \| 3.92E+00 \| 3.91715277 \| \| LINC01679 \| 2.57E+00 \| 1.40E-03 \| 0.03422635 \| 3.93E+00 \| 3.93245027 \| \| RACGAP1P \| 1.96E+00 \| 2.64E-03 \| 0.04256329 \| 3.94E+00 \| 3.94237027 \| \| MZB1 \| 3.38E+01 \| 2.93E-03 \| 0.04348498 \| 3.97E+00 \| 3.97445337 \| \| KIF20A \| 6.97E+00 \| 1.03E-03 \| 0.03031049 \| 3.98E+00 \| 3.98399843 \| \| PCP2 \| 1.70E+00 \| 2.85E-03 \| 0.0431483 \| 4.02E+00 \| 4.02172275 \| \| XAF1 \| 8.00E+02 \| 4.82E-04 \| 0.0222881 \| 4.14E+00 \| 4.14259164 \| \| RRM2 \| 8.78E+01 \| 3.48E-05 \| 0.01358748 \| 4.21E+00 \| 4.21004487 \| \| ZNF684 \| 2.94E+01 \| 3.21E-04 \| 0.0194704 \| 4.22E+00 \| 4.22347362 \| \| LY6E \| 6.61E+02 \| 6.73E-05 \| 0.01358748 \| 4.31E+00 \| 4.30934414 \| \| ESPL1 \| 6.22E+00 \| 7.97E-04 \| 0.02766819 \| 4.38E+00 \| 4.37590656 \| \| OASL \| 2.60E+02 \| 7.99E-05 \| 0.01392625 \| 4.47E+00 \| 4.46802385 \| \| DDX60 \| 1.37E+03 \| 8.49E-05 \| 0.01407615 \| 4.52E+00 \| 4.51526804 \| \| BIRC5 \| 1.08E+01 \| 6.68E-04 \| 0.02505425 \| 4.58E+00 \| 4.58342594 \| \| SAMD14 \| 4.81E+01 \| 5.07E-03 \| 0.05411025 \| 4.61E+00 \| 4.61099561 \| \| LAG3 \| 7.60E+00 \| 1.01E-03 \| 0.03024167 \| 4.70E+00 \| 4.70445757 \| \| CMPK2 \| 7.99E+02 \| 1.87E-04 \| 0.01718472 \| 4.78E+00 \| 4.77565639 \| \| HERC5 \| 5.78E+02 \| 1.34E-04 \| 0.01562712 \| 4.82E+00 \| 4.82212156 \| \| RTP4 \| 1.30E+02 \| 5.93E-05 \| 0.01358748 \| 4.88E+00 \| 4.87622467 \| \| TNFRSF17 \| 9.63E+00 \| 2.11E-03 \| 0.0382945 \| 4.93E+00 \| 4.92722859 \| \| SORD2P \| 4.56E+00 \| 6.49E-04 \| 0.02457212 \| 5.19E+00 \| 5.19233291 \| \| GBP1 \| 2.12E+03 \| 1.42E-03 \| 0.03422635 \| 5.29E+00 \| 5.29465977 \| \| PHF11 \| 8.71E+01 \| 1.03E-03 \| 0.03031049 \| 5.33E+00 \| 5.33287345 \| \| IFITM3 \| 2.40E+03 \| 1.16E-05 \| 0.01073864 \| 5.35E+00 \| 5.34921219 \| \| SDC1 \| 4.17E+00 \| 4.55E-03 \| 0.05231298 \| 5.38E+00 \| 5.37807452 \| \| IFIT2 \| 3.77E+03 \| 4.50E-05 \| 0.01358748 \| 5.41E+00 \| 5.40685321 \| \| ALDH1L2 \| 8.64E+00 \| 1.52E-03 \| 0.03454272 \| 5.41E+00 \| 5.41039703 \| \| ZWINT \| 6.73E+00 \| 4.28E-04 \| 0.02156426 \| 5.44E+00 \| 5.43837103 \| \| CDC25A \| 8.68E+00 \| 3.31E-04 \| 0.01980958 \| 5.54E+00 \| 5.53578677 \| \| EPSTI1 \| 7.32E+02 \| 8.70E-05 \| 0.01415534 \| 5.71E+00 \| 5.71136343 \| \| LGALS3BP \| 1.05E+02 \| 4.64E-04 \| 0.02201345 \| 5.81E+00 \| 5.80617087 \| \| CDC20 \| 1.14E+01 \| 3.81E-04 \| 0.02090486 \| 5.92E+00 \| 5.91640231 \| \| MX1 \| 1.51E+03 \| 4.86E-05 \| 0.01358748 \| 6.14E+00 \| 6.13908851 \| \| E2F7 \| 5.58E+00 \| 4.23E-04 \| 0.02156426 \| 6.15E+00 \| 6.14883571 \| \| SERPING1 \| 2.55E+02 \| 1.29E-03 \| 0.03293681 \| 6.49E+00 \| 6.48793773 \| \| CARD17 \| 1.76E+01 \| 1.37E-04 \| 0.01562712 \| 6.49E+00 \| 6.49376558 \| \| LINC02574 \| 9.93E-01 \| 2.70E-04 \| 0.01826046 \| 7.00E+00 \| 6.99757036 \| \| GBP6 \| 4.03E+01 \| 1.37E-03 \| 0.03405687 \| 7.13E+00 \| 7.13492028 \| \| IFI44 \| 9.79E+02 \| 6.17E-05 \| 0.01358748 \| 7.17E+00 \| 7.16713638 \| \| ERCC6L \| 4.02E+00 \| 4.11E-03 \| 0.05064464 \| 7.18E+00 \| 7.18028304 \| \| OAS3 \| 2.38E+03 \| 6.88E-05 \| 0.01358748 \| 7.29E+00 \| 7.29386857 \| \| PDZD2 \| 8.84E+00 \| 4.51E-03 \| 0.05227731 \| 7.34E+00 \| 7.33700263 \| \| IFI6 \| 3.47E+02 \| 1.45E-05 \| 0.01073864 \| 7.38E+00 \| 7.37761708 \| \| EXOC3L1 \| 1.66E+00 \| 1.63E-03 \| 0.03523186 \| 7.56E+00 \| 7.56101036 \| \| SPATS2L \| 1.16E+02 \| 9.25E-05 \| 0.01473715 \| 7.62E+00 \| 7.61692052 \| \| PLAAT2 \| 1.59E+00 \| 3.67E-03 \| 0.04762297 \| 7.95E+00 \| 7.95120025 \| \| RSAD2 \| 2.47E+03 \| 2.48E-04 \| 0.01800472 \| 8.04E+00 \| 8.03845098 \| \| IFIT3 \| 2.63E+03 \| 3.34E-05 \| 0.01358748 \| 8.34E+00 \| 8.33506387 \| \| LINC00908 \| 2.05E+00 \| 4.81E-03 \| 0.05307894 \| 8.43E+00 \| 8.43470803 \| \| CCDC140 \| 5.94E-01 \| 3.88E-03 \| 0.04895559 \| 8.61E+00 \| 8.60613289 \| \| USP18 \| 7.32E+01 \| 5.05E-05 \| 0.01358748 \| 8.69E+00 \| 8.69260448 \| \| KCNN3 \| 6.25E+00 \| 3.94E-03 \| 0.04944595 \| 8.85E+00 \| 8.85015323 \| \| KY \| 9.58E+00 \| 3.83E-03 \| 0.04859009 \| 8.91E+00 \| 8.90856261 \| \| CABLES1 \| 1.48E+00 \| 2.46E-03 \| 0.041125 \| 8.99E+00 \| 8.99371775 \| \| MUSTN1 \| 1.46E+00 \| 1.43E-03 \| 0.03422635 \| 9.28E+00 \| 9.27925645 \| \| LAMP3 \| 2.14E+01 \| 9.02E-04 \| 0.02864918 \| 9.50E+00 \| 9.49557306 \| \| ANKRD45 \| 1.91E+01 \| 6.88E-04 \| 0.0250927 \| 9.87E+00 \| 9.87164827 \| \| ISG15 \| 2.69E+02 \| 7.39E-05 \| 0.01383902 \| 9.96E+00 \| 9.96236714 \| \| ETV7 \| 7.05E+01 \| 5.27E-04 \| 0.02266365 \| 1.09E+01 \| 10.9176585 \| \| CDT1 \| 4.76E+00 \| 2.78E-03 \| 0.04307144 \| 1.11E+01 \| 11.0956422 \| \| ZBTB32 \| 3.72E+00 \| 9.49E-05 \| 0.01482445 \| 1.12E+01 \| 11.238116 \| \| HESX1 \| 4.73E+00 \| 5.70E-04 \| 0.02292566 \| 1.18E+01 \| 11.8484939 \| \| IFIT1 \| 1.39E+03 \| 6.81E-06 \| 0.00733564 \| 1.20E+01 \| 12.032719 \| \| ADRA2A \| 6.24E+00 \| 2.56E-03 \| 0.04188047 \| 1.25E+01 \| 12.4939272 \| \| KCNK7 \| 8.65E-01 \| 1.44E-03 \| 0.03422635 \| 1.29E+01 \| 12.8774767 \| \| LEFTY2 \| 6.14E-01 \| 2.75E-03 \| 0.04297444 \| 1.34E+01 \| 13.4378237 \| \| IFI44L \| 2.42E+03 \| 1.78E-05 \| 0.0109766 \| 1.56E+01 \| 15.5520679 \| \| SIGLEC1 \| 2.34E+02 \| 1.26E-04 \| 0.01521814 \| 1.57E+01 \| 15.6870852 \| \| CCR10 \| 5.87E-01 \| 3.26E-03 \| 0.04541638 \| 1.59E+01 \| 15.9016186 \| \| CHMP4C \| 1.38E+00 \| 1.98E-03 \| 0.03723394 \| 1.61E+01 \| 16.1144787 \| \| IFI27 \| 7.46E+02 \| 1.50E-04 \| 0.01607971 \| 1.68E+01 \| 16.7964567 \| \| PRRT4 \| 9.69E-01 \| 3.42E-03 \| 0.04632133 \| 1.79E+01 \| 17.9423245 \| \| CTAGE15 \| 1.32E+00 \| 1.56E-03 \| 0.03496769 \| 1.86E+01 \| 18.5774033 \| \| SERF2-C15ORF63 \| 1.75E+00 \| 8.04E-04 \| 0.02766819 \| 2.00E+01 \| 19.9853913 \| \| NDUFC2-KCTD14 \| 2.14E+00 \| 1.08E-03 \| 0.03063671 \| 2.13E+01 \| 21.2613596 \| \| SPDYC \| 4.32E+00 \| 5.38E-04 \| 0.02271088 \| 2.40E+01 \| 24.0017293 \| \| EME1 \| 1.12E+00 \| 2.35E-04 \| 0.01796904 \| 2.43E+01 \| 24.2731256 \| \| SCN5A \| 3.14E+00 \| 3.57E-03 \| 0.04724039 \| 2.83E+01 \| 28.3483921 \| \| C3orf70 \| 1.13E+00 \| 3.79E-04 \| 0.02090486 \| 2.92E+01 \| 29.1892336 \| \| MET \| 2.04E+00 \| 3.65E-04 \| 0.02063449 \| 3.31E+01 \| 33.0580449 \| \| GRTP1 \| 1.42E+00 \| 8.13E-04 \| 0.02766819 \| 3.36E+01 \| 33.5655256 \| \| MTARC2 \| 1.73E+00 \| 3.39E-04 \| 0.01980958 \| 3.43E+01 \| 34.3348491 \| \| LIF \| 8.76E-01 \| 1.12E-03 \| 0.0310461 \| 3.50E+01 \| 34.9869599 \| \| SLCO4A1 \| 1.77E+00 \| 5.09E-07 \| 0.00251186 \| 3.70E+01 \| 37.0062745 \| \| KRT79 \| 2.44E+00 \| 2.81E-03 \| 0.0431483 \| 3.99E+01 \| 39.9174337 \| \| TP73 \| 1.23E+00 \| 1.60E-04 \| 0.01638822 \| 4.84E+01 \| 48.425548 \| \| LOC102724708 \| 1.83E+00 \| 1.63E-04 \| 0.01638822 \| 4.87E+01 \| 48.7192846 \| \| CD34 \| 1.64E+00 \| 6.75E-04 \| 0.02505425 \| 5.32E+01 \| 53.2445082 \| \| OTOF \| 4.42E+01 \| 4.78E-04 \| 0.02227986 \| 5.70E+01 \| 56.9972409 \| \| PCDHGA12 \| 1.14E+00 \| 1.94E-03 \| 0.03703901 \| 5.95E+01 \| 59.5187853 \| \| MOCS1 \| 1.55E+00 \| 3.91E-04 \| 0.02096464 \| 6.86E+01 \| 68.6393859 \| \| ARHGEF34P \| 5.47E+00 \| 2.90E-03 \| 0.04340555 \| 8.87E+01 \| 88.7199592 \| \| UCHL1 \| 2.76E+00 \| 3.49E-04 \| 0.02021139 \| 2.42E+02 \| 242.099098 \| \| PPM1K-DT \| 6.72E-01 \| 3.89E-03 \| 0.04903783 \| 9.60E+02 \| 959.500955 \| |
| --- | --- | --- | --- | --- | --- | --- | --- | --- | --- | --- | --- | --- | --- | --- | --- | --- | --- | --- | --- | --- | --- | --- | --- | --- | --- | --- | --- | --- | --- | --- | --- | --- | --- | --- | --- | --- | --- | --- | --- | --- | --- | --- | --- | --- | --- | --- | --- | --- | --- | --- | --- | --- | --- | --- | --- | --- | --- | --- | --- | --- | --- | --- | --- | --- | --- | --- | --- | --- | --- | --- | --- | --- | --- | --- | --- | --- | --- | --- | --- | --- | --- | --- | --- | --- | --- | --- | --- | --- | --- | --- | --- | --- | --- | --- | --- | --- | --- | --- | --- | --- | --- | --- | --- | --- | --- | --- | --- | --- | --- | --- | --- | --- | --- | --- | --- | --- | --- | --- | --- | --- | --- | --- | --- | --- | --- | --- | --- | --- | --- | --- | --- | --- | --- | --- | --- | --- | --- | --- | --- | --- | --- | --- | --- | --- | --- | --- | --- | --- | --- | --- | --- | --- | --- | --- | --- | --- | --- | --- | --- | --- | --- | --- | --- | --- | --- | --- | --- | --- | --- | --- | --- | --- | --- | --- | --- | --- | --- | --- | --- | --- | --- | --- | --- | --- | --- | --- | --- | --- | --- | --- | --- | --- | --- | --- | --- | --- | --- | --- | --- | --- | --- | --- | --- | --- | --- | --- | --- | --- | --- | --- | --- | --- | --- | --- | --- | --- | --- | --- | --- | --- | --- | --- | --- | --- | --- | --- | --- | --- | --- | --- | --- | --- | --- | --- | --- | --- | --- | --- | --- | --- | --- | --- | --- | --- | --- | --- | --- | --- | --- | --- | --- | --- | --- | --- | --- | --- | --- | --- | --- | --- | --- | --- | --- | --- | --- | --- | --- | --- | --- | --- | --- | --- | --- | --- | --- | --- | --- | --- | --- | --- | --- | --- | --- | --- | --- | --- | --- | --- | --- | --- | --- | --- | --- | --- | --- | --- | --- | --- | --- | --- | --- | --- | --- | --- | --- | --- | --- | --- | --- | --- | --- | --- | --- | --- | --- | --- | --- | --- | --- | --- | --- | --- | --- | --- | --- | --- | --- | --- | --- | --- | --- | --- | --- | --- | --- | --- | --- | --- | --- | --- | --- | --- | --- | --- | --- | --- | --- | --- | --- | --- | --- | --- | --- | --- | --- | --- | --- | --- | --- | --- | --- | --- | --- | --- | --- | --- | --- | --- | --- | --- | --- | --- | --- | --- | --- | --- | --- | --- | --- | --- | --- | --- | --- | --- | --- | --- | --- | --- | --- | --- | --- | --- | --- | --- | --- | --- | --- | --- | --- | --- | --- | --- | --- | --- | --- | --- | --- | --- | --- | --- | --- | --- | --- | --- | --- | --- | --- | --- | --- | --- | --- | --- | --- | --- | --- | --- | --- | --- | --- | --- | --- | --- | --- | --- | --- | --- | --- | --- | --- | --- | --- | --- | --- | --- | --- | --- | --- | --- | --- | --- | --- | --- | --- | --- | --- | --- | --- | --- | --- | --- | --- | --- | --- | --- | --- | --- | --- | --- | --- | --- | --- | --- | --- | --- | --- | --- | --- | --- | --- | --- | --- | --- | --- | --- | --- | --- | --- | --- | --- | --- | --- | --- | --- | --- | --- | --- | --- | --- | --- | --- | --- | --- | --- | --- | --- | --- | --- | --- | --- | --- | --- | --- | --- | --- | --- | --- | --- | --- | --- | --- | --- | --- | --- | --- | --- | --- | --- | --- | --- | --- | --- | --- | --- | --- | --- | --- | --- | --- | --- | --- | --- | --- | --- | --- | --- | --- | --- | --- | --- | --- | --- | --- | --- | --- | --- | --- | --- | --- | --- | --- | --- | --- | --- | --- | --- | --- | --- | --- | --- | --- | --- | --- | --- | --- | --- | --- | --- | --- | --- | --- | --- | --- | --- | --- | --- | --- | --- | --- | --- | --- | --- | --- | --- | --- | --- | --- | --- | --- | --- | --- | --- | --- | --- | --- | --- | --- | --- | --- | --- | --- | --- | --- | --- | --- | --- | --- | --- | --- | --- | --- | --- | --- | --- | --- | --- | --- | --- | --- | --- | --- | --- | --- | --- | --- | --- | --- | --- | --- | --- | --- | --- | --- | --- | --- | --- | --- | --- | --- | --- | --- | --- | --- | --- | --- | --- | --- | --- | --- | --- | --- | --- | --- | --- | --- | --- | --- | --- | --- | --- | --- | --- | --- | --- | --- | --- | --- | --- | --- | --- | --- | --- | --- | --- | --- | --- | --- | --- | --- | --- | --- | --- | --- | --- | --- | --- | --- | --- | --- | --- | --- | --- | --- | --- | --- | --- | --- | --- | --- | --- | --- | --- | --- | --- | --- | --- | --- | --- | --- | --- | --- | --- | --- | --- | --- | --- | --- | --- | --- | --- | --- | --- | --- | --- | --- | --- | --- | --- | --- | --- | --- | --- | --- | --- | --- | --- | --- | --- | --- | --- | --- | --- | --- | --- | --- | --- | --- | --- | --- | --- | --- | --- | --- | --- | --- | --- | --- | --- | --- | --- | --- | --- | --- | --- | --- | --- | --- | --- | --- | --- | --- | --- | --- | --- | --- | --- | --- | --- | --- | --- | --- | --- | --- | --- | --- | --- | --- | --- | --- | --- | --- | --- | --- | --- | --- | --- | --- | --- | --- | --- | --- | --- | --- | --- | --- | --- | --- | --- | --- | --- | --- | --- | --- | --- | --- | --- | --- | --- | --- | --- | --- | --- | --- | --- | --- | --- | --- | --- | --- | --- | --- | --- | --- | --- | --- | --- | --- | --- | --- | --- | --- | --- | --- | --- | --- | --- | --- | --- | --- | --- | --- | --- | --- | --- | --- | --- | --- | --- | --- | --- | --- | --- | --- | --- | --- | --- | --- | --- | --- | --- | --- | --- | --- | --- | --- | --- | --- | --- | --- | --- | --- | --- | --- | --- | --- | --- | --- | --- | --- | --- | --- | --- | --- | --- | --- | --- | --- | --- | --- | --- | --- | --- | --- | --- | --- | --- | --- | --- | --- | --- | --- | --- | --- | --- | --- | --- | --- | --- | --- | --- | --- | --- | --- | --- | --- | --- | --- | --- | --- | --- | --- | --- | --- | --- | --- | --- | --- | --- | --- | --- | --- | --- | --- | --- | --- | --- | --- | --- | --- | --- | --- | --- | --- | --- | --- | --- | --- | --- | --- | --- | --- | --- | --- | --- | --- | --- | --- | --- | --- | --- | --- | --- | --- | --- | --- | --- | --- | --- | --- | --- | --- | --- | --- | --- | --- | --- | --- | --- | --- | --- | --- | --- | --- | --- | --- | --- | --- | --- | --- | --- | --- | --- | --- | --- | --- | --- | --- | --- | --- | --- | --- | --- | --- | --- | --- | --- | --- | --- | --- | --- | --- | --- | --- | --- | --- | --- | --- | --- | --- | --- | --- | --- | --- | --- | --- | --- | --- | --- | --- | --- | --- | --- | --- | --- | --- | --- | --- | --- | --- | --- | --- | --- | --- | --- | --- | --- | --- | --- | --- | --- | --- | --- | --- | --- | --- | --- | --- | --- | --- | --- | --- | --- | --- | --- | --- | --- | --- | --- | --- | --- | --- | --- | --- | --- | --- | --- | --- | --- | --- | --- | --- | --- | --- | --- | --- | --- | --- | --- | --- | --- | --- | --- | --- | --- | --- | --- | --- | --- | --- | --- | --- | --- | --- | --- | --- | --- | --- | --- | --- | --- | --- | --- | --- | --- | --- | --- | --- | --- | --- | --- | --- | --- | --- | --- | --- | --- | --- | --- | --- | --- | --- | --- | --- | --- | --- | --- | --- | --- | --- | --- | --- | --- | --- | --- | --- | --- | --- | --- | --- | --- | --- | --- | --- | --- | --- | --- | --- | --- | --- | --- | --- | --- | --- | --- | --- | --- | --- | --- | --- | --- | --- | --- | --- | --- | --- | --- | --- | --- | --- | --- | --- | --- | --- | --- | --- | --- | --- | --- | --- | --- | --- | --- | --- | --- | --- | --- | --- | --- | --- | --- | --- | --- | --- | --- | --- | --- | --- | --- | --- | --- | --- | --- | --- | --- | --- | --- | --- | --- | --- | --- | --- | --- | --- | --- | --- | --- | --- | --- | --- | --- | --- | --- | --- | --- | --- | --- | --- | --- | --- | --- | --- | --- | --- | --- | --- | --- | --- | --- | --- | --- | --- | --- | --- | --- | --- | --- | --- | --- | --- | --- | --- | --- | --- | --- | --- | --- | --- | --- | --- | --- | --- | --- | --- | --- | --- | --- | --- | --- | --- | --- | --- | --- | --- | --- | --- | --- | --- | --- | --- | --- | --- | --- | --- | --- | --- | --- | --- | --- | --- | --- | --- | --- | --- | --- | --- | --- | --- | --- | --- | --- | --- | --- | --- | --- | --- | --- | --- | --- | --- | --- | --- | --- | --- | --- | --- | --- | --- | --- | --- | --- | --- | --- | --- | --- | --- | --- | --- | --- | --- | --- | --- | --- | --- | --- | --- | --- | --- | --- | --- | --- | --- | --- | --- | --- | --- | --- | --- | --- | --- | --- | --- | --- | --- | --- | --- | --- | --- | --- | --- | --- | --- | --- | --- | --- | --- | --- | --- | --- | --- | --- | --- | --- | --- | --- | --- | --- | --- | --- | --- | --- | --- | --- | --- | --- | --- | --- | --- | --- | --- | --- | --- | --- | --- | --- | --- | --- | --- | --- | --- | --- | --- | --- | --- | --- | --- | --- | --- | --- | --- | --- | --- | --- | --- | --- | --- | --- | --- | --- | --- | --- | --- | --- | --- | --- | --- | --- | --- | --- | --- | --- | --- | --- | --- | --- | --- | --- | --- | --- | --- | --- | --- | --- | --- | --- | --- | --- | --- | --- | --- | --- | --- | --- | --- | --- | --- | --- | --- | --- | --- | --- | --- | --- | --- | --- | --- | --- | --- | --- | --- | --- | --- | --- | --- | --- | --- | --- | --- | --- | --- | --- | --- | --- | --- | --- | --- | --- | --- | --- | --- | --- | --- | --- | --- | --- | --- | --- | --- | --- | --- | --- | --- | --- | --- | --- | --- | --- | --- | --- | --- | --- | --- | --- | --- | --- | --- | --- | --- | --- | --- | --- | --- | --- | --- | --- | --- | --- | --- | --- | --- | --- | --- | --- | --- | --- | --- | --- | --- | --- | --- | --- | --- | --- | --- | --- | --- | --- | --- | --- | --- | --- | --- | --- | --- | --- | --- | --- | --- | --- | --- | --- | --- | --- | --- | --- | --- | --- | --- | --- | --- | --- | --- | --- | --- | --- | --- | --- | --- | --- | --- | --- | --- | --- | --- | --- | --- | --- | --- | --- | --- | --- | --- | --- | --- | --- | --- | --- | --- | --- | --- | --- | --- | --- | --- | --- | --- | --- | --- | --- | --- | --- | --- | --- | --- | --- | --- | --- | --- | --- | --- | --- | --- | --- | --- | --- | --- | --- | --- | --- | --- | --- | --- | --- | --- | --- | --- | --- | --- | --- | --- | --- | --- | --- | --- | --- | --- | --- | --- | --- | --- | --- | --- | --- | --- | --- | --- | --- | --- | --- | --- | --- | --- | --- | --- | --- | --- | --- | --- | --- | --- | --- | --- | --- | --- | --- | --- | --- | --- | --- | --- | --- | --- | --- | --- | --- | --- | --- | --- | --- | --- | --- | --- | --- | --- | --- | --- | --- | --- | --- | --- | --- | --- | --- | --- | --- | --- | --- | --- | --- | --- | --- | --- | --- | --- | --- | --- | --- | --- | --- | --- | --- | --- | --- | --- | --- | --- | --- | --- | --- | --- | --- | --- | --- | --- | --- | --- | --- | --- | --- | --- | --- | --- | --- | --- | --- | --- | --- | --- | --- | --- | --- | --- | --- | --- | --- | --- | --- | --- | --- | --- | --- | --- | --- | --- | --- | --- | --- | --- | --- | --- | --- | --- | --- | --- | --- | --- | --- | --- | --- | --- | --- | --- | --- | --- | --- | --- | --- | --- | --- | --- | --- | --- | --- | --- | --- | --- | --- | --- | --- | --- | --- | --- | --- | --- | --- | --- | --- | --- | --- | --- | --- | --- | --- | --- | --- | --- | --- | --- | --- | --- | --- | --- | --- | --- | --- | --- | --- | --- | --- | --- | --- | --- | --- | --- | --- | --- | --- | --- | --- | --- | --- | --- | --- | --- | --- | --- | --- | --- | --- | --- | --- | --- | --- | --- | --- | --- | --- | --- | --- | --- | --- | --- | --- | --- | --- | --- | --- | --- | --- | --- | --- | --- | --- | --- | --- | --- | --- | --- | --- | --- | --- | --- | --- | --- | --- | --- | --- | --- | --- | --- | --- | --- | --- | --- | --- | --- | --- | --- | --- | --- | --- | --- | --- | --- | --- | --- | --- | --- | --- | --- | --- | --- | --- | --- | --- | --- | --- | --- | --- | --- | --- | --- | --- | --- | --- | --- | --- | --- | --- | --- | --- | --- | --- | --- | --- | --- | --- | --- | --- | --- | --- | --- | --- | --- | --- | --- | --- | --- | --- | --- | --- | --- | --- | --- | --- | --- | --- | --- | --- | --- | --- | --- | --- | --- | --- | --- | --- | --- | --- | --- | --- | --- | --- | --- | --- | --- | --- | --- | --- | --- | --- | --- | --- | --- | --- | --- | --- | --- | --- | --- | --- | --- | --- | --- | --- | --- | --- | --- | --- | --- | --- | --- | --- | --- | --- | --- | --- | --- | --- | --- | --- | --- | --- | --- | --- | --- | --- | --- | --- | --- | --- | --- | --- | --- | --- | --- | --- | --- | --- | --- | --- | --- | --- | --- | --- | --- | --- | --- | --- | --- | --- | --- | --- | --- | --- | --- | --- | --- | --- | --- | --- | --- | --- | --- | --- | --- | --- | --- | --- | --- | --- | --- | --- | --- | --- | --- | --- | --- | --- | --- | --- | --- | --- | --- | --- | --- | --- | --- | --- | --- | --- | --- | --- | --- | --- | --- | --- | --- | --- | --- | --- | --- | --- | --- | --- | --- | --- | --- | --- | --- | --- | --- | --- | --- | --- | --- | --- | --- | --- | --- | --- | --- | --- | --- | --- | --- | --- | --- | --- | --- | --- | --- | --- | --- | --- | --- | --- | --- | --- | --- | --- | --- | --- | --- | --- | --- | --- | --- | --- | --- | --- | --- | --- | --- | --- | --- | --- | --- | --- | --- | --- | --- | --- | --- | --- | --- | --- | --- | --- | --- | --- | --- | --- | --- | --- | --- | --- | --- | --- | --- | --- | --- | --- | --- | --- | --- | --- | --- | --- | --- | --- | --- | --- | --- | --- | --- | --- | --- | --- | --- | --- | --- | --- | --- | --- | --- | --- | --- | --- | --- | --- | --- | --- | --- | --- | --- | --- | --- | --- | --- | --- | --- | --- | --- | --- | --- | --- | --- | --- | --- | --- | --- | --- | --- | --- | --- | --- | --- | --- | --- | --- | --- | --- | --- | --- | --- | --- | --- | --- | --- | --- | --- | --- | --- | --- | --- | --- | --- | --- | --- | --- | --- | --- | --- | --- | --- | --- | --- | --- | --- | --- | --- | --- | --- | --- | --- | --- | --- | --- | --- | --- | --- | --- | --- | --- | --- | --- | --- | --- | --- | --- | --- | --- | --- | --- | --- | --- | --- | --- | --- | --- | --- | --- | --- | --- | --- | --- | --- | --- | --- | --- | --- | --- | --- | --- | --- | --- | --- | --- | --- | --- | --- | --- | --- | --- | --- | --- | --- | --- | --- | --- | --- | --- | --- | --- | --- | --- | --- | --- | --- | --- | --- | --- | --- | --- | --- | --- | --- | --- | --- | --- | --- | --- | --- | --- | --- | --- | --- | --- | --- | --- | --- | --- | --- | --- | --- | --- | --- | --- | --- | --- | --- | --- | --- | --- | --- | --- | --- | --- | --- | --- | --- | --- | --- | --- | --- | --- | --- | --- | --- | --- | --- | --- | --- | --- | --- | --- | --- | --- | --- | --- | --- | --- | --- | --- | --- | --- | --- | --- | --- | --- | --- | --- | --- | --- | --- | --- | --- | --- | --- | --- | --- | --- | --- | --- | --- | --- | --- | --- | --- | --- | --- | --- | --- | --- | --- | --- | --- | --- | --- | --- | --- | --- | --- | --- | --- | --- | --- | --- | --- | --- | --- | --- | --- | --- | --- | --- | --- | --- | --- | --- | --- | --- | --- | --- | --- | --- | --- | --- | --- | --- | --- | --- | --- | --- | --- | --- | --- | --- | --- | --- | --- | --- | --- | --- | --- | --- | --- | --- | --- | --- | --- | --- | --- | --- | --- | --- | --- | --- | --- | --- | --- | --- | --- | --- | --- | --- | --- | --- | --- | --- | --- | --- | --- | --- | --- | --- | --- | --- | --- | --- | --- | --- | --- | --- | --- | --- | --- | --- | --- | --- | --- | --- | --- | --- | --- | --- | --- | --- | --- | --- | --- | --- | --- | --- | --- | --- | --- | --- | --- | --- | --- | --- | --- | --- | --- | --- | --- | --- | --- | --- | --- | --- | --- | --- | --- | --- | --- | --- | --- | --- | --- | --- | --- | --- | --- | --- | --- | --- | --- | --- | --- | --- | --- | --- | --- | --- | --- | --- | --- | --- | --- | --- | --- | --- | --- | --- | --- | --- | --- | --- | --- | --- | --- | --- | --- | --- | --- | --- | --- | --- | --- | --- | --- | --- | --- | --- | --- | --- | --- | --- | --- | --- | --- | --- | --- | --- | --- | --- | --- | --- | --- | --- | --- | --- | --- | --- | --- | --- | --- | --- | --- | --- | --- | --- | --- | --- | --- | --- | --- | --- | --- | --- | --- | --- | --- | --- | --- | --- | --- | --- | --- | --- | --- | --- | --- | --- | --- | --- | --- | --- | --- | --- | --- | --- | --- | --- | --- | --- | --- | --- | --- | --- | --- | --- | --- | --- | --- | --- | --- | --- | --- | --- | --- | --- | --- | --- | --- | --- | --- | --- | --- | --- | --- | --- | --- | --- | --- | --- | --- | --- | --- | --- | --- | --- | --- | --- | --- | --- | --- | --- | --- | --- | --- | --- | --- | --- | --- | --- | --- | --- | --- | --- | --- | --- | --- | --- | --- | --- | --- | --- | --- | --- | --- | --- | --- | --- | --- | --- | --- | --- | --- | --- | --- | --- | --- | --- | --- | --- | --- | --- | --- | --- | --- | --- | --- | --- | --- | --- | --- | --- | --- | --- | --- | --- | --- | --- | --- | --- | --- | --- | --- | --- | --- | --- | --- | --- | --- | --- | --- | --- | --- | --- | --- | --- | --- | --- | --- | --- | --- | --- | --- | --- | --- | --- | --- | --- | --- | --- | --- | --- | --- | --- | --- | --- | --- | --- | --- | --- | --- | --- | --- | --- | --- | --- | --- | --- | --- | --- | --- | --- | --- | --- | --- | --- | --- | --- | --- | --- | --- | --- | --- | --- | --- | --- | --- | --- | --- | --- | --- | --- | --- | --- | --- | --- | --- | --- | --- | --- | --- | --- | --- | --- | --- | --- | --- | --- | --- | --- | --- | --- | --- | --- | --- | --- | --- | --- | --- | --- | --- | --- | --- | --- | --- | --- | --- | --- | --- | --- | --- | --- | --- | --- | --- | --- | --- | --- | --- | --- | --- | --- | --- | --- | --- | --- | --- | --- | --- | --- | --- | --- | --- | --- | --- | --- | --- | --- | --- | --- | --- | --- | --- | --- | --- | --- | --- | --- | --- | --- | --- | --- | --- | --- | --- | --- | --- | --- | --- | --- | --- | --- | --- | --- | --- | --- | --- | --- | --- | --- | --- | --- | --- | --- | --- | --- | --- | --- | --- | --- | --- | --- | --- | --- | --- | --- | --- | --- | --- | --- | --- | --- | --- | --- | --- | --- | --- | --- | --- | --- | --- | --- | --- | --- | --- | --- | --- | --- | --- | --- | --- | --- | --- | --- | --- | --- | --- | --- | --- | --- | --- | --- | --- | --- | --- | --- | --- | --- | --- | --- | --- | --- | --- | --- | --- | --- | --- | --- | --- | --- | --- | --- | --- | --- | --- | --- | --- | --- | --- | --- | --- | --- | --- | --- | --- | --- | --- | --- | --- | --- | --- | --- | --- | --- | --- | --- | --- | --- | --- | --- | --- | --- | --- | --- | --- | --- | --- | --- | --- | --- | --- | --- | --- | --- | --- | --- | --- | --- | --- | --- | --- | --- | --- | --- | --- | --- | --- | --- | --- | --- | --- | --- | --- | --- | --- | --- | --- | --- | --- | --- | --- | --- | --- | --- | --- | --- | --- | --- | --- | --- | --- | --- | --- | --- | --- | --- | --- | --- | --- | --- | --- | --- | --- | --- | --- | --- | --- | --- | --- | --- | --- | --- | --- | --- | --- | --- | --- | --- | --- | --- | --- | --- | --- | --- | --- | --- | --- | --- | --- | --- | --- | --- | --- | --- | --- | --- | --- | --- | --- | --- | --- | --- | --- | --- | --- | --- | --- | --- | --- | --- | --- | --- | --- | --- | --- | --- | --- | --- | --- | --- | --- | --- | --- | --- | --- | --- | --- | --- | --- | --- | --- | --- | --- | --- | --- | --- | --- | --- | --- | --- | --- | --- | --- | --- | --- | --- | --- | --- | --- | --- | --- | --- | --- | --- | --- | --- | --- | --- | --- | --- | --- | --- | --- | --- | --- | --- | --- | --- | --- | --- | --- | --- | --- | --- | --- | --- | --- | --- | --- | --- | --- | --- | --- | --- | --- | --- | --- | --- | --- | --- | --- | --- | --- | --- | --- | --- | --- | --- | --- | --- | --- | --- | --- | --- | --- | --- | --- | --- | --- | --- | --- | --- | --- | --- | --- | --- | --- | --- | --- | --- | --- | --- | --- | --- | --- | --- | --- | --- | --- | --- | --- | --- | --- | --- | --- | --- | --- | --- | --- | --- | --- | --- | --- | --- | --- | --- | --- | --- | --- | --- | --- | --- | --- | --- | --- | --- | --- | --- | --- | --- | --- | --- | --- | --- | --- | --- | --- | --- | --- | --- | --- | --- | --- | --- | --- | --- | --- | --- | --- | --- | --- | --- | --- | --- | --- | --- | --- | --- | --- | --- | --- | --- | --- | --- | --- | --- | --- | --- | --- | --- | --- | --- | --- | --- | --- | --- | --- | --- | --- | --- | --- | --- | --- | --- | --- | --- | --- | --- | --- | --- | --- | --- | --- | --- | --- | --- | --- | --- | --- | --- | --- | --- | --- | --- | --- | --- | --- | --- | --- | --- | --- | --- | --- | --- | --- | --- | --- | --- | --- | --- | --- | --- | --- | --- | --- | --- | --- | --- | --- | --- | --- | --- | --- | --- | --- | --- | --- | --- | --- | --- | --- | --- | --- | --- | --- | --- | --- | --- | --- | --- | --- | --- | --- | --- | --- | --- | --- | --- | --- | --- | --- | --- | --- | --- | --- | --- | --- | --- | --- | --- | --- | --- | --- | --- | --- | --- | --- | --- | --- | --- | --- | --- | --- | --- | --- | --- | --- | --- | --- | --- | --- | --- | --- | --- | --- | --- | --- | --- | --- | --- | --- | --- | --- | --- | --- | --- | --- | --- | --- | --- | --- | --- | --- | --- | --- | --- | --- | --- | --- | --- | --- | --- | --- | --- | --- | --- | --- | --- | --- | --- | --- | --- | --- | --- | --- | --- | --- | --- | --- | --- | --- | --- | --- | --- | --- | --- | --- | --- | --- | --- | --- | --- | --- | --- | --- | --- | --- | --- | --- | --- | --- | --- | --- | --- | --- | --- | --- | --- | --- | --- | --- | --- | --- | --- | --- | --- | --- | --- | --- | --- | --- | --- | --- | --- | --- | --- | --- | --- | --- | --- | --- | --- | --- | --- | --- | --- | --- | --- | --- | --- | --- | --- | --- | --- | --- | --- | --- | --- | --- | --- | --- | --- | --- | --- | --- | --- | --- | --- | --- | --- | --- | --- | --- | --- | --- | --- | --- | --- | --- | --- | --- | --- | --- | --- | --- | --- | --- | --- | --- | --- | --- | --- | --- | --- | --- | --- | --- | --- | --- | --- | --- | --- | --- | --- | --- | --- | --- | --- | --- | --- | --- | --- | --- | --- | --- | --- | --- | --- | --- | --- | --- | --- | --- | --- | --- | --- | --- | --- | --- | --- | --- | --- | --- | --- | --- | --- | --- | --- | --- | --- | --- | --- | --- | --- | --- | --- | --- | --- | --- | --- | --- | --- | --- | --- | --- | --- | --- | --- | --- | --- | --- | --- | --- | --- | --- | --- | --- | --- | --- | --- | --- | --- | --- | --- | --- | --- | --- | --- | --- | --- | --- | --- | --- | --- | --- | --- | --- | --- | --- | --- | --- | --- | --- | --- | --- | --- | --- | --- | --- | --- | --- | --- | --- | --- | --- | --- | --- | --- | --- | --- | --- | --- | --- | --- | --- | --- | --- | --- | --- | --- | --- | --- | --- | --- | --- | --- | --- | --- | --- | --- | --- | --- | --- | --- | --- | --- | --- | --- | --- | --- | --- | --- | --- | --- | --- | --- | --- | --- | --- | --- | --- | --- | --- | --- | --- | --- | --- | --- | --- | --- | --- | --- | --- | --- | --- | --- | --- | --- | --- | --- | --- | --- | --- | --- | --- | --- | --- | --- | --- | --- | --- | --- | --- | --- | --- | --- | --- | --- | --- | --- | --- | --- | --- | --- | --- | --- | --- | --- | --- | --- | --- | --- | --- | --- | --- | --- | --- | --- | --- | --- | --- | --- | --- | --- | --- | --- | --- | --- | --- | --- | --- | --- | --- | --- | --- | --- | --- | --- | --- | --- | --- | --- | --- | --- | --- | --- | --- | --- | --- | --- | --- | --- | --- | --- | --- | --- | --- | --- | --- | --- | --- | --- | --- | --- | --- | --- | --- | --- | --- | --- | --- | --- | --- | --- | --- | --- | --- | --- | --- | --- | --- | --- | --- | --- | --- | --- | --- | --- | --- | --- | --- | --- | --- | --- | --- | --- | --- | --- | --- | --- | --- | --- | --- | --- | --- | --- | --- | --- | --- | --- | --- | --- | --- | --- | --- | --- | --- | --- | --- | --- | --- | --- | --- | --- | --- | --- | --- | --- | --- | --- | --- | --- | --- | --- | --- | --- | --- | --- | --- | --- | --- | --- | --- | --- | --- | --- | --- | --- | --- | --- | --- | --- | --- | --- | --- | --- | --- | --- | --- | --- | --- | --- | --- | --- | --- | --- | --- | --- | --- | --- | --- | --- | --- | --- | --- | --- | --- | --- | --- | --- | --- | --- | --- | --- | --- | --- | --- | --- | --- | --- | --- | --- | --- | --- | --- | --- | --- | --- | --- | --- | --- | --- | --- | --- | --- | --- | --- | --- | --- | --- | --- | --- | --- | --- | --- | --- | --- | --- | --- | --- | --- | --- | --- | --- | --- | --- | --- | --- | --- | --- | --- | --- | --- | --- | --- | --- | --- | --- | --- | --- | --- | --- | --- | --- | --- | --- | --- | --- | --- | --- | --- | --- | --- | --- | --- | --- | --- | --- | --- | --- | --- | --- | --- | --- | --- | --- | --- | --- | --- | --- | --- | --- | --- | --- | --- | --- | --- | --- | --- | --- | --- | --- | --- | --- | --- | --- | --- | --- | --- | --- | --- | --- | --- | --- | --- | --- | --- | --- | --- | --- | --- | --- | --- | --- | --- | --- | --- | --- | --- | --- | --- | --- | --- | --- | --- | --- | --- | --- | --- | --- | --- | --- | --- | --- | --- | --- | --- | --- | --- | --- | --- | --- | --- | --- | --- | --- | --- | --- | --- | --- | --- | --- | --- | --- | --- | --- | --- | --- | --- | --- | --- | --- | --- | --- | --- | --- | --- | --- | --- | --- | --- | --- | --- | --- | --- | --- | --- | --- | --- | --- | --- | --- | --- | --- | --- | --- | --- | --- | --- | --- | --- | --- | --- | --- | --- | --- | --- | --- | --- | --- | --- | --- | --- | --- | --- | --- | --- | --- | --- | --- | --- | --- | --- | --- | --- | --- | --- | --- | --- | --- | --- | --- | --- | --- | --- | --- | --- | --- | --- | --- | --- | --- | --- | --- | --- | --- | --- | --- | --- | --- | --- | --- | --- | --- | --- | --- | --- | --- | --- | --- | --- | --- | --- | --- | --- | --- | --- | --- | --- | --- | --- | --- | --- | --- | --- | --- | --- | --- | --- | --- | --- | --- | --- | --- | --- | --- | --- | --- | --- | --- | --- | --- | --- | --- | --- | --- | --- | --- | --- | --- | --- | --- | --- | --- | --- | --- | --- | --- | --- | --- | --- | --- | --- | --- | --- | --- | --- | --- | --- | --- | --- | --- | --- | --- | --- | --- | --- | --- | --- | --- | --- | --- | --- | --- | --- | --- | --- | --- | --- | --- | --- | --- | --- | --- | --- | --- | --- | --- | --- | --- | --- | --- | --- | --- | --- | --- | --- | --- | --- | --- | --- | --- | --- | --- | --- | --- | --- | --- | --- | --- | --- | --- | --- | --- | --- | --- | --- | --- | --- | --- | --- | --- | --- | --- | --- | --- | --- | --- | --- | --- | --- | --- | --- | --- | --- | --- | --- | --- | --- | --- | --- | --- | --- | --- | --- | --- | --- | --- | --- | --- | --- | --- | --- | --- | --- | --- | --- | --- | --- | --- | --- | --- | --- | --- | --- | --- | --- | --- | --- | --- | --- | --- | --- | --- | --- | --- | --- | --- | --- | --- | --- | --- | --- | --- | --- | --- | --- | --- | --- | --- | --- | --- | --- | --- | --- | --- | --- | --- | --- | --- | --- | --- | --- | --- | --- | --- | --- | --- | --- | --- | --- | --- | --- | --- | --- | --- | --- | --- | --- | --- | --- | --- | --- | --- | --- | --- | --- | --- | --- | --- | --- | --- | --- | --- | --- | --- | --- | --- | --- | --- | --- | --- | --- | --- | --- | --- | --- | --- | --- | --- | --- | --- | --- | --- | --- | --- | --- | --- | --- | --- | --- | --- | --- | --- | --- | --- | --- | --- | --- | --- | --- | --- | --- | --- | --- | --- | --- | --- | --- | --- | --- | --- | --- | --- | --- | --- | --- | --- | --- | --- | --- | --- | --- | --- | --- | --- | --- | --- | --- | --- | --- | --- | --- | --- | --- | --- | --- | --- | --- | --- | --- | --- | --- | --- | --- | --- | --- | --- | --- | --- | --- | --- | --- | --- | --- | --- | --- | --- | --- | --- | --- | --- | --- | --- | --- | --- | --- | --- | --- | --- | --- | --- | --- | --- | --- | --- | --- | --- | --- | --- | --- | --- | --- | --- | --- | --- | --- | --- | --- | --- | --- | --- | --- | --- | --- | --- | --- | --- | --- | --- | --- | --- | --- | --- | --- | --- | --- | --- | --- | --- | --- | --- | --- | --- | --- | --- | --- | --- | --- | --- | --- | --- | --- | --- | --- | --- | --- | --- | --- | --- | --- | --- | --- | --- | --- | --- | --- | --- | --- | --- | --- | --- | --- | --- | --- | --- | --- | --- | --- | --- | --- | --- | --- | --- | --- | --- | --- | --- | --- | --- | --- | --- | --- | --- | --- | --- | --- | --- | --- | --- | --- | --- | --- | --- | --- | --- | --- | --- | --- | --- | --- | --- | --- | --- | --- | --- | --- | --- | --- | --- | --- | --- | --- | --- | --- | --- | --- | --- | --- | --- | --- | --- | --- | --- | --- | --- | --- | --- | --- | --- | --- | --- | --- | --- | --- | --- | --- | --- | --- | --- | --- | --- | --- | --- | --- | --- | --- | --- | --- | --- | --- | --- | --- | --- | --- | --- | --- | --- | --- | --- | --- | --- | --- | --- | --- | --- | --- | --- | --- | --- | --- | --- | --- | --- | --- | --- | --- | --- | --- | --- | --- | --- | --- | --- | --- | --- | --- | --- | --- | --- | --- | --- | --- | --- | --- | --- | --- | --- | --- | --- | --- | --- | --- | --- | --- | --- | --- | --- | --- | --- | --- | --- | --- | --- | --- | --- | --- | --- | --- | --- | --- | --- | --- | --- | --- | --- | --- | --- | --- | --- | --- | --- | --- | --- | --- | --- | --- | --- | --- | --- | --- | --- | --- | --- | --- | --- | --- | --- | --- | --- | --- | --- | --- | --- | --- | --- | --- | --- | --- | --- | --- | --- | --- | --- | --- | --- | --- | --- | --- | --- | --- | --- | --- | --- | --- | --- | --- | --- | --- | --- | --- | --- | --- | --- | --- | --- | --- | --- | --- | --- | --- | --- | --- | --- | --- | --- | --- | --- | --- | --- | --- | --- | --- | --- | --- | --- | --- | --- | --- | --- | --- | --- | --- | --- | --- | --- | --- | --- | --- | --- | --- | --- | --- | --- | --- | --- | --- | --- | --- | --- | --- | --- | --- | --- | --- | --- | --- | --- | --- | --- | --- | --- | --- | --- | --- | --- | --- | --- | --- | --- | --- | --- | --- | --- | --- | --- | --- | --- | --- | --- | --- | --- | --- | --- | --- | --- | --- | --- | --- | --- | --- | --- | --- | --- | --- | --- | --- | --- | --- | --- | --- | --- | --- | --- | --- | --- | --- | --- | --- | --- | --- | --- | --- | --- | --- | --- | --- | --- | --- | --- | --- | --- | --- | --- | --- | --- | --- | --- | --- | --- | --- | --- | --- | --- | --- | --- | --- | --- | --- | --- | --- | --- | --- | --- | --- | --- | --- | --- | --- | --- | --- | --- | --- | --- | --- | --- | --- | --- | --- | --- | --- | --- | --- | --- | --- | --- | --- | --- | --- | --- | --- | --- | --- | --- | --- | --- | --- | --- | --- | --- | --- | --- | --- | --- | --- | --- | --- | --- | --- | --- | --- | --- | --- | --- | --- | --- | --- | --- | --- | --- | --- | --- | --- | --- | --- | --- | --- | --- | --- | --- | --- | --- | --- | --- | --- | --- | --- | --- | --- | --- | --- | --- | --- | --- | --- | --- | --- | --- | --- | --- | --- | --- | --- | --- | --- | --- | --- | --- | --- | --- | --- | --- | --- | --- | --- | --- | --- | --- | --- | --- | --- | --- | --- | --- | --- | --- | --- | --- | --- | --- | --- | --- | --- | --- | --- | --- | --- | --- | --- | --- | --- | --- | --- | --- | --- | --- | --- | --- | --- | --- | --- | --- | --- | --- | --- | --- | --- | --- | --- | --- | --- | --- | --- | --- | --- | --- | --- | --- | --- | --- | --- | --- | --- | --- | --- | --- | --- | --- | --- | --- | --- | --- | --- | --- | --- | --- | --- | --- | --- | --- | --- | --- | --- | --- | --- | --- | --- | --- | --- | --- | --- | --- | --- | --- | --- | --- | --- | --- | --- | --- | --- | --- | --- | --- | --- | --- | --- | --- | --- | --- | --- | --- | --- | --- | --- | --- | --- | --- | --- | --- | --- | --- | --- | --- | --- | --- | --- | --- | --- | --- | --- | --- | --- | --- | --- | --- | --- | --- | --- | --- | --- | --- | --- | --- | --- | --- | --- | --- | --- | --- | --- | --- | --- | --- | --- | --- | --- | --- | --- | --- | --- | --- | --- | --- | --- | --- | --- | --- | --- | --- | --- | --- | --- | --- | --- | --- | --- | --- | --- | --- | --- | --- | --- | --- | --- | --- | --- | --- | --- | --- | --- | --- | --- | --- | --- | --- | --- | --- | --- | --- | --- | --- | --- | --- | --- | --- | --- | --- | --- | --- | --- | --- | --- | --- | --- | --- | --- | --- | --- | --- | --- | --- | --- | --- | --- | --- | --- | --- | --- | --- | --- | --- | --- | --- | --- | --- | --- | --- | --- | --- | --- | --- | --- | --- | --- | --- | --- | --- | --- | --- | --- | --- | --- | --- | --- | --- | --- | --- | --- | --- | --- | --- | --- | --- | --- | --- | --- | --- | --- | --- | --- | --- | --- | --- | --- | --- | --- | --- | --- | --- | --- | --- | --- | --- | --- | --- | --- | --- | --- | --- | --- | --- | --- | --- | --- | --- | --- | --- | --- | --- | --- | --- | --- | --- | --- | --- | --- | --- | --- | --- | --- | --- | --- | --- | --- | --- | --- | --- | --- | --- | --- | --- | --- | --- | --- | --- | --- | --- | --- | --- | --- | --- | --- | --- | --- | --- | --- | --- | --- | --- | --- | --- | --- | --- | --- | --- | --- | --- | --- | --- | --- | --- | --- | --- | --- | --- | --- | --- | --- | --- | --- | --- | --- | --- | --- | --- | --- | --- | --- | --- | --- | --- | --- | --- | --- | --- | --- | --- | --- | --- | --- | --- | --- | --- | --- | --- | --- | --- | --- | --- | --- | --- | --- | --- | --- | --- | --- | --- | --- | --- | --- | --- | --- | --- | --- | --- | --- | --- | --- | --- | --- | --- | --- | --- | --- | --- | --- | --- | --- | --- | --- | --- | --- | --- | --- | --- | --- | --- | --- | --- | --- | --- | --- | --- | --- | --- | --- | --- | --- | --- | --- | --- | --- | --- | --- | --- | --- | --- | --- | --- | --- | --- | --- | --- | --- | --- | --- | --- | --- | --- | --- | --- | --- | --- | --- | --- | --- | --- | --- | --- | --- | --- | --- | --- | --- | --- | --- | --- | --- | --- | --- | --- | --- | --- | --- | --- | --- | --- | --- | --- | --- | --- | --- | --- | --- | --- | --- | --- | --- | --- | --- | --- | --- | --- | --- | --- | --- | --- | --- | --- | --- | --- | --- | --- | --- | --- | --- | --- | --- | --- | --- | --- | --- | --- | --- | --- | --- | --- | --- | --- | --- | --- | --- | --- | --- | --- | --- | --- | --- | --- | --- | --- | --- | --- | --- | --- | --- | --- | --- | --- | --- | --- | --- | --- | --- | --- | --- | --- | --- | --- | --- | --- | --- | --- | --- | --- | --- | --- | --- | --- | --- | --- | --- | --- | --- | --- | --- | --- | --- | --- | --- | --- | --- | --- | --- | --- | --- | --- | --- | --- | --- | --- | --- | --- | --- | --- | --- | --- | --- | --- | --- | --- | --- | --- | --- | --- | --- | --- | --- | --- | --- | --- | --- | --- | --- | --- | --- | --- | --- | --- | --- | --- | --- | --- | --- | --- | --- | --- | --- | --- | --- | --- | --- | --- | --- | --- | --- | --- | --- | --- | --- | --- | --- | --- | --- | --- | --- | --- | --- | --- | --- | --- | --- | --- | --- | --- | --- | --- | --- | --- | --- | --- | --- | --- | --- | --- | --- | --- | --- | --- | --- | --- | --- | --- | --- | --- | --- | --- | --- | --- | --- | --- | --- | --- | --- | --- | --- | --- | --- | --- | --- | --- | --- | --- | --- | --- | --- | --- | --- | --- | --- | --- | --- | --- | --- | --- | --- | --- | --- | --- | --- | --- | --- | --- | --- | --- | --- | --- | --- | --- | --- | --- | --- | --- | --- | --- | --- | --- | --- | --- | --- | --- | --- | --- | --- | --- | --- | --- | --- | --- | --- | --- | --- | --- | --- | --- | --- | --- | --- | --- | --- | --- | --- | --- | --- | --- | --- | --- | --- | --- | --- | --- | --- | --- | --- | --- | --- | --- | --- | --- | --- | --- | --- | --- | --- | --- | --- | --- | --- | --- | --- | --- | --- | --- | --- | --- | --- | --- | --- | --- | --- | --- | --- | --- | --- | --- | --- | --- | --- | --- | --- | --- | --- | --- | --- | --- | --- | --- | --- | --- | --- | --- | --- | --- | --- | --- | --- | --- | --- | --- | --- | --- | --- | --- | --- | --- | --- | --- | --- | --- | --- | --- | --- | --- | --- | --- | --- | --- | --- | --- | --- | --- | --- | --- | --- | --- | --- | --- | --- | --- | --- | --- | --- | --- | --- | --- | --- | --- | --- | --- | --- | --- | --- | --- | --- | --- | --- | --- | --- | --- | --- | --- | --- | --- | --- | --- | --- | --- | --- | --- | --- | --- | --- | --- | --- | --- | --- | --- | --- | --- | --- | --- | --- | --- | --- | --- | --- | --- | --- | --- | --- | --- | --- | --- | --- | --- | --- | --- | --- | --- | --- | --- | --- | --- | --- | --- | --- | --- | --- | --- | --- | --- | --- | --- | --- | --- | --- | --- | --- | --- | --- | --- | --- | --- | --- | --- | --- | --- | --- | --- | --- | --- | --- | --- | --- | --- | --- | --- | --- | --- | --- | --- | --- | --- | --- | --- | --- | --- | --- | --- | --- | --- | --- | --- | --- | --- | --- | --- | --- | --- | --- | --- | --- | --- | --- | --- | --- | --- | --- | --- | --- | --- | --- | --- | --- | --- | --- | --- | --- | --- | --- | --- | --- | --- | --- | --- | --- | --- | --- | --- | --- | --- | --- | --- | --- | --- | --- | --- | --- | --- | --- | --- | --- | --- | --- | --- | --- | --- | --- | --- | --- | --- | --- | --- | --- | --- | --- | --- | --- | --- | --- | --- | --- | --- | --- | --- | --- | --- | --- | --- | --- | --- | --- | --- | --- | --- | --- | --- | --- | --- | --- | --- | --- | --- | --- | --- | --- | --- | --- | --- | --- | --- | --- | --- | --- | --- | --- | --- | --- | --- | --- | --- | --- | --- | --- | --- | --- | --- | --- | --- | --- | --- | --- | --- | --- | --- | --- | --- | --- | --- | --- | --- | --- | --- | --- | --- | --- | --- | --- | --- | --- | --- | --- | --- | --- | --- | --- | --- | --- | --- | --- | --- | --- | --- | --- | --- | --- | --- | --- | --- | --- | --- | --- | --- | --- | --- | --- | --- | --- | --- | --- | --- | --- | --- | --- | --- | --- | --- | --- | --- | --- | --- | --- | --- | --- | --- | --- | --- | --- | --- | --- | --- | --- | --- | --- | --- | --- | --- | --- | --- | --- | --- | --- | --- | --- | --- | --- | --- | --- | --- | --- | --- | --- | --- | --- | --- | --- | --- | --- | --- | --- | --- | --- | --- | --- | --- | --- | --- | --- | --- | --- | --- | --- | --- | --- | --- | --- | --- | --- | --- | --- | --- | --- | --- | --- | --- | --- | --- | --- | --- | --- | --- | --- | --- | --- | --- | --- | --- | --- | --- | --- | --- | --- | --- | --- | --- | --- | --- | --- | --- | --- | --- | --- | --- | --- | --- | --- | --- | --- | --- | --- | --- | --- | --- | --- | --- | --- | --- | --- | --- | --- | --- | --- | --- | --- | --- | --- | --- | --- | --- | --- | --- | --- | --- | --- | --- | --- | --- | --- | --- | --- | --- | --- | --- | --- | --- | --- | --- | --- | --- | --- | --- | --- | --- | --- | --- | --- | --- | --- | --- | --- | --- | --- | --- | --- | --- | --- | --- | --- | --- | --- | --- | --- | --- | --- | --- | --- | --- | --- | --- | --- | --- | --- | --- | --- | --- | --- | --- | --- | --- | --- | --- | --- | --- | --- | --- | --- | --- | --- | --- | --- | --- | --- | --- | --- | --- | --- | --- | --- | --- | --- | --- | --- | --- | --- | --- | --- | --- | --- | --- | --- | --- | --- | --- | --- | --- | --- | --- | --- | --- | --- | --- | --- | --- | --- | --- | --- | --- | --- | --- | --- | --- | --- | --- | --- | --- | --- | --- | --- | --- | --- | --- | --- | --- | --- | --- | --- | --- | --- | --- | --- | --- | --- | --- | --- | --- | --- | --- | --- | --- | --- | --- | --- | --- | --- | --- | --- | --- | --- | --- | --- | --- | --- | --- | --- | --- | --- | --- | --- | --- | --- | --- | --- | --- | --- | --- | --- | --- | --- | --- | --- | --- | --- | --- | --- | --- | --- | --- | --- | --- | --- | --- | --- | --- | --- | --- | --- | --- | --- | --- | --- | --- | --- | --- | --- | --- | --- | --- | --- | --- | --- | --- | --- | --- | --- | --- | --- | --- | --- | --- | --- | --- | --- | --- | --- | --- | --- | --- | --- | --- | --- | --- | --- | --- | --- | --- | --- | --- | --- | --- | --- | --- | --- | --- | --- | --- | --- | --- | --- | --- | --- | --- | --- | --- | --- | --- | --- | --- | --- | --- | --- | --- | --- | --- | --- | --- | --- | --- | --- | --- | --- | --- | --- | --- | --- | --- | --- | --- | --- | --- | --- | --- | --- | --- | --- | --- | --- | --- | --- | --- | --- | --- | --- | --- | --- | --- | --- | --- | --- | --- | --- | --- | --- | --- | --- | --- | --- | --- | --- | --- | --- | --- | --- | --- | --- | --- | --- | --- | --- | --- | --- | --- | --- | --- | --- | --- | --- | --- | --- | --- | --- | --- | --- | --- | --- | --- | --- | --- | --- | --- | --- | --- | --- | --- | --- | --- | --- | --- | --- | --- | --- | --- | --- | --- | --- | --- | --- | --- | --- | --- | --- | --- | --- | --- | --- | --- | --- | --- | --- | --- | --- | --- | --- | --- | --- | --- | --- | --- | --- | --- | --- | --- | --- | --- | --- | --- | --- | --- | --- | --- | --- | --- | --- | --- | --- | --- | --- | --- | --- | --- | --- | --- | --- | --- | --- | --- | --- | --- | --- | --- | --- | --- | --- | --- | --- | --- | --- | --- | --- | --- | --- | --- | --- | --- | --- | --- | --- | --- | --- | --- | --- | --- | --- | --- | --- | --- | --- | --- | --- | --- | --- | --- | --- | --- | --- | --- | --- | --- | --- | --- | --- | --- | --- | --- | --- | --- | --- | --- | --- | --- | --- | --- | --- | --- | --- | --- | --- | --- | --- | --- | --- | --- | --- | --- | --- | --- | --- | --- | --- | --- | --- | --- | --- | --- | --- | --- | --- | --- | --- | --- | --- | --- | --- | --- | --- | --- | --- | --- | --- | --- | --- | --- | --- | --- | --- | --- | --- | --- | --- | --- | --- | --- | --- | --- | --- | --- | --- | --- | --- | --- | --- | --- | --- | --- | --- | --- | --- | --- | --- | --- | --- | --- | --- | --- | --- | --- | --- | --- | --- | --- | --- | --- | --- | --- | --- | --- | --- | --- | --- | --- | --- | --- | --- | --- | --- | --- | --- | --- | --- | --- | --- | --- | --- | --- | --- | --- | --- | --- | --- | --- | --- | --- | --- | --- | --- | --- | --- | --- | --- | --- | --- | --- | --- | --- | --- | --- | --- | --- | --- | --- | --- | --- | --- | --- | --- | --- | --- | --- | --- | --- | --- | --- | --- | --- | --- | --- | --- | --- | --- | --- | --- | --- | --- | --- | --- | --- | --- | --- | --- | --- | --- | --- | --- | --- | --- | --- | --- | --- | --- | --- | --- | --- | --- | --- | --- | --- | --- | --- | --- | --- | --- | --- | --- | --- | --- | --- | --- | --- | --- | --- | --- | --- | --- | --- | --- | --- | --- | --- | --- | --- | --- | --- | --- | --- | --- | --- | --- | --- | --- | --- | --- | --- | --- | --- | --- | --- | --- | --- | --- | --- | --- | --- | --- | --- | --- | --- | --- | --- | --- | --- | --- | --- | --- | --- | --- | --- | --- | --- | --- | --- | --- | --- | --- | --- | --- | --- | --- | --- | --- | --- | --- | --- | --- | --- | --- | --- | --- | --- | --- | --- | --- | --- | --- | --- | --- | --- | --- | --- | --- | --- | --- | --- | --- | --- | --- | --- | --- | --- | --- | --- | --- | --- | --- | --- | --- | --- | --- | --- | --- | --- | --- | --- | --- | --- | --- | --- | --- | --- | --- | --- | --- | --- | --- | --- | --- | --- | --- | --- | --- | --- | --- | --- | --- | --- | --- | --- | --- | --- | --- | --- | --- | --- | --- | --- | --- | --- | --- | --- | --- | --- | --- | --- | --- | --- | --- | --- | --- | --- | --- | --- | --- | --- | --- | --- | --- | --- | --- | --- | --- | --- | --- | --- | --- | --- | --- | --- | --- | --- | --- | --- | --- | --- | --- | --- | --- | --- | --- | --- | --- | --- | --- | --- | --- | --- | --- | --- | --- | --- | --- | --- | --- | --- | --- | --- | --- | --- | --- | --- | --- | --- | --- | --- | --- | --- | --- | --- | --- | --- | --- | --- | --- | --- | --- | --- | --- | --- | --- | --- | --- | --- | --- | --- | --- | --- | --- | --- | --- | --- | --- | --- | --- | --- | --- | --- | --- | --- | --- | --- | --- | --- | --- | --- | --- | --- | --- | --- | --- | --- | --- | --- | --- | --- | --- | --- | --- | --- | --- | --- | --- | --- | --- | --- | --- | --- | --- | --- | --- | --- | --- | --- | --- | --- | --- | --- | --- | --- | --- | --- | --- | --- | --- | --- | --- | --- | --- | --- | --- | --- | --- | --- | --- | --- | --- | --- | --- | --- | --- | --- | --- | --- | --- | --- | --- | --- | --- | --- | --- | --- | --- | --- | --- | --- | --- | --- | --- | --- | --- | --- | --- | --- | --- | --- | --- | --- | --- | --- | --- | --- | --- | --- | --- | --- | --- | --- | --- | --- | --- | --- | --- | --- | --- | --- | --- | --- | --- | --- | --- | --- | --- | --- | --- | --- | --- | --- | --- | --- | --- | --- | --- | --- | --- | --- | --- | --- | --- | --- | --- | --- | --- | --- | --- | --- | --- | --- | --- | --- | --- | --- | --- | --- | --- | --- | --- | --- | --- | --- | --- | --- | --- | --- | --- | --- | --- | --- | --- | --- | --- | --- | --- | --- | --- | --- | --- | --- | --- | --- | --- | --- | --- | --- | --- | --- | --- | --- | --- | --- | --- | --- | --- | --- | --- | --- | --- | --- | --- | --- | --- | --- | --- | --- | --- | --- | --- | --- | --- | --- | --- | --- | --- | --- | --- | --- | --- | --- | --- | --- | --- | --- | --- | --- | --- | --- | --- | --- | --- | --- | --- | --- | --- | --- | --- | --- | --- | --- | --- | --- | --- | --- | --- | --- | --- | --- | --- | --- | --- | --- | --- | --- | --- | --- | --- | --- | --- | --- | --- | --- | --- | --- | --- | --- | --- | --- | --- | --- | --- | --- | --- | --- | --- | --- | --- | --- | --- | --- | --- | --- | --- | --- | --- | --- | --- | --- | --- | --- | --- | --- | --- | --- | --- | --- | --- | --- | --- | --- | --- | --- | --- | --- | --- | --- | --- | --- | --- | --- | --- | --- | --- | --- | --- | --- | --- | --- | --- | --- | --- | --- | --- | --- | --- | --- | --- | --- | --- | --- | --- | --- | --- | --- | --- | --- | --- | --- | --- | --- | --- | --- | --- | --- | --- | --- | --- | --- | --- | --- | --- | --- | --- | --- | --- | --- | --- | --- | --- | --- | --- | --- | --- | --- | --- | --- | --- | --- | --- | --- | --- | --- | --- | --- | --- | --- | --- | --- | --- | --- | --- | --- | --- | --- | --- | --- | --- | --- | --- | --- | --- | --- | --- | --- | --- | --- | --- | --- | --- | --- | --- | --- | --- | --- | --- | --- | --- | --- | --- | --- | --- | --- | --- | --- | --- | --- | --- | --- | --- | --- | --- | --- | --- | --- | --- | --- | --- | --- | --- | --- | --- | --- | --- | --- | --- | --- | --- | --- | --- | --- | --- | --- | --- | --- | --- | --- | --- | --- | --- | --- | --- | --- | --- | --- | --- | --- | --- | --- | --- | --- | --- | --- | --- | --- | --- | --- | --- | --- | --- | --- | --- | --- | --- | --- | --- | --- | --- | --- | --- | --- | --- | --- | --- | --- | --- | --- | --- | --- | --- | --- | --- | --- | --- | --- | --- | --- | --- | --- | --- | --- | --- | --- | --- | --- | --- | --- | --- | --- | --- | --- | --- | --- | --- | --- | --- | --- | --- | --- | --- | --- | --- | --- | --- | --- | --- | --- | --- | --- | --- | --- | --- | --- | --- | --- | --- | --- | --- | --- | --- | --- | --- | --- | --- | --- | --- | --- | --- | --- | --- | --- | --- | --- | --- | --- | --- | --- | --- | --- | --- | --- | --- | --- | --- | --- | --- | --- | --- | --- | --- | --- | --- | --- | --- | --- | --- | --- | --- | --- | --- | --- | --- | --- | --- | --- | --- | --- | --- | --- | --- | --- | --- | --- | --- | --- | --- | --- | --- | --- | --- | --- | --- |
