# Supplementary material for: Transcriptional profiling of leukocytes in critically ill COVID19 patients: implications for interferon response and coagulation
Source: Intensive Care Med Exp. 2020 Dec 11;8:75. doi: 10.1186/s40635-020-00361-9 (PMC7729690; doi:10.1186/s40635-020-00361-9)
Supplement: Supplementary file 4 — Additional file 4: Table S2. Top significant cell types from Enrichr (p < 0.05) [file 40635_2020_361_MOESM4_ESM.docx]

**Supplemental Table 2: Top significant cell types**

**from Enrichr (p < 0.05).**

| Cell types | p-value |
| --- | --- |
| CD14+ Monocytes | 5.19E-22 |
| CD33+ Myeloid | 3.62E-14 |
| BDCA4+ Dendritic Cells | 1.45E-06 |
| CD8+ T cells | 2.76E-04 |
| CD4+ T cells | 3.27E-04 |
